# Supplementary material for: Are workplace factors associated with employee alcohol use? The WIRUS cross-sectional study
Source: BMJ Open. 2022 Oct 13;12(10):e064352. doi: 10.1136/bmjopen-2022-064352 (PMC9562323; doi:10.1136/bmjopen-2022-064352)
Supplement: Supplementary data [file bmjopen-2022-064352supp001.pdf]

Supplementary File

Table S1. Matrix of bivariate correlations between all study variables (outcomes, predictors and covariates)

	1	2	3	4	5	6	7	8	9	10	11	12	13	14	15	16	17	18	19	20	21	22	23	24	25
1. Alcohol consumption																									
2. Alcohol-rel. problems[A]	**.49**																								
3. Working hours	.02	-.01																							
4. Day job, no weekends[B]	**-.05**	**-.05**	.02																						
5. Evening job[B]	.01	.02	**-.05**	**-.11**																					
6. Night job[B]	-.01	.01	-.01	**-.21**	-.01																				
7. Shift work[B]	**.04**	**.04**	**-.08**	**-.79**	**-.03**	**-.06**																			
8. Job position[C]	**.04**	**-.03**	**.24**	**.12**	**-.03**	**-.06**	**-.14**																		
9. Job size[D]	**.08**	**.04**	**.25**	**.20**	**-.10**	**-.25**	**-.15**	**.19**																	
10. Income	.01	**-.08**	**.14**	**.13**	**-.04**	**-.05**	**-.13**	**.21**	**.11**																
11. Psych. job demands	-.02	-.00	**.23**	**-.05**	-.00	**-.06**	.03	**.17**	**.07**	**.05**															
12. Job control	-.01	**-.04**	**.15**	**.10**	-.01	**-.08**	**-.12**	**.14**	**.08**	**.17**	**.14**														
13. Work. social support	.01	**-.03**	.00	**.06**	-.01	**-.03**	**-.05**	.00	-.00	**.05**	**-.06**	**.34**													
14. Leadership qualities	-.02	**-.03**	-.02	.02	-.01	**-.04**	-.01	-.01	-.01	.01	**-.10**	**.23**	**.61**												
15. Job travels[E]	**.07**	.02	**.13**	**.09**	-.02	**-.05**	**-.11**	**.16**	**.13**	**.15**	**-05**	**.14**	.01	-.01											
16. Working from home[E]	.02	-.00	**.12**	**.04**	-.01	**-.03**	**-.09**	**.11**	**.04**	**.10**	**.06**	**.13**	.01	-.01	**.25**										
17. Work. holiday home[E]	**.06**	.01	**.12**	-.03	-.01	-.02	-.02	**.10**	**.05**	**.11**	**.07**	**.06**	-.02	-.02	**.24**	**.32**									
18. Drinking social norms[F]	**.42**	**.23**	**.06**	**-.05**	.02	-.03	**.04**	**-.05**	**.07**	.02	-.01	-.01	.00	.01	**.09**	**.06**	**.03**								
19. Gender[G]	**-.18**	**-.14**	**-.16**	**.04**	.01	.02	-.02	**-.13**	**-.17**	-.02	**.05**	.01	**.06**	**.05**	**-.17**	**-.03**	**-.08**	**-.16**							
20. Age	**-.12**	**-.13**	**.05**	**.19**	-.01	-.01	**-.20**	**.18**	.00	**.10**	**-.07**	-.02	**-.09**	**-.10**	.03	-.02	**.05**	**-.25**	**-.04**						
21. Educational attainment	**-.03**	-.03	**.18**	**.15**	-.03	**-.07**	**-.18**	**.09**	**.13**	**.29**	**.10**	**.30**	**.08**	**.04**	**.16**	**.14**	**.06**	**.06**	.01	**-.07**					
22. Living status[H]	**-.07**	**-.12**	.02	**.07**	-.01	-.02	**-.06**	**.06**	-.02	**.50**	.01	**.07**	**.03**	.03	**.03**	.01	**.03**	**-.04**	-.01	-.02	**.03**				
23. Marital status[I]	**-.14**	**-.15**	**.04**	**.14**	-.01	-.02	**-.15**	**.13**	-.02	**.46**	.00	**.08**	.01	-.00	**.06**	**.03**	**.04**	**-.12**	**-.04**	**.26**	**.07**	**.46**			
24. Number of children	**-.16**	**-.17**	**.03**	**.15**	-.02	-.01	**-.15**	**.14**	-.02	**.31**	-.01	**.07**	-.01	-.01	**.06**	**.04**	**.04**	**-.20**	.00	**.40**	.01	**.33**	**.42**		
25. Year of data collection	.01	.01	**-.10**	**-.12**	**.04**	**.04**	**.16**	.00	**-.04**	**-.07**	.02	**.06**	**.07**	**.09**	**-.15**	**-.06**	-.01	**-.04**	**.14**	**-.07**	**.04**	-.02	**-.03**	-.02	

N = 5388; Results from non-parametric (Spearman *rho*) correlation analyses; Statistically significant correlations (p <.05) indicated with bold typeface; [A]Ref. = no problems; [B]Ref. = other work schedules; [C]Ref. = worker; [D]Ref. = part-time; [E]Ref. = no; [F]Higher scores indicate liberal attitudes; [G]Ref. = male; [H]Ref. = living alone; [I]Ref. = unmarried; For all other variables: higher scores indicate higher variable values

Table S2. Study selection analyses: Comparisons between completers and non-completers on sociodemographic variables

Variable	Completers[A]	Non-completers[B]	*p*
Gender, % females	68.5	75.7	<.001[C]
Age, *M*	44.8	44.7	.946[D]
Education, % university/college	77.7	66.2	<.001[C]
Livings status, % living alone	15.1	15.1	.949[C]
Marital status, % unmarried	44.8	48.0	.005[C]
Children, *M*	1.8	1.7	.001[D]

[A]Employees who responded on all study items, i.e., the study sample (n = 5388); [B]Employees who responded on the sociodemographic items but not on all study items; [C]Difference tested with chi square tests of independence; [D]Difference tested with independent samples t-tests
