# Supplementary material for: Are workplace factors associated with employee alcohol use? The WIRUS cross-sectional study
Source: BMJ Open. 2022 Oct 13;12(10):e064352. doi: 10.1136/bmjopen-2022-064352 (PMC9562323; doi:10.1136/bmjopen-2022-064352)
Supplement: Author's manuscript [file bmjopen-2022-064352.draft_revisions.pdf]

BMJ Open is committed to open peer review. As part of this commitment we make the peer review history of every article we publish publicly available.

When an article is published we post the peer reviewers' comments and the authors' responses online. We also post the versions of the paper that were used during peer review. These are the versions that the peer review comments apply to.

The versions of the paper that follow are the versions that were submitted during the peer review process. They are not the versions of record or the final published versions. They should not be cited or distributed as the published version of this manuscript.

BMJ Open is an open access journal and the full, final, typeset and author-corrected version of record of the manuscript is available on our site with no access controls, subscription charges or pay-per-view fees (http://bmjopen.bmj.com).

If you have any questions on BMJ Open's open peer review process please email info.bmjopen@bmj.com

Associations between workplace factors and employee alcohol use: the WIRUS Screening study

Journal:	*BMJ Open*
Manuscript ID	bmjopen-2022-064352
Article Type:	Original research
Date Submitted by the Author:	30-Apr-2022

Complete List of Authors:	Thørrisen, Mikkel; Oslo Metropolitan University, Department of Occupational Therapy, Prosthetics and Orthotics; University of Stavanger, Department of Public Health<br>Skogen, Jens; Nasjonalt folkehelseinstitutt, Department of Health Promotion; Stavanger University Hospital<br>Bonsaksen, Tore; Inland Norway University of Applied Sciences; VID Specialized University - Sandness<br>Skarpaas, Lisebet; Oslo Metropolitan University, Department of Occupational Therapy, Prosthetics and Orthotics<br>Aas, Randi; Oslo Metropolitan University, Department of Occupational Therapy, Prosthetics and Orthotics; University of Stavanger, Department of Public Health

Keywords:	OCCUPATIONAL & INDUSTRIAL MEDICINE, PUBLIC HEALTH, EPIDEMIOLOGY

SCHOLARONE™ Manuscripts

I, the Submitting Author has the right to grant and does grant on behalf of all authors of the Work (as defined in the below author licence), an exclusive licence and/or a non-exclusive licence for contributions from authors who are: i) UK Crown employees; ii) where BMJ has agreed a CC-BY licence shall apply, and/or iii) in accordance with the terms applicable for US Federal Government officers or employees acting as part of their official duties; on a worldwide, perpetual, irrevocable, royalty-free basis to BMJ Publishing Group Ltd ("BMJ") its licensees and where the relevant Journal is co-owned by BMJ to the co-owners of the Journal, to publish the Work in this journal and any other BMJ products and to exploit all rights, as set out in our licence.

The Submitting Author accepts and understands that any supply made under these terms is made by BMJ to the Submitting Author unless you are acting as an employee on behalf of your employer or a postgraduate student of an affiliated institution which is paying any applicable article publishing charge ("APC") for Open Access articles. Where the Submitting Author wishes to make the Work available on an Open Access basis (and intends to pay the relevant APC), the terms of reuse of such Open Access shall be governed by a Creative Commons licence – details of these licences and which Creative Commons licence will apply to this Work are set out in our licence referred to above.

Other than as permitted in any relevant BMJ Author's Self Archiving Policies, I confirm this Work has not been accepted for publication elsewhere, is not being considered for publication elsewhere and does not duplicate material already published. I confirm all authors consent to publication of this Work and authorise the granting of this licence.

For peer review only

Associations between workplace factors and employee alcohol use: the WIRUS Screening study

Mikkel Magnus Thørrisen[1,2]*
Jens Christoffer Skogen[2,3,4]
Tore Bonsaksen[5,6]
Lisebet Skeie Skarpaas[1]
Randi Wågø Aas[1,2]*

[1]Department of Occupational Therapy, Prosthetics and Orthotics, Faculty of Health Sciences, OsloMet – Oslo Metropolitan University, Oslo, Norway

[2]Department of Public Health, Faculty of Health Sciences, University of Stavanger, Stavanger, Norway

[3]Department of Health Promotion, Norwegian Institute of Public Health, Bergen, Norway

[4]Center for Alcohol & Drug Research, Stavanger University Hospital, Stavanger, Norway

[5]Department of Health and Nursing Sciences, Faculty of Social and Health Sciences, Inland Norway University of Applied Sciences, Elverum, Norway

[6]Department of Health, Faculty of Health Studies, VID Specialized University, Sandnes, Norway

*Correspondence:
Mikkel Magnus Thørrisen / Randi Wågø Aas

OsloMet – Oslo Metropolitan University, Faculty of Health Sciences, Department of Occupational Therapy, Prosthetics and Orthotics, PO box 4 St. Olavs plass, NO-0130 Oslo, Norway

mikkel-magnus.thorrisen@oslomet.no / randi.aas@oslomet.no

Word count: 4752 (main text, i.e., excluding title page, abstract, strength/limitations statement, declarations, reference list, tables, figure and supplementary material)

For peer review only - http://bmjopen.bmj.com/site/about/guidelines.xhtml

For peer review only

ABSTRACT

Objectives: Reducing harmful alcohol use is pivotal in the sustainable development of health. Sociodemographic predictors of employee alcohol use are well established in the literature, but knowledge about associations between workplace factors and alcohol use is less explored, with inconclusive results. The aim of this study was to explore whether workplace factors were associated with employee alcohol use (consumption and alcohol-related problems).

Design: Cross-sectional study. Linear and binary logistic regression analyses were performed.

Setting: Heterogeneous sample of employees (workers and supervisors) from 22 companies across geographical locations and work divisions in Norway.

Participants: Employees (N = 6164) responded on survey items measuring workplace factors (working hours, work schedule, working from home/holiday home, job travels, job position, job size, supervisors' leadership qualities, psychological job demands, job control, workplace social support) and alcohol use.

Outcomes: Data on alcohol use were collected with the Alcohol Use Disorders Identification Test (AUDIT). Consumption was measured with the AUDIT-C (the first three items), and alcohol-related problems were operationalised as a sum score of eight or higher on the full ten-item AUDIT.

Results Higher levels of alcohol consumption was associated with working from holiday home (b = .53, p <.001), job travels (b = .28, p <.001), working full-time (b = .28, p <.001) and being a supervisor (b = .27, p <.001). Alcohol-related problems was associated with lower scores on supervisors' desired leadership qualities (OR = 0.85, p = .002) and lower workplace social support (OR = 0.81, p = .016).

Conclusions: Results suggest that some workplace factors may play a role in explaining employee alcohol consumption, although the predictive ability of these factors were limited.

For peer review only - http://bmjopen.bmj.com/site/about/guidelines.xhtml

For peer review only

This study points to the importance of desired leadership qualities and workplace social support as potential protective factors for alcohol-related problems among employees.

Keywords: Alcohol Drinking; Employees; Work; Workplace

Strengths and limitations of this study

- This study explores the importance of workplace factors for employee alcohol use in a large and heterogeneous sample by means of a validated alcohol screening instrument
- This study utilises individuals' overall degree of consumption, as well as odds of having a drinking pattern that may induce alcohol-related problems, as outcomes
- Study limitations include a low response rate, the use of self-reported data, and a cross-sectional design that precludes causal inferences

INTRODUCTION

Alcohol consumption is associated with detrimental outcomes such as disease, disability and mortality.[1-4] Globally, approximately five percent of mortality and disability-adjusted life years can be attributed to alcohol consumption.[5] Within the age group of 25-49 years, 1.6 % of global disability-adjusted-life-years (DALYs) were attributable to alcohol use disorders in 2019.[6] Reducing harmful alcohol use represents a keystone in sustainable development of health,[5, 7] and risky drinking has been conceptualised as a drinking pattern that increases the risk of social, legal, medical, occupational, domestic and economic problems.[8]

Alcohol is the most commonly used and misused psychoactive substance in the workforce.[9] Between 10 and 30 % of employees consume alcohol at a risky level and may benefit from alcohol prevention interventions.[10] Employees' alcohol use is associated with

For peer review only - http://bmjopen.bmj.com/site/about/guidelines.xhtml

increased sickness absence,[11-14] and presenteeism (impaired on-the-job performance).[15, 16] Research has demonstrated that brief alcohol interventions have promising effects in primary care,[17, 18] as well as in working populations.[19-21]

According to Frone's model of employee substance use,[22] employee alcohol use can be construed as a function of individual and workplace environment characteristics, and multifaceted interactions between them. Individual (sociodemographic) predictors of alcohol use among employees are quite well established in the research literature. For instance, being male, young, unmarried and not having children constitute individual predictors of elevated alcohol consumption.[10, 23, 24] The role of workplace factors is, however, not as established in the literature. Workplace factors may, on the one hand, refer to the workplace as a sociocultural setting (e.g., with a specific drinking culture), or to more general workplace factors conceptualised in the form of conditions and terms defining the work situation (e.g., work schedule, work location, job position, job size and psychosocial work environment). Studies have demonstrated that workplace drinking culture may impact the drinking level of employees.[25-27] Research on the importance of other aspects of the workplace and work situation stands out as more inconclusive.

Systematic reviews generally point to longer working hours being associated with higher alcohol consumption.[28, 29] However, the evidence for such a relationship remains uncertain. Some studies have failed to demonstrate statistically significant relationships,[30-32] and an opposite association (shorter working hours associated with higher risk of alcohol dependence) was found in a study of employees in Taiwan.[33] In a Norwegian study,[34] having flexible working hours was associated with increased consumption, and some studies have linked shift work with increased probability of workplace alcohol use,[30] as well as with higher overall consumption.[35] Others have not found a relationship between shift work and alcohol use.[34, 36-38] In a study of employees in Norway,[39] working from home

For peer review only - http://bmjopen.bmj.com/site/about/guidelines.xhtml

(telecommuting) at least two days per week was associated with higher alcohol use. Furthermore, a high prevalence of job travels has been linked with increased likelihood of problematic drinking.[40]

Evidence on the importance of work position is mixed. Some studies support an association between being a supervisor (as opposed to a worker) and higher alcohol consumption,[31, 39] while others do not.[23, 30, 33, 34] In a study of employees in the USA, Lee et al. revealed that working part-time (as opposed to full-time) was associated with higher alcohol use per week, but unrelated to prevalence of binge drinking episodes and alcohol-related consequences.[41] Although working hours may be somewhat overlapping with job size, one may capture different aspects of "time at work" by studying typical number of hours at work per day (working hours) and whether or not an individual has a full-time job (job-size). For instance, variations in working hours that exceed "normal working hours" (e.g., overtime) will not be captured in a dichotomy of part-time and full-time work.

Research on the importance of the psychosocial work environment (e.g., leadership, job demands and control, and workplace social support) for employee alcohol use have yielded variable results. It has been advocated that visible and active supervisors constitute a protective factor for alcohol use among workers,[42] while others have failed to demonstrate such a relationship.[40, 43] In a Norwegian study,[44] supervisors' leadership style (transformational and laissez-fair leadership) were not related to employee alcohol use. On the other hand, some studies imply that alcohol use and hangover episodes are less prevalent at workplaces where supervisors exert active social control.[25, 45, 46]

Analyses of cross-sectional data imply that lower levels of job control (skill discretion and decision latitude) may be associated with higher levels of alcohol consumption, although such a relationship has proved difficult to replicate in longitudinal data.[47] Some studies reject a potential association between job control and alcohol use,[23, 33, 48] while others underscore

For peer review only - http://bmjopen.bmj.com/site/about/guidelines.xhtml

that different types of control may be differentially related to consumption. For instance, Marchand found that an increased probability of risky drinking was associated with lower skill discretion and higher decision latitude,[31] while Saade and Marchand found the opposite.[32] Higher perceived psychological job demands have in some studies been related to higher alcohol use,[32, 33] but not in others.[49] In a study of Canadian employees,[50] lower psychological demands were associated with higher alcohol use for men only. The evidence base of physical job demands is also inconclusive. A Canadian study found that higher physical demands were associated with higher alcohol use among men only,[50] while a French study found an association for women only.[49] Another Canadian study failed to demonstrate a relationship between physical job demands and alcohol use altogether.[32] Some studies indicate that lower workplace social support is associated with higher alcohol consumption,[32, 37, 50] while others do not.[23, 48] A study from Norway found increased odds of alcohol-related problems among employees who reported a perceived imbalance between work efforts and rewards, in conjunction with high levels of work overcommitment.[51]

In summary, evidence on associations between workplace factors and employee alcohol use stands out as mixed and inconclusive. Moreover, few studies have explored the importance of workplace factors for alcohol consumption as well as for odds of alcohol-related problems in heterogeneous employee samples by means of validated alcohol screening instruments. Individuals' overall degree of consumption, and their odds of having a drinking pattern that may induce alcohol-related problems, constitute two different – yet equally important – ways of conceptualising alcohol use that can be differentially associated with workplace factors. Knowledge about associations between workplace factors and employee alcohol use is important as an aid in determining the extent to which employers should make workplace-based alcohol health promotion and prevention interventions an overall priority,

For peer review only - http://bmjopen.bmj.com/site/about/guidelines.xhtml

and for determining which elements should be emphasised in such interventions. Hence, further research is warranted.

Study aim

The aim of this study was to explore whether workplace factors (working hours, work schedule, working from home/holiday home, job travels, job position, job size, supervisors' leadership qualities, psychological job demands, job control, workplace social support) were associated with employee alcohol consumption and odds having of alcohol-related problems in a heterogeneous sample of employees in Norway.

MATERIALS AND METHODS

Design and setting

This cross-sectional study explored associations between workplace factors and alcohol use in a heterogeneous sample of employees (workers and supervisors) in 22 companies in Norway (private companies: n = 8; central government companies: n = 6; local government companies: n = 8), across geographical locations. The study was part of the Norwegian national WIRUS project (Workplace Interventions preventing Risky alcohol Use and Sick leave).

Data collection and sample

The researchers recruited companies through three occupational health service units' client lists in collaboration with the addiction competence environment KoRus Stavanger. Individual-level inclusion criteria were: (i) age 16-72; (ii) status as employee (blue, white or pink collar worker, or supervisor, i.e., salaried person), regardless of sector, work division or geographical location; and (iii) basic understanding of the Norwegian language. The invited

For peer review only - http://bmjopen.bmj.com/site/about/guidelines.xhtml

sample was, in accordance with the inclusion criteria, heterogeneous, representing different sectors, work divisions and geographical locations. Twenty-two companies agreed to participate and provided e-mail addresses for all their employees (n = 30811). Digital questionnaires measuring workplace factors, alcohol use and sociodemographic variables were distributed to all employees in the 22 companies. Consent to participate was provided by 8542 employees (response rate = 27.7 %). A total of 2378 employees failed to respond on all relevant study items, leaving a final study sample of 6164 employees. Characteristics of the study sample are presented in Table 1.

Table 1. Characteristics of the study sample

Variable	
**Gender**	
Male, n (%)	1825 (29.6)
Female, n (%)	4339 (70.4)
**Age** (years), *M* (*SD*)	44.8 (11.3)
**Educational attainment**	
Primary/lower secondary, n (%)	115 (1.9)
Upper secondary, n (%)	1247 (20.2)
University/college ≤4 years, n (%)	2411 (39.1)
University/college >4 years, n (%)	2391 (38.8)
**Employment sector**	
Private corporation, n (%)	429 (7.0)
Local government, n (%)	4028 (65.3)
Central government, n (%)	1707 (27.7)
**Work division**	
Transportation/manufacturing, n (%)	310 (5.0)
Public administration/services, n (%)	4914 (79.7)
Health services, n (%)	874 (14.2)
Other services, n (%)	66 (1.1)

N = 6164; *M* = mean; *SD* = standard deviation

Data from participants were collected between October 2014 and October 2019. Hence, each participant was measured once (between 2014 and 2019), and all data were collected prior to the covid-19 pandemic. We predefined that a statistically satisfactory sample size had to

For peer review only - http://bmjopen.bmj.com/site/about/guidelines.xhtml

conform with the formula N > 50 + (8 x number of predictors/covariates),[52] as well as exceeding a ratio of 15 participants per predictor/covariate variable.[53]

Measures

Employee alcohol use (outcomes)

The outcomes were measured with the Alcohol Use Disorders Identification Test (AUDIT). The AUDIT is an alcohol screening instrument, developed by the World Health Organization (WHO),[8, 54] consisting of 10 items (each scored 0-4). Higher scores on the AUDIT indicate higher consumption and more severe alcohol-related consequences. The first outcome, *alcohol consumption*, was measured with the first three AUDIT items (sum score of three items = continuous scale 0-12), often referred to as the AUDIT-C.[55] The second outcome, *alcohol-related problems*, was measured as a dichotomous categorical variable (0 = no problems; 1 = problems), based on a cut-off of eight on the full ten-item AUDIT (scores 0-7 = no problems; scores 8-40 = problems).[8]

Workplace factors (predictors)

Work schedule factors included *working hours* (number of hours during a typical day) and *work schedule* (day job without weekends; day job with weekends; evening job; night job; shift work). Work location flexibility were measured in terms of whether the job included *working from home* (no; yes), *working from holiday home* (no; yes) and *job travels* (no; yes). Employees also provided information about their *job position* (worker; supervisor) and their *job size* (part-time; full-time).

Factors related to the psychosocial work environment included workers' perceptions of supervisors' leadership qualities, psychological job demands, job control and workplace social support. *Supervisors' leadership qualities* were measured with the Seven-item Leadership

For peer review only - http://bmjopen.bmj.com/site/about/guidelines.xhtml

Qualities Questionnaire (7LQQ).[56] Developed on the basis of qualitative interview data,[57] 78 distinct leadership qualities were identified and categorised into seven desired leadership types (problem-solver; contact-maker; responsible-maker; protector; trust-creator; recogniser; encourager), each type scored on a five-point Likert scale based on the frequency of which supervisors were perceived to display the leadership type in question (0 = never; 1 = seldom; 2 = sometimes; 3 = often; 4 = very often). A mean score of the seven items was calculated (α = .94), where higher scores indicated higher levels of desired leadership qualities.

Psychological job demands (five items), *job control* (perceived control over workplace decisions and potential utilising personal skills in the job; nine items) and *workplace social support* (from co-workers and supervisors; eight items) were measured with the Job Content Questionnaire (JCQ),[58] based on the Demand Control Support model,[59, 60] each scored on four-point Likert scales where higher scored indicated higher levels of demands, control and support, respectively. Mean scores were calculated (demands: α = .76; control: α = .58; support: α = .89).

An overview of workplace factors is presented in Table 2.

Table 2. Overview of workplace factors (predictors)

Main factor	Factors	Measurement
Work schedule	Working hours	Continuous[A]: number of hours during a typical day
	Work schedule	Categorical[A]: day job, no weekends; day job with weekends; evening job; night job; shift work
Work location flexibility	Working from home	Categorical[A]: no; yes
	Working from holiday home	Categorical[A]: no; yes
	Job travels	Categorical[A]: no; yes
Job position and job size	Job position	Categorical[A]: worker; supervisor
	Job size	Categorical[A]: part-time; full-time
Psychosocial work environment	Supervisors' leadership qualities	7LQQ: mean score of seven items
	Psychological job demands	JCQ: mean score of five items
	Job control	JCQ: mean score of nine items

For peer review only - http://bmjopen.bmj.com/site/about/guidelines.xhtml

Workplace social support	JCQ: mean score of eight items

[A]Self-developed measure; 7LQQ = Seven-item Leadership Qualities Questionnaire; JCQ = Job Content Questionnaire

Covariates

The following sociodemographic factors, that have demonstrated significant associations with alcohol use in a Norwegian employee population,[10] were included as controls in adjusted analyses: *Gender* (male; female), *age* (years), *educational attainment* (primary/lower secondary; upper secondary; university/college ≤4 years; university/college >4 years), *living status* (living alone; living with others), *marital status* (unmarried; married), and *number of children* (continuous measure).

Analysis

Descriptive analyses were performed on the main study variables (outcomes and predictors) and presented in terms of means, standard deviations, frequencies and percentages, as appropriate.

Main analyses were conducted in two stages: model selection and predictor comparison.[61] In the model selection stage, associations between workplace factors and alcohol consumption (AUDIT-C scale 0-12) were explored by means of multiple linear regression analyses. Crude and adjusted unstandardised coefficients (b) were calculated. Associations between workplace factors and the odds of alcohol-related problems (AUDIT-10 categories 0-7 vs. 8-40) were explored with multiple binary logistic regression analyses. Crude and adjusted odds ratios (OR) were calculated. In all adjusted analyses (linear and logistic), associations between predictors and outcomes were adjusted for gender, age, educational attainment, living status, marital status, and number of children. In order to avoid losing statistical power and to minimise issues relating to potential collinearity effects,

For peer review only

For peer review only - http://bmjopen.bmj.com/site/about/guidelines.xhtml

workplace factors were not adjusted for each other. Work schedule (categorical nominal predictor) was dummy coded.

In the predictor comparison stage, the relative importance of predictors that demonstrated significant associations with the outcomes (p <.05) in the model selection stage were estimated by means of dominance analysis (DA).[62] DA consists of examining R^2 values for all possible subset models in a multiple regression, with additional contribution of each predictor. A predictor is considered to be more important than its competitors if its predictive ability exceeds the others' in all subset regressions.[62, p. 545] Each predictor's relative importance was expressed in terms of its percentage of the full regression model's predictive ability. DA was performed separately for each outcome. $DA_{\text{alcohol consumption}}$ was based on R^2 values from multiple linear regression analyses, while $DA_{\text{alcohol-related problems}}$ were based on pseudo $R^2_{\text{Nagelkerke}}$ values from multiple binary logistic regression analyses.

All analyses were performed with IBM SPSS version 27, and significant results were defined as p <.05.

Patient and public involvement

Employee representatives and other relevant stakeholders were included in the WIRUS project's reference group. Design, research questions, recruitment procedures and choice of outcome measures in the project have been informed by priorities, experience and preferences expressed by the reference group.

RESULTS

Approximately one out of ten employees (11.3 %) reported alcohol-related problems (AUDIT ≥8). The sample's means score on alcohol consumption was 3.2 (on the AUDIT-C scale ranging from 0 to 12). Employees' mean rating of supervisors' desired leadership

For peer review only - http://bmjopen.bmj.com/site/about/guidelines.xhtml

qualities was 2.9 (on the 7LQQ scale ranging from 0 to 4). Job control and workplace social support were rated higher than psychological job demands (on the JCQ scales ranging from 1 to 4: $M_{\text{Job control}} = 3.0$; $M_{\text{Social support}} = 3.2$; $M_{\text{Psychological job demands}} = 2.2$). The majority of employees were workers (80.2 % of the total sample) with full-time (80.1 % of the total sample) day jobs without weekend work (73.2 % of the total sample). Descriptive statistics for the main study variables are presented in Table 3.

Table 3. Descriptive statistics for the main study variables

	Variable	
O	Alcohol consumption[A], *M* (*SD*)	3.2 (1.9)
O	Alcohol-related problems[B], n (%) yes	695 (11.3)
P	Working hours[C], *M* (*SD*)	7.9 (1.9)
P	Day job, no weekends, n (%) yes	4515 (73.2)
P	Day job, with weekends, n (%) yes	351 (5.7)
P	Evening job, n (%) yes	30 (0.5)
P	Night job, n (%) yes	96 (1.6)
P	Shift work, n (%) yes	1172 (19.0)
P	Working from home, n (%) yes	407 (6.6)
P	Working form holiday home, n (%) yes	177 (2.9)
P	Job travels, n (%) yes	852 (13.8)
P	Job position, n (%) workers	4944 (80.2)
P	Job size, n (%) full-time	4939 (80.1)
P	Leadership qualities[D], *M* (*SD*)	2.9 (0.8)
P	Psychological job demands[E], *M* (*SD*)	2.2 (0.4)
P	Job control[E], *M* (*SD*)	3.0 (0.3)
P	Workplace social support[E], *M* (*SD*)	3.2 (0.5)

N = 6164; O = outcome; P = predictor; *M* = mean; *SD* = standard deviation; [A]Alcohol Use Disorders Identification Test – Consumption (AUDIT-C), scale 0-12; [B]AUDIT (all items) (scale 0-40), sum score ≥8; [C]Number of hours during a typical day; [D]Seven-item Leadership Qualities Questionnaire (7LQQ), scale 0-4; [E]Job Content Questionnaire (JCQ), scale 1-4

Workplace factors and alcohol consumption

Unadjusted and adjusted associations between workplace factors and alcohol consumption are presented in Table 4.

For peer review only - http://bmjopen.bmj.com/site/about/guidelines.xhtml

Table 4. Associations between workplace factors and alcohol consumption[A]

	Crude		Adjusted[B]		
Predictor	b	*p*	b	95% CI for b	*p*
Working hours	**.03**	**.026**	.02	-.01, .04	.198
Day job, no weekends[C]	**-.17**	**.002**	.07	-.04, .17	.206
Day job, with weekends[C]	**.21**	**.038**	.11	-.09, .30	.288
Evening job[C]	.06	.871	.01	-.64, .65	.982
Night job[C]	-.33	.087	-.35	-.72, .01	.058
Shift work[C]	**.17**	**.006**	-.09	-.21, ,03	.142
Working from home[D]	.12	.202	.14	-.04, .32	.136
Working from holiday home[D]	**.53**	**<.001**	**.53**	**.26, .80**	**<.001**
Job travels[D]	**.36**	**<.001**	**.28**	**.15, .42**	**<.001**
Job position[E]	**.17**	**.006**	**.27**	**.15, .38**	**<.001**
Job size[F]	**.41**	**<.001**	**.28**	**.16, .39**	**<.001**
Leadership qualities[G]	-.06	.061	-.05	-.11, .00	.062
Psychological job demands[H]	-.07	.195	-.05	-.16, .05	.323
Job control[H]	-.00	.994	.14	-.00, .28	.051
Workplace social support[H]	.04	.441	.06	-.04, .16	.237

N = 6164; Results from multiple linear regression analyses; b = unstandardised regression coefficient; CI = confidence interval; [A]Alcohol Use Disorders Identification Test – Consumption (AUDIT-C), scale 0-12, higher scores indicate higher consumption; [B]Adjusted for gender, age, educational attainment, living status, marital status, having children (workplace factors not adjusted for each other); [C]Ref. = other work schedules; [D]Ref. = no; [E]Ref. = worker; [F]Ref. = part-time; [G]Seven-item Leadership Qualities Questionnaire (7LQQ), scale 0-4, higher scores indicate higher level of desired leadership qualities; [H]Job Content Questionnaire (JCQ), scale 1-4, higher scores indicate higher predictor values; Bold typeface indicates statistically significant associations (p <.05)

As shown in Table 4, adjusted analyses revealed that alcohol consumption was significantly associated with four workplace factors. Higher consumption was associated with working from holiday home, job travels, working full-time (as opposed to part-time), and being a supervisor (versus a worker). The remaining workplace factors either failed to demonstrate significant relationships with alcohol consumption altogether (evening job, night job, working from home, leadership qualities, psychological job demands, job control, workplace social support), or failed to reach statistical significance when covariates were taken into account (working hours, day job without weekends, day job with weekends, shift work). There were, however, borderline-significant tendencies indicating higher consumption to be associated with not having a night job (p = .058), having supervisors with lower levels of desired leadership qualities (p = .062), and lower levels of job control (p = .051).

For peer review only

For peer review only - http://bmjopen.bmj.com/site/about/guidelines.xhtml

The relative importance of the four significant workplace factors (working from holiday home, job travels, working full-time, being a supervisor) for explaining the variance in alcohol consumption is presented in the Supplementary File (Table S1). The four workplace factors (in a multiple model without sociodemographic covariates) collectively explained 1.2 % of the variance in alcohol consumption. Job size was the dominant predictor, accounting for 56.7 % of the variance in the model's proportion of explained variance of the outcome, followed by job travels (25.0 %), working from holiday home (12.5 %) and job position (4.2 %).

Workplace factors and alcohol-related problems

Unadjusted and adjusted associations between workplace factors and odds of having alcohol-related problems are presented in Table 5.

Table 5. Associations between workplace factors and odds of having alcohol-related problems[A]

	Crude		Adjusted[B]		
Predictor	OR	*p*	OR	95% CI for OR	*p*
Working hours	0.98	.289	0.97	0.93, 1.01	.139
Day job, no weekends[C]	**0.75**	**.001**	1.10	0.91, 1.32	.337
Day job, with weekends[C]	1.17	.346	1.01	0.72, 1.41	.957
Evening job[C]	1.58	.353	1.46	0.53, 4.02	.464
Night job[C]	1.13	.702	1.08	0.57, 2.03	.818
Shift work[C]	**1.33**	**.003**	0.87	0.71, 1.07	.872
Working from home[D]	0.98	.885	1.05	0.75, 1.47	.777
Working from holiday home[D]	1.06	.801	1.22	0.76, 1.98	.414
Job travels[D]	**1.26**	**.037**	1.23	0.98, 1.55	.080
Job position[E]	**0.77**	**.018**	0.98	0.78, 1.23	.977
Job size[F]	**1.43**	**.001**	1.24	0.98, 1.56	.072
Leadership qualities[G]	**0.88**	**.006**	**0.85**	**0.77, 0.94**	**.002**
Psychological job demands[H]	1.01	.945	1.02	0.85, 1.24	.822
Job control[H]	**0.72**	**.006**	0.88	0.69, 1.13	.307
Workplace social support[H]	**0.82**	**.022**	**0.81**	**0.68, 0.96**	**.016**

N = 6164; Results from multiple binary logistic regression analyses; OR = odds ratio; CI = confidence interval; [A]Alcohol Use Disorders Identification Test (AUDIT, all items), sum score 0-7 vs 8-40; [B]Adjusted for gender, age, educational attainment, living status, marital status, having children (workplace factors not adjusted for each other); [C]Ref. = other work schedules; [D]Ref. = no; [E]Ref. = worker; [F]Ref. = part-time; [G]Seven-item Leadership Qualities Questionnaire (7LQQ), scale 0-4, higher scores indicate higher level of desired

For peer review only - http://bmjopen.bmj.com/site/about/guidelines.xhtml

leadership qualities; [H]Job Content Questionnaire (JCQ), scale 1-4, higher scores indicate higher predictor values; Bold typeface indicates statistically significant associations (p <.05)

As shown in Table 5, only two workplace factors were significantly related with alcohol-related problems when adjusting for sociodemographic covariates. Elevated odds of alcohol-related problems was associated with lower scores on leadership qualities and workplace social support. The remaining workplace factors wither demonstrated a significant unadjusted association with alcohol-related problems but failed to reach statistical significance in adjusted analyses (day job without weekends, shift work, job travels, job position, job size, job control), or were unrelated with alcohol-related problems altogether (working hours, day job with weekends, evening job, night job, working from home, working from holiday home, psychological job demands).

Dominance analysis (Supplementary File, Table S2) revealed that leadership qualities and workplace social support contributed equally (50 %) in explaining alcohol-related problems. The model containing leadership qualities and workplace social support explained a total of only 0.3 % of the variance in alcohol-related problems.

Summary of main results

Main results are summarised in Figure 1.

[Figure 1 about here]

Based on results from adjusted analysis, 9 out of 15 workplace factors were not significantly related to either alcohol consumption or alcohol-related problems. Four workplace factors were significantly associated with alcohol consumption but not with alcohol-related problems (job size, job travels, working from holiday home, job position). Two workplace factors were significantly related to alcohol-related problems but not with

For peer review only - http://bmjopen.bmj.com/site/about/guidelines.xhtml

alcohol consumption (supervisor's leadership qualities, workplace social support). Overall, associations were quite weak (see Tables 4 and 5) and most of the variance in employees' alcohol consumption and alcohol-related problems was explained by variables not included in this study, i.e., by variables beyond the included workplace factors (see Supplementary File, Tables S1 and S2).

DISCUSSION

The aim of this study was to explore whether workplace factors were associated with alcohol use in a heterogeneous sample of employees in Norway.

Working full-time (as opposed to part-time) was associated with elevated alcohol consumption among employees. This finding contradicts earlier research from the USA that has found that part-time workers had higher weekly consumption than full-time workers.[41] Our data do not illuminate reasons for why full-time employees were more exposed to alcohol than part-time employees. One may hypothesise that this association can be interpreted in terms of the degree to which employees are socially integrated at the workplace. Evidence has demonstrated that a significant proportion of employees' overall alcohol consumption does indeed occur in job-related contexts. Studies of employees in Norway have indicated that 30 % had consumed alcohol in job-related settings during the past two weeks,[63] 43 % of employees' overall consumption occurred in job-related situations,[63] and employers initiated and organised more than half of the job-related situations in which employees were exposed to alcohol.[64] Part-time employees may have a lower workplace social integration than their full-time counterparts, perhaps resulting in a lower exposure to alcohol in job-related situations. Alternatively, part-time employees may have reduced working hours due to health problems,[65] which in turn may explain why they – on a group level – consume less alcohol than full-time employees. Employees' financial situation may as well be of

For peer review only

For peer review only - http://bmjopen.bmj.com/site/about/guidelines.xhtml

importance, insofar that alcohol is a costly commodity,[66] and that part-time workers may find themselves in a more strained financial situation than full-time employees.

Work location flexibility (e.g., working from home and having overall greater flexibility) and job travels have in some studies been linked with elevated alcohol consumption.[34, 39, 40] In our study, work location flexibility demonstrated mixed results. We did not find an association between alcohol consumption and working from home. However, working from holiday home and job travels were associated with elevated consumption. These findings may reflect a context-dependency in the relationship between work location flexibility and alcohol use. In an earlier study exploring relationships between activity patterns and alcohol use among employees in Norway,[67] non-domestic activities were associated with higher consumption than domestic activities. Having an activity pattern characterised by activities within (rather than outside) the household was associated with lower alcohol consumption. Working from home (in a domestic setting) may constitute working in an activity setting not very compatible with alcohol use, even though working from home entails being less visible for co-workers and supervisors and less susceptible to workplace social control (factors which have been linked with elevated alcohol consumption.[9, 22] Working from holiday home and job travels constitute non-domestic activities where work is performed in contexts more similar to vacations. Vacations and holidays typically involve greater alcohol exposure, potentially resulting in higher consumption levels.[68, 69]

Workers consumed less alcohol than supervisors, which is in line with some earlier studies.[31, 39] One may hypothesise that a supervisor position entails access and exposure to factors that may enhance alcohol consumption, such as higher salaries and greater work location flexibility (e.g., job travels and working from holiday home). However, our data do not lend themselves to explanations for the association between job position and alcohol

For peer review only - http://bmjopen.bmj.com/site/about/guidelines.xhtml

consumption, and several studies have failed to demonstrate such a relationship.[23, 30, 33, 34]

Having supervisors with desired leadership qualities was associated with reduced odds of having alcohol-related problems. Supervisors who are highly rated on desired leadership qualities (problem-solving, contact-making, responsible-making, protecting, trust-creating, recognising and encouraging;[57]) may be perceived as active, visible and involved with their staff and can be well-positioned to set norms and exert informal social control. It has been advocated that visible and active supervisors represent protective factors for elevated alcohol use among workers,[42] and studies have demonstrated lower prevalence of drinking and hangover episodes at work among employees employed at workplaces where supervisors exert active social control.[25, 45, 46] As such, supervisors with desired leadership qualities may be influential in determining workplace drinking cultures, i.e., the sociocultural prescriptions for appropriate levels of alcohol use, the purposes and settings for drinking, and how to behave in drinking situations.[70-72] Employees' level of drinking are likely to be influenced by the workplace drinking culture within which they are integrated.[26] One may assume that desired leadership qualities represent a potential protective factor for alcohol-related problems in line with how earlier studies have found such desired leadership to be a protective factor for mental distress,[73] and physiological pain.[74, 75]

In line with earlier research,[32, 37, 50] we found that higher levels of workplace social support (from co-workers and supervisors) were associated with reduced odds of having alcohol-related problems. A supportive workplace context may reflect a work climate that welcomes disclosure of potential alcohol-related problems, rendering it possible to provide early identification and intervention. Interestingly, leadership qualities and social support (which are to some degree overlapping due to supervisor support being an important aspect of workplace social support) were associated only with alcohol-related problems and not with

For peer review only - http://bmjopen.bmj.com/site/about/guidelines.xhtml

alcohol consumption. It may be that leadership and support in the work context have limited influence on employees' overall consumption level but may be of importance for understanding whether drinking translates into alcohol-related problems. Such an interpretation underscores the importance of the workplace as an arena for alcohol prevention interventions.

Methodological considerations

The cross-sectional design of this study precludes causal inferences regarding the relationships between workplace factors and alcohol-related outcomes. Even though we attempted to minimise risk of confounding by including relevant covariates in adjusted analyses, reversed, reciprocal and/or third-variable causation cannot be ruled out. For instance, the association between job size and alcohol use may be due to job size affecting drinking pattern, to drinking pattern affecting job size, or to an unmeasured third variable (e.g., health status) affecting both job size and drinking pattern. However, the aim of this study was not to unravel causal relationships but was explorative in nature.

The sample was large and consisted of a heterogeneous selection of employees across work sectors, divisions, levels and geographical locations. The sample size well exceeded the a priori defined criteria,[52, 53] and was thus considered statistically satisfactory (required sample size: N = 435; actual sample size: N = 6164). However, the response rate was quite low (27.7 %). Low response rates may represent a threat to external validity by limiting generalisability of results in instances where responders systematically differ from non-responders. Study selection analyses of the WIRUS screening data indicate that the final study sample was similar to the invited sample (i.e., all employees in the included companies) regarding distributions of gender and age.[76] However, compared with the entire Norwegian workforce, female, older and well-educated employees were overrepresented in our study.

For peer review only - http://bmjopen.bmj.com/site/about/guidelines.xhtml

There may also be important differences between the study sample and non-responders that we lack information about (unmeasured selection effects) that have biased our findings. Moreover, a considerable number of employees (n = 2378) were exluded due to not responding on all study items in the survey. Comparisons between completers and non-completers on sociodemographic variables (see Supplementary File, Table S3) demonstrated that completers were characterised by a slight overrepresentation of males, employees with university/college education, employees not living alone, married employees, and employees having more children.

This study was solely based on self-reported data from employees, which may involve risks of measuring bias (e.g., recall bias and social desirability bias). In particular, one may assume that alcohol use may have been underestimated, insofar that studies have demonstrated a discrepancy between self-reported consumption and actual alcohol sales.[77] On the other hand, alcohol consumption and alcohol-related problems were both measured with the AUDIT, which has demonstrated psychometric properties superior to other alcohol screening instruments,[78] and has been deemed appropriate for use in Norwegian employee populations.[79] A threshold of eight points on the AUDIT as indicative of alcohol-related problems has been found to represent a satisfactory compromise between sensitivity and specificity,[8] and are in line with previous research on non-clinical populations.[80-83]

Implications

Although not being able to generate a clear profile of workplace factors related to employee alcohol use, results from this study imply the importance of supervisors' leadership qualities and workplace social support. Higher levels of workplace social support and having managers with desired leadership qualities emerged as potential protective factors for alcohol-related problems among employees. As such, results indicate that efforts aimed at reducing

For peer review only - http://bmjopen.bmj.com/site/about/guidelines.xhtml

harmful alcohol use in working populations should target leadership styles and the workplace context (e.g., social support and drinking culture), as well as individual employees. Efforts may include fostering desired leadership qualities among managers, raise managers' awareness of their influence on workplace drinking culture, and ensuring workers serviceable levels of social support from co-workers and managers. Prosocial supervisors may stimulate workplace cultures that discourage or inhibit drinking in work-related situations and may also foster social relationships and trust that enable establishment of routines for early identification and aid for employees who may benefit from alcohol prevention interventions. In a systematic review,[84] collaboration/teamwork and positive, accessible and fair supervisors were identified among the most pronounced factors considered as important for a healthy workplace. In another systematic review,[85] covering workplace resources to improve employee well-being and performance, social support and high-quality relationships between workers and supervisors were identified among the factors organisations should emphasise. As such, a focus on workplace factors identified in our study as potential protective factors for alcohol-related problems seem to converge with the WHO framework for healthy workplaces, i.e., workplaces "in which workers and managers collaborate to use a continual improvement process to protect and promote health, safety and well-being of all workers and the sustainability of the workplace by considering" important aspects such as the psychosocial work environment, the organisation of work, and the workplace culture.[86, p. 16]

Evidence on associations between workplace factors and employee alcohol use still stands out as mixed and inconclusive, and this study showed that only marginal proportions of the variance in alcohol consumption and alcohol-related problems were explained by the included workplace factors. Hence, further research is warranted. Results from this study suggest the application and further development of interventions, e.g., Workplace Health Promotion

For peer review only - http://bmjopen.bmj.com/site/about/guidelines.xhtml

Programs (WHPP), targeting workplace leadership, drinking culture, and social support. Effects and implementation of such interventions should be explored by means of robust research designs, such as prospective cohort studies and cluster randomised controlled trials. Future research may also benefit from explorations of potential interactions between individual and workplace factors, including mediators and moderators, which were beyond the scope of our study.

Conclusions

Knowledge about workplace factors associated with employee alcohol use is important as an aid in determining the extent to which employers should make workplace-based alcohol prevention interventions an overall priority. This study, conducted in a large and heterogeneous sample of employees in Norway, was unable to generate a clear profile of workplace factors associated with alcohol use, but points to the importance of leadership qualities and workplace social support as potential protective factors for alcohol-related problems.

DECLARATIONS

Ethics approval statement

Companies and employees were provided with information about the study's aim, they were assured confidentiality, that participation was voluntary, and that they could withdraw from the study at any given time. Written informed consent was obtained from all included employees. The study was approved by the Regional Committee for Medical and Health Research in Norway (REK; approval no. 2014/647).

For peer review only

For peer review only - http://bmjopen.bmj.com/site/about/guidelines.xhtml

Author contributions

RWA is the principal investigator (PI) and project manager (PM) for the WIRUS project (Workplace Interventions preventing Risky alcohol Use and Sick leave). This study was designed by MMT and RWA. MMT analysed the data and drafted the manuscript. JCS, TB, LSS, RWA and MMT provided scientific input to the different drafts and provided data interpretation. All authors made critical revisions and provided intellectual content to the manuscript, approved the final version to be published, and agreed to be accountable for all aspects of this work.

Funding

This work was supported by the Norwegian Directorate of Health (grant number: n/a) and the Research Council of Norway (grant number: 260640).

Competing interests

None declared.

Data availability statement

De-identified data from the WIRUS Screening study are available from the corresponding author on reasonable request.

SUPPLEMENTARY FILE

Supplementary File 1: Dominance analyses (PDF)

Supplementary File 2: Study selection analyses (PDF)

REFERENCES

For peer review only - http://bmjopen.bmj.com/site/about/guidelines.xhtml

1 Griswold MG, Fullman N, Hawley C, et al. Alcohol use and burden for 195 countries and territories, 1990–2016: a systematic analysis for the Global Burden of Disease Study 2016. *Lancet* 2018;392:1015-35. doi: 10.1016/s0140-6736(18)31310-2

2 Rehm J, Gmel GE, Gmel G, et al. The relationship between different dimensions of alcohol use and the burden of disease - an update. *Addiction* 2017;112:968-1001. doi: 10.1111/add.13757

3 Room R, Babor T, Rehm J. Alcohol and public health. *Lancet* 2005;365:519-30. doi: 10.1016/s0140-6736(05)70276-2

4 Wood AM, Kaptoge S, Butterworth AS, et al. Risk thresholds for alcohol consumption: combined analysis of individual-participant data for 599 912 current drinkers in 83 prospective studies. *Lancet* 2018;391:1513-23. doi: 10.1016/S0140-6736(18)30134-X

5 World Health Organization. Global status report on alcohol and health 2018. Geneva, Switzerland: World Health Organization 2018.

6 Vos T, Lim SS, Abbafati C, et al. Global burden of 369 diseases and injuries in 204 countries and territories, 1990–2019: a systematic analysis for the Global Burden of Disease Study 2019. *Lancet* 2020;396:1204-22.

7 Collin J, Casswell S. Alcohol and the sustainable development goals. *Lancet* 2016;387:2582-83. doi: 10.1016/S0140-6736(16)30827-3

8 Babor TF, Higgins-Biddle JC, Saunders JB, et al. AUDIT: The alcohol use disorders identification test. Guidelines for use in primary health care. 2nd ed. Geneva, Switzerland: World Health Organization 2001.

9 Frone MR. Alcohol and illicit drug use in the workforce and workplace. Washington, DC: American Psychological Association 2013.

10 Thørrisen MM, Skogen JC, Aas RW. The associations between employees' risky drinking and sociodemographics, and implications for intervention needs. *BMC Public Health* 2018;18 doi: 10.1186/s12889-018-5660-x

11 Amiri S, Behnezhad S. Alcohol consumption and sick leave: a meta-analysis. *J Addict Dis* 2020;38:100-12. doi: 10.1080/10550887.2020.1724606

12 Hashemi N, Skogen JC, Sevic A, et al. A systematic review and meta-analysis uncovering the relationship between alcohol consumption and sickness absence. When type of design, data, and sickness absence make a difference. *PLoS ONE* 2022;17 doi: 10.1371/journal.pone.0262458

13 Marzan M, Callinan S, Livingston M, et al. Systematic review and dose-response meta-analysis on the relationship between alcohol consumption and sickness absence. *Alcohol Alcohol* 2021 doi: 10.1093/alcalc/agab008

14 Schou L, Moan IS. Alcohol use–sickness absence association and the moderating role of gender and socioeconomic status: A literature review. *Drug Alcohol Rev* 2016;35:158-69. doi: 10.1111/dar.12278

15. Ford MT, Cerasoli CP, Higgins JA, et al. Relationships between psychological, physical, and behavioural health and work performance: A review and meta-analysis. *Work & Stress* 2011;25:185-204. doi: 10.1080/02678373.2011.609035

16 Thørrisen MM, Bonsaksen T, Hashemi N, et al. Association between alcohol consumption and impaired work performance (presenteeism): a systematic review. *BMJ Open* 2019;9 doi: 10.1136/bmjopen-2019-029184

For peer review only - http://bmjopen.bmj.com/site/about/guidelines.xhtml

17 Kaner EF, Beyer FR, Muirhead C, et al. Effectiveness of brief alcohol interventions in primary care populations. *Cochrane Database Syst Rev* 2018;2:CD004148. doi: 10.1002/14651858.CD004148.pub4

18 O'Donnell P, Anderson P, Newbury-Birch D, et al. The impact of brief alcohol interventions in primary healthcare: A systematic review of reviews. *Alcohol Alcohol* 2014;49:66-78. doi: 10.1093/alcalc/agt170

19 Schulte B, O'Donnell AJ, Kastner S, et al. Alcohol screening and brief intervention in workplace settings and social services: A comparison of literature. *Front Psychiatry* 2014;5 doi: 10.3389/fpsyt.2014.00131

20 Webb G, Shakeshaft A, Sanson-Fisher R, et al. A systematic review of work-place interventions for alcohol-related problems. *Addiction* 2009;104:365-77. doi: 10.1111/j.1360-0443.2008.02472.x

21 Yuvaraj K, Eliyas SK, Gokul S, et al. Effectiveness of workplace intervention for reducing alcohol consumption: a systematic review and meta-analysis. *Alcohol Alcohol* 2019;54:264-71. doi: 10.1093/alcalc/agz024

22 Frone MR. Predictors of overall and on-the-job substance use among young workers. *J Occup Health Psychol* 2003;8:39-54. doi: 10.1037/1076-8998.8.1.39

23 Marchand A, Parent-Lamarche A, Blanc ME. Work and high-risk alcohol consumption in the Canadian workforce. *Int J Environ Res Public Health* 2011;8:2692-705. doi: 10.3390/ijerph8072692

24 Skogen JC, Bøe T, Thørrisen MM, et al. Sociodemographic characteristics associated with alcohol consumption and alcohol-related consequences, a latent class analysis of the Norwegian WIRUS screening study. *BMC Public Health* 2019;19 doi: 10.1186/s12889-019-7648-6

25 Ames GM, Grube JW, Moore RS. Social control and workplace drinking norms: A comparison of two organizational cultures. *J Stud Alcohol* 2000;61:203-19. doi: 10.15288/jsa.2000.61.203

26 Barrientos-Gutierrez T, Gimeno D, Mangione TW, et al. Drinking social norms and drinking behaviours: a multilevel analysis of 137 workgroups in 16 worksites. *Occup Environ Med* 2007;64:602-08. doi: 10.1136/oem.2006.031765

27 Hodgins DC, Williams R, Munro G. Workplace responsibility, stress, alcohol availability and norms as predictors of alcohol consumption-related problems among employed workers. *Subst Use Misuse* 2009;44:2062-69. doi: 10.3109/10826080902855173

28 Pachito DV, Pega F, Bakusic J, et al. The effect of exposure to long working hours on alcohol consumption, risky drinking and alcohol use disorder: A systematic review and meta-analysis from the WHO/ILO Joint Estimates of the Work-related Burden of Disease and Injury. *Environ Int* 2021;146 doi: 10.1016/j.envint.2020.106205

29 Virtanen M, Jokela M, Nyberg ST, et al. Long working hours and alcohol use: systematic review and meta-analysis of published studies and unpublished individual participant data. *BMJ* 2015;350 doi: 10.1136/bmj.g7772

30 Frone MR. Prevalence and distribution of alcohol use and impairment in the workplace: a U.S. national survey. *J Stud Alcohol* 2006;67:147-56. doi: 10.15288/jsa.2006.67.147

31 Marchand A. Alcohol use and misuse: What are the contributions of occupation and work organization conditions? *BMC Public Health* 2008;8 doi: 10.1186/1471-2458-8-333

For peer review only

For peer review only - http://bmjopen.bmj.com/site/about/guidelines.xhtml

32 Saade SL, Marchand A. Work organization conditions, alcohol misuse: The moderating role of personality traits. *Work* 2013;44:191-200. doi: 10.3233/WOR-2012-1408

33 Cheng WJ, Cheng Y, Huang MC, et al. Alcohol dependence, consumption of alcoholic energy drinks and associated work characteristics in the Taiwan working population. *Alcohol Alcohol* 2012;47:372-79. doi: 10.1093/alcalc/ags034

34 Moan IS, Halkjelsvik T. Alkohol og arbeidsliv. En undersøkelse blant norske arbeidstakere [Alcohol and work. A survey among Norwegian employees]. Oslo, Norway: Norwegian Institute of Public Health 2016.

35 Plescia F, Cirrincione L, Martorana D, et al. Alcohol abuse and insomnia disorder: Focus on a group of night and day workers. *Int J Environ Res Public Health* 2021;18 doi: 10.3390/ijerph182413196

36 Buchvold HV, Pallesen S, Øyane NMF, et al. Associations between night work and BMI, alcohol, smoking, caffeine and exercise - a cross-sectional study. *BMC Public Health* 2015;15 doi: 10.1186/s12889-015-2470-2

37 Ansoleaga E, Montano R, Vezina M. Psychosocial risk at work and hazardous alcohol consumption among Chile's working adults. *Can J Public Health* 2013;104:502-08. doi: 10.17269/cjph.104.4045

38 Hermansson U, Knutsson A, Brandt L, et al. Screening for high-risk and elevated alcohol consumption in day and shift workers by use of the AUDIT and CDT. *Occup Med* 2003;53 doi: 10.1093/occmed/kqg104

39 Nielsen MB, Christensen JO, Knardahl S. Working at home and alcohol use. *Addict Behav Rep* 2021;14 doi: 10.1016/j.abrep.2021.100377

40 Macdonald S, Wells S, Wild TC. Occupational risk factors associated with alcohol and drug problems. *Am J Drug Alcohol Abuse* 1999;25:351-69. doi: 10.1081/ADA-100101865

41 Lee CM, Cadigan JM, Fairlie AM, et al. Transitions into young adulthood: Extent to which alcohol use, perceived drinking norms, and consequences vary by education and work statuses among 18-20 year olds. *Addict Behav* 2018;79:107-12. doi: 10.1016/j.addbeh.2017.12.004

42 Roman PM, Trice HM. The development of deviant drinking behavior. *Arch Environ Health* 1970;20:424-35. doi: 10.1080/00039896.1970.10665615

43 Bacharach SB, Bamberger PA, Sonnenstuhl WJ. Driven to drink: Managerial control, work-related risk factors, and employee problem drinking. *Acad Manage J* 2002;45:637-58. doi: 10.5465/3069302

44 Nielsen MB, Gjerstad J, Frone MR. Alcohol use and psychosocial stressors in the Norwegian workforce. *Subst Use Misuse* 2018;53:574-84. doi: 10.1080/10826084.2017.1349797

45 Frone MR, Trinidad JR. Testing a general model of employee alcohol use and workplace productivity among U.S. workers. *Alcohol Clin Exp Res* 2012;36:229A.

46 Moore RS, Ames GM, Duke MR, et al. Food service employee alcohol use, hangovers and norms during and after work hours. *Journal of Substance Use* 2012;17 doi: 10.3109/14659891.2011.580414

47 Heikkilä K, Fransson EI, Nyberg ST, et al. Job strain and health-related lifestyle: findings from an individual-participant meta-analysis of 118 000 working adults. *Am J Public Health* 2013;103:2090-97.

For peer review only - http://bmjopen.bmj.com/site/about/guidelines.xhtml

48 Roxburgh S. Gender differences in the effect of job stressors on alcohol consumption. *Addict Behav* 1998;23:101-07. doi: 10.1016/S0306-4603(97)00025-7

49 Legleye S, Baumann M, Peretti-Watel P, et al. Gender and age disparities in the associations of occupational factors with alcohol abuse and smoking in the French working population. *Revue d'Epidemiologie et de Sante Publique* 2011;59:223-32. doi: 10.1016/j.respe.2011.02.103

50 Dobson KG, Ibrahim S, Gilbert-Ouimet M, et al. Association between psychosocial work conditions and latent alcohol consumption trajectories among men and women over a 16-year period in a national Canadian sample. *J Epidemiol Community Health* 2018;72:113-20. doi: 10.1136/jech-2017-209691

51 Skogen JC, Thørrisen MM, Bonsaksen T, et al. Effort-reward imbalance is associated with alcohol-related problems. WIRUS-screening study. *Front Psychol* 2019;10 doi: 10.3389/fpsyg.2019.02079

52 Tabachnick BG, Fidell LS. Using multivariate statistics. 6th ed. Boston, MA: Pearson Education 2013.

53 Stevens J. Applied multivariate statistics for the social sciences. 3rd ed. Mahwah, NJ: Lawrence Erlbaum 1996.

54 Saunders JB, Aasland OG, Babor TF, et al. Development of the Alcohol Use Disorders Identification Test (AUDIT): WHO collaborative project on early detection of persons with harmful alcohol consumption-II. *Addiction* 1993;88:791-804. doi: 10.1111/j.1360-0443.1993.tb02093.x

55 Bush K, Kivlahan DR, McDonell MB, et al. The AUDIT alcohol consumption questions (AUDIT-C). An effective brief screening test for problem drinking. *Arch Intern Med* 1998;158:1789-95. doi: 10.1001/archinte.158.16.1789

56 Aas RW. Workplace based sick leave prevention and return to work. Exploratory studies (doctoral thesis). Stockholm, Sweden: Karolinska Institute (Department of Clinical Neuroscience) 2011.

57 Aas RW, Ellingsen KL, Lindøe P, et al. Leadership qualities in the return to work process: a content analysis. *J Occup Rehabil* 2008;18 doi: 10.1007/s10926-008-9147-y

58 Karasek R, Brisson C, Kawakami N, et al. The Job Content Questionnaire (JCQ): An instrument for internationally comparative assessments of psychosocial job characteristics. *J Occup Health Psychol* 1998;3:322-55. doi: 10.1037/1076-8998.3.4.322

59 Johnson JV, Hall EM, Theorell T. Combined effects of job strain and social isolation on cardiovascular disease morbidity and mortality in a random sample of the Swedish male working population. *Scand J Work Environ Health* 1989;15:271-79. doi: 10.5271/sjweh.1852

60 Karasek R. Job demands, job decision latitude, and mental strain: implications for job redesign. *Adm Sci Q* 1979;24:285. doi: 10.2307/2392498

61 Azen R, Budescu DV. The dominance analysis approach for comparing predictors in multiple regression. *Psychol Methods* 2003;8:129-48. doi: 10.1037/1082-989x.8.2.129

62 Budescu DV. Dominance analysis: A new approach to the problem of relative importance of predictors in multiple regression. *Psychol Bull* 1993;114:542-51. doi: 10.1037/0033-2909.114.3.542

63 Nesvåg S, Lie T. Rusmiddelbruk blant ansatte i norsk privat arbeidsliv [Drug use among employees in Norwegian private sector]. *Nord Alkohol Nark* 2004;21:91-109.

For peer review only - http://bmjopen.bmj.com/site/about/guidelines.xhtml

64 Nordaune K, Skarpaas LS, Sagvaag H, et al. Who initiates and organises situations for work-related alcohol use? The WIRUS culture study. *Scand J Public Health* 2017;45:749-56. doi: 10.1177/1403494817704109

65 Nicolaisen H, Bråthen K. Frivillig deltid - en privatsak? [Voluntary part-time work - a private matter?]. Oslo, Norway: Fafo 2012.

66 Kan MY, Lan M. Comparing alcohol affordability in 65 cities worldwide. *Drug Alcohol Rev* 2013;32:19-26. doi: 10.1111/j.1465-3362.2012.00476.x

67 Moe MSH, Aas RW, Thørrisen MM. Tid brukt på daglige aktiviteter og alkoholbruk - WIRUS screening studien [Time spent on daily activities and alcohol consumption. The WIRUS screening study]. *Ergoterapeuten* 2020;63:76-87.

68 Carr N. Going with the flow: An assessment of the relationship between young people's leisure and holiday behaviour. *Tourism Geographies* 2002;4:115-34. doi: 10.1080/14616680210124891

69 Heacock RM, Capodilupo ER, Czeisler ME, et al. Sleep and alcohol use patterns during federal holidays in the United States. *MedRxiv* 2021 doi: 10.1101/2021.11.04.21264353

70 Ames GM, Janes C. A cultural approach to conceptualizing alcohol and the workplace. *Alcohol Health Res World* 1992;16:112-19.

71 Gordon R, Heim D, MacAskill S. Rethinking drinking cultures: A review of drinking cultures and a reconstructed dimensional approach. *Public Health* 2012;126:3-11. doi: 10.1016/j.puhe.2011.09.014

72 Savic M, Room R, Mugavin J, et al. Defining "drinking culture": A critical review of its meaning and connotation in social research on alcohol problems. *Drugs* 2016;23:270-82. doi: 10.3109/09687637.2016.1153602

73 Finne LB, Christensen JO, Knardahl S. Psychological and social work factors as predictors of mental distress: a prospective study. *PLoS ONE* 2014;9 doi: 10.1371/journal.pone.0102514

74 Sterud T, Johannessen HA, Tynes T. Work-related psychosocial and mechanical risk factors for neck/shoulder pain: a 3-year follow-up study of the general working population in Norway. *Int Arch Occup Environ Health* 2014;87:471-81. doi: 10.1007/s00420-013-0886-5

75 Christensen JO, Nielsen MB, Finne LB, et al. Comprehensive profiles of psychological and social work factors as predictors of site-specific and multi-site pain. *Scand J Work Environ Health* 2018;44:291-302. doi: 10.5271/sjweh.3706

76 Thørrisen MM. Alcohol consumption and impaired work performance. Interventions, and implementation barriers (doctoral thesis). Oslo, Norway: OsloMet - Oslo Metropolitan University (Faculty of Health Sciences) 2020.

77 Boniface S, Kneale J, Shelton N. Drinking pattern is more strongly associated with under-reporting of alcohol consumption than socio-demographic factors: evidence from a mixed-methods study. *BMC Public Health* 2014;14 doi: 10.1186/1471-2458-14-1297

78 de Meneses-Gaya C, Zuardi AW, Loureiro SR, et al. Alcohol Use Disorders Identification Test (AUDIT): An updated systematic review of psychometric properties. *Psychol Neurosci* 2009;2:83-97. doi: 10.3922/j.psns.2009.1.12

79 Skogen JC, Thørrisen MM, Olsen E, et al. Evidence for essential unidimensionality of AUDIT and measurement invariance across gender, age and education. Results from the WIRUS study. *Drug Alcohol Depend* 2019;202:87-92. doi: 10.1016/j.drugalcdep.2019.06.002

For peer review only - http://bmjopen.bmj.com/site/about/guidelines.xhtml

80 Aalto M, Tuunanen M, Sillanaukee P, et al. Effectiveness of structured questionnaires for screening heavy drinking in middle-aged women. *Alcohol Clin Exp Res* 2006;30:1884-88. doi: 10.1111/j.1530-0277.2006.00233.x

81 Giang KB, Spak F, Dzung TV, et al. The use of AUDIT to assess level of alcohol problems in rural Vietnam. *Alcohol Alcohol* 2005;40:578-83. doi: 10.1093/alcalc/agh198

82 Selin KH. Test-retest reliability of the Alcohol Use Disorder Identification Test in a general population sample. *Alcohol Clin Exp Res* 2003;27:1428-35. doi: 10.1097/01.alc.0000085633.23230.4a

83 Tuunanen M, Aalto M, Seppä K. Binge drinking and its detection among middle-aged men using AUDIT, AUDIT-C and AUDIT-3. *Drug Alcohol Rev* 2007;26:295-99. doi: 10.1080/09595230701247756

84 Lindberg P, Vingård E. Indicators of healthy work environments - a systematic review. *Work* 2012;41:3032-38. doi: 10.3233/wor-2012-0560-3032

85 Nielsen K, Nielsen MB, Ogbonnaya C, et al. Workplace resources to improve both employee well-being and performance: a systematic review and meta-analysis. *Work Stress* 2017;31:101-20. doi: 10.1080/02678373.2017.1304463

86 Burton J. WHO healthy workplace framework and model: background and supporting literature and practice. Geneva, Switzerland: World Health Organization 2010.

FIGURE TITLE/LEGEND

Figure 1. Overview of main study results. Arrow sizes and percentages reflect relative importance of predictors in explaining outcome (based on dominance analysis)

For peer review only

For peer review only - http://bmjopen.bmj.com/site/about/guidelines.xhtml

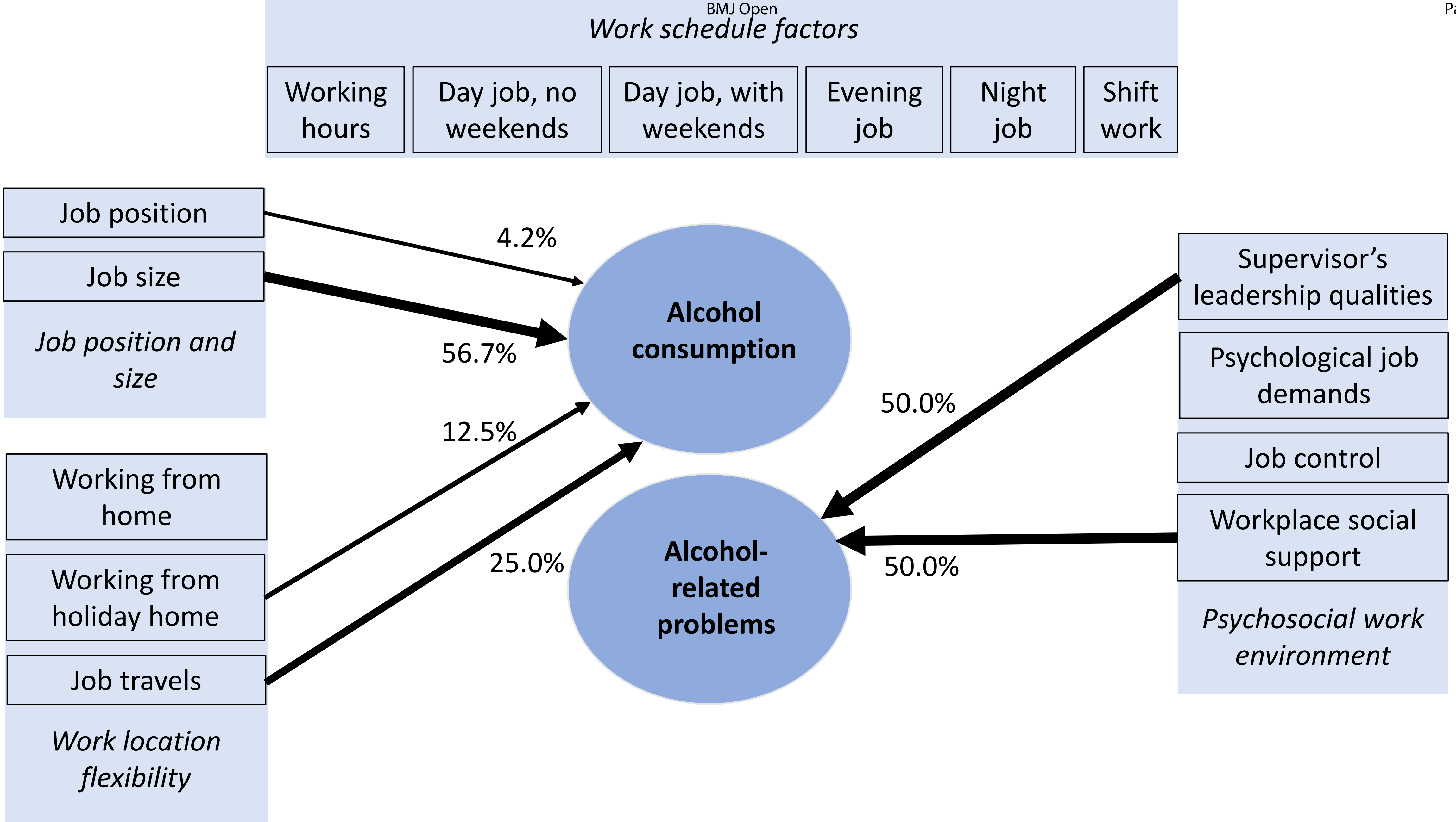
Work schedule factors
Working hours
Day job, no weekends
Day job, with weekends
Evening job
Night job
Shift work
Job position
Job size
Job position and size
4.2%
56.7%
12.5%
25.0%
Working from home
Working from holiday home
Job travels
Work location flexibility
Alcohol consumption
Alcohol-related problems
50.0%
50.0%
Supervisor's leadership qualities
Psychological job demands
Job control
Workplace social support
Psychosocial work environment

Supplementary File 1. Dominance analyses

Table S1. Relative importance of workplace factors[A] in explaining alcohol consumption[B]

		Additional contribution of			
Subset model (X)	$R^2_{y \cdot x}$	X_1 (Working from holiday home)	X_2 (Job travels)	X_3 (Job position)	X_4 (Job size)
Null and *k* = 0 average	0	.002	.004	.001	.008
X_1	.002	-	.003	.001	.007
X_2	.004	.001	-	.001	.006
X_3	.001	.002	.004	-	.007
X_4	.008	.002	.003	.000	-
k = 1 average		.002	.003	.001	.007
X_1X_2	.006	-	-	.001	.006
X_1X_3	.003	-	.003	-	.006
X_1X_4	.009	-	.002	.000	-
X_2X_3	.005	.001	-	-	.006
X_2X_4	.011	.001	-	.000	-
X_3X_4	.008	.002	.003	-	-
k = 2 average		.001	.003	.000	.006
$X_1X_2X_3$	.006	-	-	-	.006
$X_1X_2X_4$	.012	-	-	.000	-
$X_1X_3X_4$	.010	-	.002	-	-
$X_2X_3X_4$	.011	.001	-	-	-
k = 3 average		.001	.002	.000	.006
$X_1X_2X_3X_4$	.012				
**Overall average**		**.0015**	**.0030**	**.0005**	**.0068**
**% relative importance**		**12.5 %**	**25.0 %**	**4.2 %**	**56.7 %**

Results from dominance analysis; N = 6164; [A]Workplace factors that demonstrated significant adjusted associations with alcohol consumption; [B]Alcohol Use Disorders Identification Test – Consumption (AUDIT-C), scale 0-12; X_1 = Working from holiday home; X_2 = Job travels; X_3 = Job position; X_4 = Job size; Values in table indicate proportion of explained variance (R^2)

For peer review only

Table S2. Relative importance of workplace factors[A] in explaining alcohol-related problems[B]

Subset model (X)	$R^2_{y \cdot x}$	Additional contribution of X1 (Leadership qualitites)	Additional contribution of X2 (Social support)
Null and *k* = 0 average	0	.002	.002
X_1	.002	-	.001
X_2	.002	.001	-
k = 1 average		.001	.001
X_1X_2	.003		
**Overall average**		**.00015**	**.00015**
**Percentage relative importance**		**50.00 %**	**50.00 %**

Results from dominance analysis; N = 6164; [A]Workplace factors that demonstrated significant adjusted associations with alcohol-related problems; [B]Alcohol Use Disorders Identification Test (AUDIT, all items), sum score 0-7 vs 8-40; X_1 = Leadership qualities; X_2 = Workplace social support; Values in table indicate proportion of explained variance ($R^2_{Nagelkerke}$)

For peer review only

Supplementary File 2. Study selection analyses

Table S3. Comparisons between completers and non-completers on sociodemographic variables

Variable	Completers[A]	Non-completers[B]	*p*
Gender, % females	70.4	72.9	.023
Age, *M*	44.8	44.5	.723
Education, % university/college	77.9	66.5	<.001
Livings status, % living alone	14.7	16.3	.069
Marital status, % unmarried	44.4	50.1	<.001
Children, *M*	1.8	1.7	<.001

[A]Employees who responded on all study items, i.e., the study sample (n = X); [B]Employees who responded on the sociodemographic items but not on all study items (n = X); [C]Difference tested with chi square tests of independence; [D]Difference tested with independent samples t-tests

For peer review only

STROBE 2007 (v4) Statement—Checklist of items that should be included in reports of *cross-sectional studies*

Section/Topic	Item #	Recommendation	Reported on page #
**Title and abstract**	1	(*a*) Indicate the study's design with a commonly used term in the title or the abstract	1,2
		(*b*) Provide in the abstract an informative and balanced summary of what was done and what was found	2
**Introduction**			
Background/rationale	2	Explain the scientific background and rationale for the investigation being reported	3-7
Objectives	3	State specific objectives, including any prespecified hypotheses	7
**Methods**			
Study design	4	Present key elements of study design early in the paper	7
Setting	5	Describe the setting, locations, and relevant dates, including periods of recruitment, exposure, follow-up, and data collection	7-8
Participants	6	(*a*) Give the eligibility criteria, and the sources and methods of selection of participants	7-8
Variables	7	Clearly define all outcomes, exposures, predictors, potential confounders, and effect modifiers. Give diagnostic criteria, if applicable	9-11
Data sources/ measurement	8*	For each variable of interest, give sources of data and details of methods of assessment (measurement). Describe comparability of assessment methods if there is more than one group	9-11
Bias	9	Describe any efforts to address potential sources of bias	11-12
Study size	10	Explain how the study size was arrived at	8-9
Quantitative variables	11	Explain how quantitative variables were handled in the analyses. If applicable, describe which groupings were chosen and why	11-12
Statistical methods	12	(*a*) Describe all statistical methods, including those used to control for confounding	11-12
		(*b*) Describe any methods used to examine subgroups and interactions	n/a
		(*c*) Explain how missing data were addressed	8
		(*d*) If applicable, describe analytical methods taking account of sampling strategy	n/a
		(*e*) Describe any sensitivity analyses	n/a
**Results**			

For peer review only

Participants	13*	(a) Report numbers of individuals at each stage of study—eg numbers potentially eligible, examined for eligibility, confirmed eligible, included in the study, completing follow-up, and analysed	7-8
		(b) Give reasons for non-participation at each stage	n/a
		(c) Consider use of a flow diagram	n/a
Descriptive data	14*	(a) Give characteristics of study participants (eg demographic, clinical, social) and information on exposures and potential confounders	8
		(b) Indicate number of participants with missing data for each variable of interest	8
Outcome data	15*	Report numbers of outcome events or summary measures	9
Main results	16	(*a*) Give unadjusted estimates and, if applicable, confounder-adjusted estimates and their precision (eg, 95% confidence interval). Make clear which confounders were adjusted for and why they were included	11, 12-17, Suppl file
		(*b*) Report category boundaries when continuous variables were categorized	9-11
		(*c*) If relevant, consider translating estimates of relative risk into absolute risk for a meaningful time period	n/a
Other analyses	17	Report other analyses done—eg analyses of subgroups and interactions, and sensitivity analyses	n/a
**Discussion**			
Key results	18	Summarise key results with reference to study objectives	17-20
Limitations	19	Discuss limitations of the study, taking into account sources of potential bias or imprecision. Discuss both direction and magnitude of any potential bias	20, 21
Interpretation	20	Give a cautious overall interpretation of results considering objectives, limitations, multiplicity of analyses, results from similar studies, and other relevant evidence	17-20, 21-23
Generalisability	21	Discuss the generalisability (external validity) of the study results	20, 21
**Other information**			
Funding	22	Give the source of funding and the role of the funders for the present study and, if applicable, for the original study on which the present article is based	23

*Give information separately for cases and controls in case-control studies and, if applicable, for exposed and unexposed groups in cohort and cross-sectional studies.

Note: An Explanation and Elaboration article discusses each checklist item and gives methodological background and published examples of transparent reporting. The STROBE checklist is best used in conjunction with this article (freely available on the Web sites of PLoS Medicine at http://www.plosmedicine.org/, Annals of Internal Medicine at http://www.annals.org/, and Epidemiology at http://www.epidem.com/). Information on the STROBE Initiative is available at www.strobe-statement.org.

For peer review only

BMJ Open

Are workplace factors associated with employee alcohol use? The WIRUS cross-sectional study

Journal:	*BMJ Open*
Manuscript ID	bmjopen-2022-064352.R1
Article Type:	Original research
Date Submitted by the Author:	09-Aug-2022
Complete List of Authors:	Thørrisen, Mikkel; Oslo Metropolitan University, Department of Occupational Therapy, Prosthetics and Orthotics; University of Stavanger, Department of Public Health<br>Skogen, Jens; Nasjonalt folkehelseinstitutt, Department of Health Promotion; Stavanger University Hospital<br>Bonsaksen, Tore; Inland Norway University of Applied Sciences; VID Specialized University - Sandness<br>Skarpaas, Lisebet; Oslo Metropolitan University, Department of Occupational Therapy, Prosthetics and Orthotics<br>Aas, Randi; Oslo Metropolitan University, Department of Occupational Therapy, Prosthetics and Orthotics; University of Stavanger, Department of Public Health
**Primary Subject Heading**:	Public health
Secondary Subject Heading:	Occupational and environmental medicine
Keywords:	OCCUPATIONAL & INDUSTRIAL MEDICINE, PUBLIC HEALTH, EPIDEMIOLOGY

SCHOLARONE™
Manuscripts

I, the Submitting Author has the right to grant and does grant on behalf of all authors of the Work (as defined in the below author licence), an exclusive licence and/or a non-exclusive licence for contributions from authors who are: i) UK Crown employees; ii) where BMJ has agreed a CC-BY licence shall apply, and/or iii) in accordance with the terms applicable for US Federal Government officers or employees acting as part of their official duties; on a worldwide, perpetual, irrevocable, royalty-free basis to BMJ Publishing Group Ltd ("BMJ") its licensees and where the relevant Journal is co-owned by BMJ to the co-owners of the Journal, to publish the Work in this journal and any other BMJ products and to exploit all rights, as set out in our licence.

The Submitting Author accepts and understands that any supply made under these terms is made by BMJ to the Submitting Author unless you are acting as an employee on behalf of your employer or a postgraduate student of an affiliated institution which is paying any applicable article publishing charge ("APC") for Open Access articles. Where the Submitting Author wishes to make the Work available on an Open Access basis (and intends to pay the relevant APC), the terms of reuse of such Open Access shall be governed by a Creative Commons licence – details of these licences and which Creative Commons licence will apply to this Work are set out in our licence referred to above.

Other than as permitted in any relevant BMJ Author's Self Archiving Policies, I confirm this Work has not been accepted for publication elsewhere, is not being considered for publication elsewhere and does not duplicate material already published. I confirm all authors consent to publication of this Work and authorise the granting of this licence.

For peer review only

Are workplace factors associated with employee alcohol use? The WIRUS cross-sectional study

Mikkel Magnus Thørrisen[1,2]*
Jens Christoffer Skogen[2,3,4]
Tore Bonsaksen[5,6]
Lisebet Skeie Skarpaas[1]
Randi Wågø Aas[1,2]*

[1]Department of Rehabilitation Science and Health Technology, Faculty of Health Sciences, OsloMet – Oslo Metropolitan University, Oslo, Norway

[2]Department of Public Health, Faculty of Health Sciences, University of Stavanger, Stavanger, Norway

[3]Department of Health Promotion, Norwegian Institute of Public Health, Bergen, Norway

[4]Center for Alcohol & Drug Research, Stavanger University Hospital, Stavanger, Norway

[5]Department of Health and Nursing Sciences, Faculty of Social and Health Sciences, Inland Norway University of Applied Sciences, Elverum, Norway

[6]Department of Health, Faculty of Health Studies, VID Specialized University, Stavanger, Norway

*Correspondence:
Mikkel Magnus Thørrisen / Randi Wågø Aas
OsloMet – Oslo Metropolitan University, Faculty of Health Sciences, Department of Rehabilitation Science and Health Technology, PO box 4 St. Olavs plass, NO-0130 Oslo, Norway
mikkel-magnus.thorrisen@oslomet.no / randi.aas@oslomet.no

Word count: 6154 (main text, i.e., excluding title page, abstract, strength/limitations statement, declarations, reference list, tables, figure and supplementary material)

For peer review only

For peer review only - http://bmjopen.bmj.com/site/about/guidelines.xhtml

ABSTRACT

Objectives: Sociodemographic predictors of employee alcohol use are well established in the literature, but knowledge about associations between workplace factors and alcohol use is less explored. The aim of this study was to explore whether workplace factors were associated with employee alcohol use (consumption and alcohol-related problems).

Design: Cross-sectional study. Linear and binary logistic regression analyses.

Setting: Heterogeneous sample of employees (workers and supervisors) from 22 companies across geographical locations and work divisions in Norway.

Participants: Employees (N = 5388) responded on survey items measuring workplace factors and alcohol use.

Outcomes: Data on alcohol use were collected with the Alcohol Use Disorders Identification Test (AUDIT). Consumption was measured with the AUDIT-C (the first three items), and alcohol-related problems were operationalised as a sum score of eight or higher on the full ten-item AUDIT.

Results: Higher levels of alcohol consumption were associated with more liberal workplace drinking social norms (b = 1.37, p <.001), working full-time (b = .18, p <.001), working from holiday home (b = .40, p <.01), being a supervisor (b = .25, p <.001), having supervisors with less desired leadership qualities (b = -.10, p <.01), shorter working hours (b = -.03, p <.05), higher workplace social support (b = .13, p <.05), and higher income (b = .02, p <.001). Alcohol-related problems were associated with more liberal workplace drinking social norms (OR = 3.52, p <.001) and shorter working hours (OR = 0.94, p <.05).

Conclusions: Workplace drinking social norms was the supremely most dominant predictor of both consumption and alcohol-related problems. Results suggest that some workplace factors may play a role in explaining employee alcohol consumption, although the predictive

For peer review only - http://bmjopen.bmj.com/site/about/guidelines.xhtml

ability of these factors was limited. This study points to the importance of drinking social norms, workplace drinking culture and leadership for understanding employee alcohol use.

Keywords: Alcohol Drinking; Employees; Work; Workplace

Strengths and limitations of this study

- This study explores the importance of a wide range of workplace factors for employee alcohol use in a large and heterogeneous sample by means of a validated alcohol screening instrument
- This study utilises individuals' overall degree of consumption, as well as odds of having a drinking pattern that may induce alcohol-related problems, as outcomes
- Study limitations include a low response rate, the use of self-reported data, and a cross-sectional design that precludes causal inferences

INTRODUCTION

Alcohol consumption is associated with detrimental health outcomes.[1-4] Globally, approximately five percent of mortality and disability-adjusted life years (DALYs) can be attributed to alcohol consumption.[5] Within the age group of 25-49 years, 1.6 % of global DALYs were attributable to alcohol use disorders in 2019.[6] Risky drinking has been conceptualised as a drinking pattern that increases the risk of social, legal, medical, occupational, domestic and economic problems.[7] Reducing harmful alcohol use represents a keystone in sustainable development of health.[5, 8]

Alcohol is the most commonly used and misused psychoactive substance in the workforce.[9] Between 10 and 30 % of employees consume alcohol at a risky level and may benefit from alcohol prevention interventions.[10] Employees' alcohol use is associated with

For peer review only - http://bmjopen.bmj.com/site/about/guidelines.xhtml

For peer review only

increased sickness absence,[11-14] and presenteeism (impaired on-the-job performance).[15, 16] Research has demonstrated that brief alcohol interventions have promising effects in primary care,[17, 18] as well as in working populations.[19-21]

According to Frone's model of employee substance use,[22] employee alcohol use can be construed as a function of individual and workplace environment characteristics, and multifaceted interactions between them. Individual (sociodemographic) predictors of alcohol use among employees are quite well established in the research literature. For instance, being male, young, unmarried and not having children constitute individual predictors of elevated alcohol consumption.[10, 23, 24] The role of workplace factors is, however, not as established in the literature. Workplace factors may, on the one hand, refer to employment characteristics, i.e., to more general workplace factors conceptualised in the form of conditions and terms defining the work situation (e.g., working hours, work schedule, job position, job size and income). On the other hand, the workplace can be understood as a psycho-socio-cultural setting that involves job demands and support factors (which are related to occupational stress, e.g., psychological job demands, job control, workplace social support and supervisors' perceived leadership qualities), travels and worksite factors (related to degree of workplace social control, e.g., job travels, working from home and working from holiday home), and workplace drinking social norms (which reflect the workplace alcohol use climate, e.g., workplace drinking culture and drinking social norms).

Studies have demonstrated that workplace alcohol use climate may affect the drinking level of employees,[25-27] e.g., through the impact of injunctive and descriptive norms.[28] "Drinking social norms shared by a group define standards of appropriate behaviour, creating social controls that regulate workplace alcohol availability and drinking behaviours".[26, p. 602] For instance, in a large-scale study of employees in the USA (across workgroups and worksites) it was found that being part of an alcohol discouraging workplace drinking culture

For peer review only - http://bmjopen.bmj.com/site/about/guidelines.xhtml

predicted lower consumption (less frequent drinking in general and at work, as well as overall reduced likelihood of being a heavy drinker), compared to being part of a more encouraging workplace drinking culture.[26] Using multilevel analysis, it has been found that individual-level measurement of drinking social norms may be indicative of group-level norms at worksites employees are nested within.[26] Research on the importance of other aspects of the workplace and work situation, such as general employment characteristics, job demands and support factors and travels and worksite factors, stands out as more inconclusive.

Systematic reviews generally point to longer working hours being associated with higher alcohol consumption.[29, 30] However, the evidence for such a relationship remains uncertain. Some studies have failed to demonstrate statistically significant relationships,[31-33] and an opposite association (shorter working hours associated with higher risk of alcohol dependence) was found in a study of employees in Taiwan.[34] In a Norwegian study,[35] having flexible working hours was associated with increased consumption, and some studies have linked shift work with increased probability of workplace alcohol use,[31] as well as with higher overall consumption.[36] Others have not found a relationship between shift work and alcohol use.[35, 37-39] Evidence on the importance of work position is mixed. Some studies support an association between being a supervisor (as opposed to a worker) and higher alcohol consumption,[32, 40] while others do not.[23, 31, 34, 35] In a study of employees in the USA, Lee et al. revealed that working part-time (as opposed to full-time) was associated with higher alcohol use per week, but unrelated to prevalence of binge drinking episodes and alcohol-related consequences.[41] Although working hours may be somewhat overlapping with job size, one may capture different aspects of "time at work" by studying typical number of hours at work per day (working hours) and whether or not an individual has a full-time job (job-size). For instance, variations in working hours that exceed "normal working hours" (e.g., overtime) will not be captured in a dichotomy of part-time and full-time work. Studies have

For peer review only - http://bmjopen.bmj.com/site/about/guidelines.xhtml

suggested that higher socioeconomic status is associated with higher levels of alcohol consumption,[42, 43] which may be due to alcohol being a costly commodity.[44] As such, high-income employees may have better access to alcohol than low-income employees. A study of Canadian employees found that employees in high-income households had 35 % higher odds of high-risk drinking than employees in low-income households.[23] However, in a study of Japanese employees,[45] family income was unrelated to prevalence of alcohol-related problems.

Travels and worksite factors may refer to the degree to which employees are subjects of direct monitoring at the workplace. According to Roman and Trice,[46] low visibility at the workplace and low degrees of interdependence among employees stand out as risk factors for deviant drinking behaviours. In a study of employees in Norway,[40] working from home (telecommuting) at least two days per week was associated with higher alcohol use. Furthermore, a high prevalence of job travels has been linked with increased likelihood of problematic drinking.[47]

Research on the importance of job demands and support factors (e.g., leadership, job demands and control, and workplace social support) for employee alcohol use have yielded variable results. It has been advocated that visible and active supervisors constitute a protective factor for alcohol use among workers,[46] while others have failed to demonstrate such a relationship.[47, 48] In a Norwegian study,[49] supervisors' leadership style (transformational and laissez-faire leadership) was not related to employee alcohol use. On the other hand, some studies imply that alcohol use and hangover episodes are less prevalent at workplaces where supervisors exert active social control.[25, 50, 51] Analyses of cross-sectional data imply that lower levels of job control (skill discretion and decision latitude) may be associated with higher levels of alcohol consumption, although such a relationship has proved difficult to replicate in longitudinal data.[52] Some studies reject a potential

For peer review only - http://bmjopen.bmj.com/site/about/guidelines.xhtml

association between job control and alcohol use,[23, 34, 53] while others underscore that different types of control may be differentially related to consumption. For instance, Marchand found that an increased probability of risky drinking was associated with lower skill discretion and higher decision latitude,[32] while Saade and Marchand found the opposite.[33] Higher perceived psychological job demands have in some studies been related to higher alcohol use,[33, 34] but not in others.[54] In a study of Canadian employees,[55] lower psychological demands were associated with higher alcohol use for men only. The evidence base of physical job demands is also inconclusive. A Canadian study found that higher physical demands were associated with higher alcohol use among men only,[55] while a French study found an association for women only.[54] Another Canadian study failed to demonstrate a relationship between physical job demands and alcohol use altogether.[33] Some studies indicate that lower workplace social support is associated with higher alcohol consumption,[33, 38, 55] while others do not.[23, 53] A study from Norway found increased odds of alcohol-related problems among employees who reported a perceived imbalance between work efforts and rewards, in conjunction with high levels of work overcommitment.[56]

In summary, evidence on associations between workplace factors and employee alcohol use stands out as complex and nuanced. Moreover, few studies have explored the importance of workplace factors for alcohol consumption as well as for odds of alcohol-related problems in heterogeneous employee samples by means of validated alcohol screening instruments. Individuals' overall degree of consumption, and their odds of having a drinking pattern that may induce alcohol-related problems, constitute two different – yet equally important – ways of conceptualising alcohol use that can be differentially associated with workplace factors. Knowledge about associations between workplace factors and employee alcohol use is important as an aid in determining the extent to which employers should make workplace-

For peer review only - http://bmjopen.bmj.com/site/about/guidelines.xhtml

based alcohol health promotion and prevention interventions an overall priority, and for determining which elements should be emphasised in such interventions. Hence, further research is warranted.

Study aim

The aim of this study was to explore whether workplace factors (employment characteristics, job demands and support factors, travels and worksite factors and workplace drinking social norms) were associated with employee alcohol consumption and odds of having of alcohol-related problems in a heterogeneous sample of employees in Norway.

MATERIALS AND METHODS

Design and setting

This cross-sectional study explored associations between workplace factors and alcohol use in a heterogeneous sample of employees (workers and supervisors) in 22 companies in Norway (private companies: n = 8; central government companies: n = 6; local government companies: n = 8), across geographical locations. The study was part of the Norwegian national WIRUS project (Workplace Interventions preventing Risky alcohol Use and Sick leave).

Data collection and sample

The researchers recruited companies through three occupational health service units' client lists in collaboration with the addiction competence environment KoRus Stavanger. Individual-level inclusion criteria were: (i) age 16-72; (ii) status as employee, regardless of sector, work division or geographical location; and (iii) basic understanding of the Norwegian language. Our definition of employees (criteria ii) included white- (supervisors,

For peer review only - http://bmjopen.bmj.com/site/about/guidelines.xhtml

(semi)professional roles, business owners), blue- (labourers and skilled trade roles) and pink-collar employed (hospitality, retail, care and administration roles).[57] Inclusion of employees aged 16 to 72 (criteria i) was chosen because 16 constitutes the age when individuals in Norway have typically completed mandatory primary and secondary school and are eligible for full-time work (or further education), and because employment by Norwegian law may be terminated by the employer at age 72 (even though 67 constitutes the general age of retirement).[58] The invited sample was, in accordance with the inclusion criteria, heterogeneous, representing different sectors, work divisions and geographical locations. Twenty-two companies agreed to participate and provided e-mail addresses for all their employees (n = 30811). Digital questionnaires measuring workplace factors, alcohol use and sociodemographic variables were distributed to all employees in the 22 companies. Consent to participate was provided by 8542 employees (response rate = 27.7 %). A total of 3154 employees failed to respond on all relevant study items, leaving a final study sample of 5388 employees. Characteristics of the study sample are presented in Table 1.

Table 1. Characteristics of the study sample

Variable	
**Gender**	
Male, n (%)	1699 (31.5)
Female, n (%)	3689 (68.5)
**Age** (years), *M* (*SD*)	44.8 (11.2)
**Educational attainment**	
Primary/lower secondary, n (%)	87 (1.6)
Upper secondary, n (%)	1006 (18.7)
University/college ≤4 years, n (%)	2125 (39.4)
University/college >4 years, n (%)	2170 (40.3)
**Employment sector**	
Private corporation, n (%)	368 (6.8)
Local government, n (%)	3469 (64-4)
Central government, n (%)	1551 (28-8)
**Work division**	
Transportation/manufacturing, n (%)	285 (5.3)

For peer review only - http://bmjopen.bmj.com/site/about/guidelines.xhtml

Public administration/services, n (%)	4299 (79.8)
Health services, n (%)	762 (14.1)
Other services, n (%)	42 (0.8)

N = 5388; *M* = mean; *SD* = standard deviation

Data from participants were collected between October 2015 and October 2019. Hence, each participant was measured once (between 2015 and 2019), and all data were collected prior to the covid-19 pandemic. We predefined that a statistically satisfactory sample size had to conform with the formula N > 50 + (8 x number of predictors/covariates),[59] as well as exceeding a ratio of 15 participants per predictor/covariate variable.[60]

Measures

Employee alcohol use (outcomes)

The outcomes were measured with the Alcohol Use Disorders Identification Test (AUDIT). The AUDIT is an alcohol screening instrument, developed by the World Health Organization (WHO),[7, 61] consisting of 10 items (each scored 0-4). Higher scores on the AUDIT indicate higher consumption and more severe alcohol-related consequences. The first outcome, *alcohol consumption*, was measured with the first three AUDIT items (sum score of three items = continuous scale 0-12), often referred to as the AUDIT-C.[62] The second outcome, *alcohol-related problems*, was measured as a dichotomous categorical variable (0 = no problems; 1 = problems), based on a cut-off of eight on the full ten-item AUDIT (scores 0-7 = no problems; scores 8-40 = problems).[7]

Workplace factors (predictors)

Employment characteristics included *working hours* (number of hours during a typical day), *work schedule* (day job without weekends; day job with weekends; evening job; night

job; shift work), *job position* (worker; supervisor), *job size* (part-time; full-time), and *income* (gross annual household income in Norwegian Kroner (NOK)).

Job demands and support factors included employees' perceptions of *psychological job demands*, *job control*, *workplace social support* and supervisors' *leadership qualities*. Psychological job demands (five items), job control (perceived control over workplace decisions and potential for utilising personal skills in the job; nine items) and workplace social support (from co-workers and supervisors; eight items) were measured with the Job Content Questionnaire (JCQ) (63),[58] based on the Demand Control Support model,[64, 65] each scored on four-point Likert scales where higher scores indicated higher levels of demands, control and support, respectively. Reversed items were recoded and mean scores were calculated (demands: $\alpha = .76$; control: $\alpha = .58$; support: $\alpha = .89$). Supervisors' leadership qualities were measured with the Seven-item Leadership Qualities Questionnaire (7LQQ).[66] Developed on the basis of qualitative interview data,[67] 78 distinct leadership qualities were identified and categorised into seven desired leadership types (problem-solver; contact-maker; responsible-maker; protector; trust-creator; recogniser; encourager), each type scored on a five-point Likert scale based on the frequency of which supervisors were perceived to display the leadership type in question (0 = never; 1 = seldom; 2 = sometimes; 3 = often; 4 = very often). A mean score of the seven items was calculated ($\alpha = .94$), where higher scores indicated higher levels of desired leadership qualities.

Travels and worksite factors (reflecting degree to which employees are under direct monitoring by co-workers and supervisors) were measured in terms of whether the job included *job travels* (no; yes), *working from home* (no; yes), and *working from holiday home* (no; yes).

Workplace alcohol use climate was conceptualised as employees' perceptions of *drinking social norms*, measured with the four items from the Drinking Norms Scale (DNS) that

For peer review only

For peer review only - http://bmjopen.bmj.com/site/about/guidelines.xhtml

directly relates to the work situation.[26] Responses were provided on a four-point Likert scale where higher scores indicated more liberal drinking social norms. A mean score was calculated (α = .73).

An overview of workplace factors is presented in Table 2.

Table 2. Overview of workplace factors

Main factor	Factors	Measurement
Employment characteristics	Working hours	Continuous[A]: number of hours during a typical day
	Work schedule	Categorical[A]: day job, no weekends; day job with weekends; evening job; night job; shift work
	Job position	Categorical[A]: worker; supervisor
	Job size	Categorical[A]: part-time; full-time
	Income	Continuous[A]: gross annual household income in 100000 NOK
Job demands and support factors	Psychological job demands	JCQ: mean score of five items
	Job control	JCQ: mean score of nine items
	Workplace social support	JCQ: mean score of eight items
	Supervisors' leadership qualities	7LQQ: mean score of seven items
Travels and worksite factors	Job travels	Categorical[A]: no; yes
	Working from home	Categorical[A]: no; yes
	Working from holiday home	Categorical[A]: no; yes
Workplace drinking social norms	Drinking social norms	DNS: mean score of four items

[A]Self-developed measure; NOK = Norwegian Kroner; JCQ = Job Content Questionnaire; 7LQQ = Seven-item Leadership Qualities Questionnaire; DNS = Drinking Norms Scale

Covariates

The following sociodemographic factors, that have demonstrated significant associations with alcohol use in a Norwegian employee population,[10] were included as controls in adjusted analyses: *Gender* (male; female), *age* (years), *educational attainment* (primary/lower secondary; upper secondary; university/college ≤4 years; university/college >4 years), *living status* (living alone; living with others), *marital status* (unmarried; married), and *number of children*. Due to the cross-sectional data in this study being collected over a period of

For peer review only

approximately five years, *year of data collection* (2015; 2016; 2017; 2018; 2019) was included as an additional covariate.

Analysis

Descriptive analyses were performed on the main study variables (outcomes and predictors) and presented in terms of means, standard deviations, frequencies and percentages, as appropriate.

Main analyses were conducted in two stages: model selection and predictor comparison.[68] In the model selection stage, associations between workplace factors and alcohol consumption (AUDIT-C scale 0-12) were explored by means of a multiple hierarchical linear regression analysis. Employment characteristics were entered as predictors in the first model, followed by sequential inclusion of job demands and support factors (Model 2), travels and worksite factors (Model 3), workplace drinking social norms (Model 4), and covariates (Model 5). Unstandardised (b) and standardised (β) regression coefficients were calculated. Potential multicollinearity was explored using the variance inflation factor (VIF). Multicollinearity was deemed a concern if VIFs exceeded 10.[69] Associations between workplace factors and the odds of alcohol-related problems (AUDIT-10 categories 0-7 vs. 8-40) were explored with a multiple hierarchical binary logistic regression analysis. Predictors and covariates were entered sequentially through five models, and odds ratios were calculated. Goodness of fit was explored by means of Hosmer and Lemeshow tests, where poor model fit was indicated by $p < .05$.[70] In the final and fully adjusted models in the regression analyses (linear and logistic), associations between workplace factors and alcohol outcomes were adjusted for gender, age, educational attainment, living status, marital status, number of children and year of data collection. Work schedule (categorical nominal predictor) was dummy coded. All main analyses were performed with IBM SPSS version 27.

For peer review only - http://bmjopen.bmj.com/site/about/guidelines.xhtml

In the predictor comparison stage, the relative importance of predictors that demonstrated significant associations with the outcomes (p <.05) in fully adjusted models in the model selection stage were estimated by means of dominance analysis (DA).[71] DA consists of examining R^2 values for all possible subset models in a multiple regression, with additional contribution of each predictor. A predictor is considered to be more important than its competitors if its predictive ability exceeds the others' in all subset regressions.[71, p. 545] Each predictor's relative importance was expressed in terms of its average contribution (in percentage points) to the explained variance in the outcome. DA was conducted separately for each outcome using Stata version 17.

Significant results were defined as p <.05.

Patient and public involvement

Employee representatives and other relevant stakeholders were included in the WIRUS project's reference group. Design, research questions, recruitment procedures and choice of outcome measures in the project have been informed by priorities, experience and preferences expressed by the reference group.

RESULTS

Approximately one out of ten employees (11.5 %) reported alcohol-related problems (AUDIT ≥8). The sample's means score on alcohol consumption was 3.2 (on the AUDIT-C scale ranging from 0 to 12). The majority of employees were workers (79.6 % of the total sample) with full-time (81.8 % of the total sample) day job schedules without weekends (73.9 % of the total sample). Employees' average gross annual household income was 1,012,000 NOK (approximately 83,587 GBP), and a minority of employees had a job that included job travels (14.9 % of the total sample), working from home (6.9 % of the total sample) and

For peer review only - http://bmjopen.bmj.com/site/about/guidelines.xhtml

working from holiday home (3.0 % of the total sample). Job control and workplace social support were rated higher than psychological job demands (on the JCQ scales ranging from 1 to 4: $M_{\text{Job control}} = 3.0$; $M_{\text{Social support}} = 3.2$; $M_{\text{Psychological job demands}} = 2.2$). Employees' mean rating of supervisors' desired leadership qualities was 2.9 (on the 7LQQ scale ranging from 0 to 4). On average, employees reported somewhat more restrictive than liberal workplace drinking social norms (*M* = 2.2 on the DNS scale ranging from 1 to 4). Descriptive statistics for the main study variables are presented in Table 3.

Table 3. Descriptive statistics for the main study variables

	Variable	
O	Alcohol consumption[A], *M* (*SD*)	3.21 (1.87)
O	Alcohol-related problems[B], n (%) yes	619 (11.50)
**Employment characteristics:**		
P	Working hours[C], *M* (*SD*)	7.95 (1.83)
P	Day job, no weekends, n (%) yes	3982 (73.90)
P	Evening job, n (%) yes	22 (0.40)
P	Night job, n (%) yes	84 (1.60)
P	Shift work, n (%) yes	981 (18.20)
P	Job position, n (%) workers	4288 (79.60)
P	Job size, n (%) full-time	4407 (81.80)
P	Income[D], *M* (*SD*)	10.12 (6.25)
**Job demands and support factors:**		
P	Psychological job demands[E], *M* (*SD*)	2.17 (0.43)
P	Job control[E], *M* (*SD*)	3.00 (0.34)
P	Workplace social support[E], *M* (*SD*)	3.19 (0.47)
P	Leadership qualities[F], *M* (*SD*)	2.91 (0.81)
**Travels and worksite factors:**		
P	Job travels, n (%) yes	804 (14.90)
P	Working from home, n (%) yes	374 (6.90)
P	Working from holiday home, n (%) yes	161 (3.00)
**Workplace drinking social norms:**		
P	Drinking social norms[G], *M* (*SD*)	2.23 (0.53)

N = 5388; O = outcome; P = predictor; *M* = mean; *SD* = standard deviation; [A]Alcohol Use Disorders Identification Test – Consumption (AUDIT-C), scale 0-12; [B]AUDIT (all ten items, scale 0-40), sum score ≥8; [C]Number of hours during a typical day; [D]Gross annual household income in 100,000 Norwegian Kroner; [E]Job Content Questionnaire (JCQ), scale 1-4; [F]Seven-item Leadership Qualities Questionnaire (7LQQ), scale 0-4; [G]Drinking Norms Scale (DNS), scale 1-4 (higher scores = more liberal norms)

For peer review only - http://bmjopen.bmj.com/site/about/guidelines.xhtml

Bivariate correlations between all study variables (outcomes, predictors and covariates) are presented in the Supplementary File (Table S1).

Workplace factors and alcohol consumption

Associations between workplace factors and alcohol consumption are presented in Table 4.

For peer review only - http://bmjopen.bmj.com/site/about/guidelines.xhtml

Table 4. Associations between workplace factors and alcohol consumption[A]

	Model 1 Employment characteristics	Model 2 Job demands and support factors added	Model 3 Travels and worksite factors added	Model 4 Workplace drinking social norms added	Model 5 Fully adjusted model, including covariates[B]
	b (β) [*p*]	b (β) [*p*]	b (β) [*p*]	b (β) [*p*]	b (β) [*p*]
Working hours	-.01 (-.00) [.750]	.00 (.00) [.905]	-.00 (-.00) [.831]	**-.03 (-.03) [.029]**	**-.03 (-.03) [.049]**
Day job, no weekends[C]	**-.25 (-.06) [.022]**	**-.28 (-.07) [.011]**	**-.25 (-.06) [.020]**	-.12 (-.03) [.231]	-.04 (-.01) [.669]
Evening job[C]	.41 (.01) [.320]	.39 (.01) [.346]	.43 (.01) [.303]	.21 (.01) [.574]	.07 (.00) [.840]
Night job[C]	.02 (.00) [.920]	-.06 (-.00) [.798]	-.02 (-.00) [.924]	.12 (.01) [.590]	-.04 (-.00) [.867]
Shift work[C]	.06 (.01) [.619]	.04 (.01) [.745]	.07 (.02) [.550]	.09 (.02) [.441]	-.06 (-.01) [.591]
Job position[D]	.11 (.02) [.109]	**.14 (.03) [.040]**	.10 (.02) [.118]	**.25 (.06) [<.001]**	**.25 (.06) [<.001]**
Job size[E]	**.46 (.09) [<.001]**	**.46 (.09) [<.001]**	**.44 (.09) [<.001]**	**.31 (.06) [<.001]**	**.18 (.04) [.006]**
Income[F]	.00 (.00) [.760]	.00 (.01) [.716]	.00 (.00) [.966]	.00 (.00) [.840]	**.02 (.05) [<.001]**
Psych. job demands[G]	-	**-.15 (-.03) [.017]**	**-.15 (-.03) [.016]**	**-.13 (-.03) [.026]**	-.09 (-.02) [.106]
Job control[G]	-	-.12 (-.02) [.144]	-.15 (-.03) [.063]	-.10 (-.02) [.165]	.02 (.00) [.755]
Workplace social support[G]	-	**.15 (.04) [.030]**	**.16 (.04) [.023]**	**.14 (.03) [.035]**	**.13 (.03) [.040]**
Leadership qualities[H]	-	**-.09 (-.04) [.028]**	**-.09 (-.04) [.033]**	**-.10 (-.04) [.005]**	**-.10 (-.04) [.004]**
Job travels[I]	-	-	**.25 (.05) [.001]**	.04 (.01) [.564]	.04 (.01) [.575]
Working from home[I]	-	-	-.05 (-.01) [.647]	-.18 (-.02) [.069]	-.12 (-.02) [.216]
Working from holiday home[I]	-	-	**.40 (.04) [.012]**	**.42 (.04) [.003]**	**.40 (.04) [.007]**
Drinking social norms[J]	-	-	-	**1.51 (.42) [<.001]**	**1.37 (.39) [<.001]**
R^2	.012	.014	.018	.192	.225
VIF range	1.02 – 3.54	1.03 – 3.56	1.03 – 3.59	1.03 – 3.60	1.08 – 3.62

N = 5388; Results from multiple hierarchical linear regression analyses; b = unstandardised regression coefficient; β = standardized regression coefficient; VIF = variance inflation factor; [A]Alcohol Use Disorders Identification Test – Consumption (AUDIT-C), scale 0-12, higher scores indicate higher consumption; [B]Covariates = gender, age, educational attainment, living status, marital status, number of children, year of data collection; [C]Ref. = other work schedules; [D]Ref. = worker; [E]Ref. = part-time; [F]Household gross annual income in 100,000 Norwegian Kroner; [G]Job Content Questionnaire (JCQ), scale 1-4, higher scores indicate higher predictor values; [H]Seven-item Leadership Qualities Questionnaire (7LQQ), scale 0-4, higher scores indicate higher level of desired leadership qualities; [I]Ref. = no; [J]Drinking Norms Scale (DNS), scale 1-4, higher scores indicate more liberal workplace drinking norms; Bold typeface indicates statistically significant associations (p <.05)

For peer review only - http://bmjopen.bmj.com/site/about/guidelines.xhtml

As shown in Table 4 (Models 1-3), employment characteristics, job demands and support factors and travels and worksite factors explained only small proportions of the variance in alcohol consumption (1.2 to 1.8 %). The proportion of explained variance in consumption increased considerably (to 19.2 %) when workplace drinking social norms was included in the analysis (Model 4). In the fully adjusted model (Model 5), also taking covariates into account, eight workplace factors were significantly associated with alcohol consumption. The highest VIF was 3.62, indicating that multicollinearity was not a concern.

Higher consumption was associated with shorter working hours, being a supervisor (versus a worker), working full-time (as opposed to part-time), higher income, higher levels of workplace social support, having a supervisor with less desired leadership qualities, working from holiday home, and more liberal workplace drinking social norms. The remaining workplace factors either failed to demonstrate significant relationships with alcohol consumption altogether (evening job schedule, night job schedule, shift work schedule, job control, working from home), or failed to reach statistical significance in the fully adjusted model (day job without weekend schedule, psychological job demands, job travels).

The relative importance of the eight significant workplace factors for explaining the variance in alcohol consumption is presented in Table 5.

Table 5. Relative importance of workplace factors[A] in explaining alcohol consumption[B]

Predictor	Dominance statistic	Average contribution (pp)[C]	Ranking
Drinking social norms	0.1792	17.92	1
Job size	0.0045	0.45	2
Working from holiday home	0.0016	0.16	3
Job position	0.0013	0.13	4
Leadership qualities	0.0007	0.07	5
Working hours	0.0005	0.05	6
Workplace social support	0.0002	0.02	7
Income	0.0000	0.00	8

N = 5388; Results from dominance analysis; [A]Workplace factors that demonstrated significant associations with alcohol consumption in the fully adjusted regression model (Model 5 in Table 4); [B]Alcohol Use

For peer review only - http://bmjopen.bmj.com/site/about/guidelines.xhtml

Disorders Identification Test – Consumption (AUDIT-C), scale 0-12; [C]Average contribution to the R^2 in percentage points

Based on average contribution to the explained variance in alcohol consumption, drinking social norms (17.92 %) was the dominant predictor, followed by job size (0.45 %), working from holiday home (0.16 %), job position (0.13 %), leadership qualities (0.07 %), working hours (0.05 %), workplace social support (0.02 %) and income (0.00 %). In the fully adjusted regression model (Table 4, Model 5), a one-unit increase in drinking social norms (towards more liberal) was associated with an increase of 1.37 units on the alcohol consumption scale (ranging from 0 to 12).

Workplace factors and alcohol-related problems

Associations between workplace factors and odds of having alcohol-related problems are presented in Table 6.

For peer review only

For peer review only - http://bmjopen.bmj.com/site/about/guidelines.xhtml

Table 6. Associations between workplace factors and odds of having alcohol-related problems[A]

	Model 1 Employment characteristics	Model 2 Job demands and support factors added	Model 3 Travels and worksite factors added	Model 4 Workplace drinking social norms added	Model 5 Fully adjusted model, including covariates[B]
	OR (*p*)	OR (*p*)	OR (*p*)	OR (*p*)	OR (*p*)
Working hours	0.97 (.270)	0.98 (.315)	0.97 (.247)	**0.95 (.025)**	**0.94 (.024)**
Day job, no weekends[C]	0.74 (.079)	0.73 (.074)	0.74 (.082)	0.82 (.278)	0.97 (.852)
Evening job[C]	2.35 (.117)	2.30 (.128)	2.35 (.119)	1.98 (.235)	1.77 (.334)
Night job[C]	1.59 (.212)	1.48 (.299)	1.52 (.268)	1.68 (.182)	1.48 (.328)
Shift work[C]	1.05 (.812)	1.02 (.921)	1.04 (.822)	1.06 (.761)	0.83 (.373)
Job position[D]	0.82 (.082)	0.83 (.107)	0.80 (.067)	0.95 (.646)	0.99 (.956)
Job size[E]	**1.92 (<.001)**	**1.91 (<.001)**	**1.88 (<.001)**	**1.68 (<.001)**	1.31 (.064)
Income[F]	**1.00 (.001)**	**1.00 (.001)**	**1.00 (.001)**	**1.00 (<.001)**	1.00 (.764)
Psych. job demands[G]	-	1.02 (.880)	1.02 (.869)	1.03 (.780)	1.05 (.672)
Job control[G]	-	0.82 (.142)	0.79 (.086)	0.82 (.175)	0.92 (.565)
Workplace social support[G]	-	0.97 (.764)	0.97 (.812)	0.90 (.407)	0.88 (.331)
Leadership qualities[H]	-	0.94 (.317)	0.94 (.329)	0.92 (.223)	0.90 (.127)
Job travels[I]	-	-	**1.35 (.016)**	1.12 (.383)	1.09 (.506)
Working from home[I]	-	-	0.99 (.964)	0.86 (.435)	0.91 (.646)
Working from holiday home[I]	-	-	1.09 (.749)	1.15 (.599)	1.17 (.564)
Drinking social norms[J]	-	-	-	**4.61 (<.001)**	**3.52 (<.001)**
$R^2_{Nagelkerke}$	.020	.022	.025	.125	.184
Hosmer and Lemeshow test	$\chi = 12.39$, $p = .135$	$\chi = 13.63$, $p = .092$	$\chi = 6.21$, $p = .624$	$\chi = 9.39$, $p = .310$	$\chi = 8.22$, $p = .412$

N = 5388; Results from multiple binary logistic regression analyses; OR = odds ratio; [A]Alcohol Use Disorders Identification Test (AUDIT, all ten items), sum score 0-7 (no problems) vs 8-40 (problems); [B]Covariates = gender, age, educational attainment, living status, marital status, number of children, year of data collection; [C]Ref. = other work schedules; [D]Ref. = worker; [E]Ref. = part-time; [F]Household gross annual income in 100,000 Norwegian Kroner; [G]Job Content Questionnaire (JCQ), scale 1-4, higher scores indicate higher predictor values; [H]Seven-item Leadership Qualities Questionnaire (7LQQ), scale 0-4, higher scores indicate higher level of desired leadership qualities; [I]Ref. = no; [J]Drinking Norms Scale (DNS), scale 1-4, higher scores indicate more liberal workplace drinking norms; Bold typeface indicates statistically significant associations (p <.05)

For peer review only - http://bmjopen.bmj.com/site/about/guidelines.xhtml

As shown in Table 6 (Models 1-3), employment characteristics, job demands and support factors and travels and worksite factors explained only small proportions of the variance in odds of having alcohol-related problems (2.0 to 2.5 %). The explained variance increased considerably when workplace drinking social norms were included in the analysis (Model 4, 12.5 %). In the fully adjusted model (Model 5, containing all workplace factors as well as covariates), only two workplace factors were significantly related with odds of having alcohol-related problems. There was no evidence of poor model fits, as indicated by Hosmer and Lemeshow tests reaching $p \geq .05$.

Elevated odds of alcohol-related problems was associated with shorter working hours and more liberal workplace drinking social norms. The remaining workplace factors either failed to reach statistical significance in the fully adjusted model (job size, income, job travels, working from home), or were unrelated with alcohol-related problems altogether (day job no weekend schedule, evening schedule, night schedule, shift work schedule, job position, psychological job demands, job control, workplace social support, leadership qualities, working from holiday home).

Dominance analysis (Table 7) revealed that drinking social norms was the dominant predictor of alcohol-related problems by having an average contribution to the proportion of explained variance of 5.26 %. In contrast, the contribution of working hours was only 0.04 % (on average).

Table 7. Relative importance of workplace factors[A] in explaining odds of having alcohol-related problems[B]

Predictor	Dominance statistic	Average contribution (pp)	Ranking
Drinking social norms	0.0526	5.26	1
Working hours	0.0004	0.04	2

N = 5388; Results from dominance analysis; [A]Workplace factors that demonstrated significant associations with odds of having alcohol-related problems in the fully adjusted regression model (Model 5 in Table 6); [B]Alcohol Use Disorders Identification Test (AUDIT, all ten items), sum score 0-7 (no problems) vs 8-40 (problems); [C]Average contribution to the R^2 in percentage points

For peer review only - http://bmjopen.bmj.com/site/about/guidelines.xhtml

A one-hour increase in working hours was associated with a reduced odds of alcohol-related problems by a factor of 0.94. A one-unit increase on the drinking social norms scale (towards more liberal) was associated with an increased odds of alcohol-related problems by a factor of 3.52.

Summary of main results

Main results are summarised in Figure 1.

[Figure 1 about here]

Eight out of 16 workplace factors were not significantly related to either alcohol consumption or alcohol-related problems. Six workplace factors were significantly associated with alcohol consumption but not with alcohol-related problems (job position, job size, income, workplace social support, leadership qualities, working from holiday home). Two workplace factors were significantly related to both alcohol consumption and alcohol-related problems (working hours, drinking social norms). With the exception of drinking social norms (that was the dominant predictor of alcohol consumption as well as of alcohol-related problems), associations between workplace factors and the alcohol outcomes were quite weak (see Tables 4 and 6) and most of the variance in employees' alcohol consumption and alcohol-related problems was explained by variables not included in this study, i.e., by variables beyond the included workplace factors.

DISCUSSION

For peer review only - http://bmjopen.bmj.com/site/about/guidelines.xhtml

The aim of this study was to explore whether workplace factors were associated with alcohol use in a heterogeneous sample of employees in Norway.

Drinking social norms emerged as the supremely most important predictor of both alcohol consumption and alcohol-related problems. More liberal attitudes toward the role of alcohol in workplace settings was associated with having a higher consumption as well as elevated odds of having alcohol-related problems. Drinking norms prescribe the level of consumption that is considered appropriate or tolerable in specific social contexts, and employees' drinking norms may reflect the workplace drinking culture.[26, 72-74] Hence, our study supports previous research demonstrating that workplace alcohol use climate may affect employees' level and experienced consequences of alcohol consumption.[25-28] The association between workplace drinking norms and alcohol consumption can be viewed in conjunction with evidence that has demonstrated that a significant proportion of employees' overall consumption indeed does occur in job-related contexts. Studies from Norway have found that 30 % of employees had consumed alcohol in job-related settings during the past two weeks,[75] 43 % of employees' overall consumption occurred in job-related situations,[75] and employers initiated and organised more than half of the job-related situations in which employees were exposed to alcohol.[76]

Interestingly, longer work hours were associated with lower consumption but also with higher odds of having alcohol-related problems. These findings may, intuitively, stand out as somewhat contradictory, insofar that one may expect alcohol consumption to be closely associated with alcohol-related problems. We did find a considerable and statistically significant correlation between the two alcohol outcomes. However, the strength of the correlation was in the upper layer of moderate strength (see Supplementary File, Table S1), which is far from a perfect correlation. Hence, somewhat different mechanisms may underlie the two alcohol outcomes, which has been demonstrated in earlier research. As captured by

For peer review only - http://bmjopen.bmj.com/site/about/guidelines.xhtml

the term the alcohol harm paradox, certain populations may be exposed to a variety of health risks that interact with alcohol consumption to create synergistically detrimental effects of consumption,[77] which result in greater alcohol-related harm among individuals with specific characteristics (e.g., socioeconomic variables) for each consumed alcohol unit, compared with individuals without these specific characteristics.[24] Associations between working hours and alcohol outcomes have been explained in terms of stress.[29, 34, 78] Shorter working hours may be related to higher consumption (as found in our study) due to involuntary part-time and/or temporary contracts generating stress that is coped with by means of higher alcohol consumption.[34] On the other hand, particularly long working hours may be associated with particularly high work loads and stress that result in coping mechanisms generating alcohol-related problems.[34, 78] However, our study did not allow causal explanations for the differential findings regarding the relationships between working hours, consumption and alcohol-related problems. It should be noted that associations found in our study were quite weak and that we, in line with earlier researchers, were unable to establish consistent knowledge regarding the relationship between working hours and alcohol outcomes among employees.[29-34]

Higher income was associated with higher alcohol consumption. This finding is consistent with earlier studies emphasising that higher socioeconomic status is related to higher levels of consumption.[23, 42, 43] Alcohol is a costly commodity,[44] and high-income employees have better access to alcohol than their lower-income counterparts.

Working full-time (as opposed to part-time) was associated with elevated alcohol consumption among employees. This finding contradicts earlier research from the USA that has found that part-time workers had higher weekly consumption than full-time workers.[41] Our data do not illuminate reasons for why full-time employees were more exposed to alcohol than part-time employees. One may hypothesise that this association can be interpreted in

For peer review only - http://bmjopen.bmj.com/site/about/guidelines.xhtml

terms of the degree to which employees are socially integrated at the workplace. Part-time employees may have a lower workplace social integration than their full-time counterparts, perhaps resulting in a lower exposure to alcohol in job-related situations. Alternatively, part-time employees may have reduced working hours due to health problems,[79] which in turn may explain why they – on a group level – consume less alcohol than full-time employees. Employees' financial situation may as well be of importance, insofar that alcohol is a costly commodity,[44] and that part-time workers may find themselves in a more strained financial situation than full-time employees.

Workers consumed less alcohol than supervisors, which is in line with some earlier studies.[32, 40] One may hypothesise that a supervisor position entails access and exposure to factors that may enhance alcohol consumption, such as higher income and greater work location flexibility (e.g., working from holiday home). However, our data do not lend themselves to explanations for the association between job position and alcohol consumption, and several studies have failed to demonstrate such a relationship.[23, 31, 34, 35]

Worksite factors (e.g., working from home and having overall greater flexibility) and job travels have in some studies been linked with elevated alcohol consumption.[35, 40, 47] In our study, travels and worksite factors demonstrated mixed results. We did not find an association between alcohol consumption, working from home and job travels. However, working from holiday home was associated with elevated consumption. These findings may reflect a context-dependency in the relationship between work location flexibility and alcohol use. In an earlier study exploring relationships between activity patterns and alcohol use among employees in Norway,[80] non-domestic activities were associated with higher consumption than domestic activities. Having an activity pattern characterised by activities within (rather than outside) the household was associated with lower alcohol consumption. Working from home (in a domestic setting) may constitute working in an activity setting not

For peer review only - http://bmjopen.bmj.com/site/about/guidelines.xhtml

very compatible with alcohol use, even though working from home entails being less visible for co-workers and supervisors and less susceptible to workplace social control (factors which have been linked with elevated alcohol consumption).[9, 22] Working from holiday home constitutes a non-domestic activity where work is performed in a context more similar to vacations. Vacations and holidays typically involve greater alcohol exposure, potentially resulting in higher consumption levels.[81, 82]

Having supervisors with desired leadership qualities was associated with lower alcohol consumption. Supervisors who are highly rated on desired leadership qualities (problem-solving, contact-making, responsible-making, protecting, trust-creating, recognising and encouraging;[67]) may be perceived as active, visible and involved with their staff and can be well-positioned to set norms and exert informal social control. It has been advocated that visible and active supervisors represent protective factors for elevated alcohol use among workers,[46] and studies have demonstrated lower prevalence of drinking and hangover episodes at work among employees employed at workplaces where supervisors exert active social control.[25, 50, 51] As such, supervisors with desired leadership qualities may be influential in determining workplace drinking cultures. One may assume that desired leadership qualities represent a potential protective factor for elevated alcohol consumption in line with how earlier studies have found such desired leadership to be a protective factor for mental distress,[83] and physiological pain.[84, 85]

In contrast to earlier studies that have found that higher levels of social support at work are associated with lower alcohol consumption among employees,[33, 38, 55] we found an association in the opposite direction, i.e., that higher levels of support were associated with higher consumption. Social support may be conceptualised as a proxy of social integration,[86] and our measure of social support included support from both peers and supervisors. Perceived levels of social support may reflect the level of which the individual

For peer review only - http://bmjopen.bmj.com/site/about/guidelines.xhtml

employee feels socially integrated in the workplace culture. Hence, social support may have dual and opposing effects on alcohol outcomes, depending on the workplace culture. In liberal drinking cultures, high levels of perceived support may reinforce permissive drinking norms,[87] while the opposite may be true in more restrictive workplace drinking cultures.

Methodological considerations

The cross-sectional design of this study precludes causal inferences regarding the relationships between workplace factors and alcohol-related outcomes. Even though we attempted to minimise risk of confounding by including relevant covariates in adjusted analyses, reversed, reciprocal and/or third-variable causation cannot be ruled out. For instance, the association between job size and alcohol use may be due to job size affecting drinking pattern, to drinking pattern affecting job size, or to an unmeasured third variable (e.g., health status) affecting both job size and drinking pattern. However, the aim of this study was not to unravel causal relationships but was explorative in nature. The cross-sectional design may also have played a role in explaining why observed effect sizes were quite small. Potential effects of some workplace factors on broad indicators of alcohol use and problems may be temporary and/or short-lived, rendering it difficult to fully capture true effects without more robust research designs that involve longitudinal measures.

The sample was large and consisted of a heterogeneous selection of employees across work sectors, divisions, levels and geographical locations. The sample size well exceeded the a priori defined criteria,[59, 60] and was thus considered statistically satisfactory (required sample size: N = 435; actual sample size: N = 5388). However, the response rate was quite low (27.7 %). Low response rates may represent a threat to external validity by limiting generalisability of results in instances where responders systematically differ from non-responders. Study selection analyses of the WIRUS screening data indicate that the final study

For peer review only - http://bmjopen.bmj.com/site/about/guidelines.xhtml

sample was similar to the invited sample (i.e., all employees in the included companies) regarding distributions of gender and age.[88] However, compared with the entire Norwegian workforce, female, older and well-educated employees were overrepresented in our study. Moreover, it must be noted that public sector employees were largely overrepresented, resulting in a limited potential of generalising results across employment sectors. Probable reasons for this overrepresentation include that public companies in Norway, on average, tend to be larger than private companies (the project aimed to recruit companies with >100 employees), and that an economic downturn in Norway in 2014-2015 (due to falling oil prices) made it difficult for private (profit-based) companies to prioritise participation in research. There may also be important differences between the study sample and non-responders that we lack information about (unmeasured selection effects) that have biased our findings. Moreover, a considerable number of employees (n = 3154) were excluded due to not responding on all study items in the survey. Comparisons between completers and non-completers on sociodemographic variables (see Supplementary File, Table S2) demonstrated that completers were characterised by a slight overrepresentation of males, employees with university/college education, married employees, and employees having more children.

This study was solely based on self-reported data from employees, which may involve risks of measuring bias (e.g., recall bias and social desirability bias). In particular, one may assume that alcohol use may have been underestimated, insofar that studies have demonstrated a discrepancy between self-reported consumption and actual alcohol sales.[89] On the other hand, alcohol consumption and alcohol-related problems were both measured with the AUDIT, which has demonstrated psychometric properties superior to other alcohol screening instruments,[90] and has been deemed appropriate for use in Norwegian employee populations.[91] A threshold of eight points on the AUDIT as indicative of alcohol-related

For peer review only

problems has been found to represent a satisfactory compromise between sensitivity and specificity,[7] and are in line with previous research on non-clinical populations.[92-95]

Implications

Among the workplace factors explored in this study, drinking social norms emerged as the supremely most important predictor of employee alcohol use. Hence, results from our study calls attention to the importance of workplace drinking cultures to understand employees' level of alcohol consumption and odds of experiencing alcohol-related problems. Furthermore, other significant predictors of employee alcohol use support the notion of emphasising workplace drinking culture. Perceiving higher levels of social support and being full-time employed were both associated with higher consumption levels and can both be conceptualised as proxies of social integration at the workplace, thereby indicating the importance of workplace culture. Moreover, results indicate the importance of supervisor behaviour. Supervisors consumed more alcohol than workers, and having supervisors with high levels of desired leadership qualities was associated with lower levels of consumption among employees. Efforts aimed at reducing alcohol consumption and alcohol-related problems among employees may include fostering less liberal drinking cultures at workplaces in combination with desired leadership qualities among managers. Raising managers' awareness of their influence on workplace drinking culture may be serviceable. Prosocial supervisors may stimulate workplace cultures that discourage or inhibit drinking in work-related situations and may also foster social relationships and trust that enable establishment of routines for early identification and aid for employees who may benefit from alcohol prevention interventions.

In a systematic review,[96] collaboration/teamwork and positive, accessible and fair supervisors were identified among the most pronounced factors considered as important for a

For peer review only - http://bmjopen.bmj.com/site/about/guidelines.xhtml

healthy workplace. In another systematic review,[97] covering workplace resources to improve employee well-being and performance, social support and high-quality relationships between workers and supervisors were identified among the factors organisations should emphasise. As such, a focus on workplace factors identified in our study as potential protective factors for alcohol-related problems seem to converge with the WHO framework for healthy workplaces, i.e., workplaces "in which workers and managers collaborate to use a continual improvement process to protect and promote health, safety and well-being of all workers and the sustainability of the workplace by considering" important aspects such as the psychosocial work environment, the organisation of work, and the workplace culture.[98, p. 16]

Evidence on associations between workplace factors and employee alcohol use still stands out as complex and nuanced, and this study showed that only marginal proportions of the variance in alcohol consumption and alcohol-related problems were explained by employment characteristics, job demands and support factors and travels and worksite factors. Hence, further research is warranted. Results from this study suggest the application and further development of interventions, e.g., Workplace Health Promotion Programs (WHPP), targeting workplace drinking culture and leadership. Effects and implementation of such interventions should be explored by means of robust research designs, such as prospective cohort studies and cluster randomised controlled trials. Future research may also benefit from explorations of potential interactions between individual and workplace factors, including mediators and moderators, which were beyond the scope of our study.

Conclusions

Knowledge about workplace factors associated with employee alcohol use is important as an aid in determining the extent to which employers should make workplace-based alcohol

For peer review only - http://bmjopen.bmj.com/site/about/guidelines.xhtml

prevention interventions an overall priority. This study, conducted in a large and heterogeneous sample of employees in Norway points to the importance of drinking social norms, workplace drinking culture and leadership.

DECLARATIONS

Ethics approval statement

Companies and employees were provided with information about the study's aim, they were assured confidentiality, that participation was voluntary, and that they could withdraw from the study at any given time. Written informed consent was obtained from all included employees. The study was approved by the Regional Committee for Medical and Health Research in Norway (REK; approval no. 2014/647).

Author contributions

RWA is the principal investigator (PI) and project manager (PM) for the WIRUS project (Workplace Interventions preventing Risky alcohol Use and Sick leave). This study was designed by MMT and RWA. MMT analysed the data and drafted the manuscript. JCS, TB, LSS, RWA and MMT provided scientific input to the different drafts and provided data interpretation. All authors made critical revisions and provided intellectual content to the manuscript, approved the final version to be published, and agreed to be accountable for all aspects of this work.

Funding

This work was supported by the Norwegian Directorate of Health (grant number: n/a) and the Research Council of Norway (grant number: 260640).

For peer review only

Competing interests

None declared.

Data availability statement

De-identified data from the WIRUS Screening study are available from the corresponding author on reasonable request.

SUPPLEMENTARY FILE

Supplementary File: Correlation analyses and study selection analyses (PDF)

REFERENCES

1 Griswold MG, Fullman N, Hawley C, et al. Alcohol use and burden for 195 countries and territories, 1990–2016: a systematic analysis for the Global Burden of Disease Study 2016. *Lancet* 2018;392:1015-35. doi: 10.1016/s0140-6736(18)31310-2

2 Rehm J, Gmel GE, Gmel G, et al. The relationship between different dimensions of alcohol use and the burden of disease - an update. *Addiction* 2017;112:968-1001. doi: 10.1111/add.13757

3 Room R, Babor T, Rehm J. Alcohol and public health. *Lancet* 2005;365:519-30. doi: 10.1016/s0140-6736(05)70276-2

4 Wood AM, Kaptoge S, Butterworth AS, et al. Risk thresholds for alcohol consumption: combined analysis of individual-participant data for 599 912 current drinkers in 83 prospective studies. *Lancet* 2018;391:1513-23. doi: 10.1016/S0140-6736(18)30134-X

5 World Health Organization. Global status report on alcohol and health 2018. Geneva, Switzerland: World Health Organization 2018.

6 Vos T, Lim SS, Abbafati C, et al. Global burden of 369 diseases and injuries in 204 countries and territories, 1990–2019: a systematic analysis for the Global Burden of Disease Study 2019. *Lancet* 2020;396:1204-22.

7 Babor TF, Higgins-Biddle JC, Saunders JB, et al. AUDIT: The alcohol use disorders identification test. Guidelines for use in primary health care. 2nd ed. Geneva, Switzerland: World Health Organization 2001.

8 Collin J, Casswell S. Alcohol and the sustainable development goals. *Lancet* 2016;387:2582-83. doi: 10.1016/S0140-6736(16)30827-3

9 Frone MR. Alcohol and illicit drug use in the workforce and workplace. Washington, DC: American Psychological Association 2013.

For peer review only

10 Thørrisen MM, Skogen JC, Aas RW. The associations between employees' risky drinking and sociodemographics, and implications for intervention needs. *BMC Public Health* 2018;18 doi: 10.1186/s12889-018-5660-x

11 Amiri S, Behnezhad S. Alcohol consumption and sick leave: a meta-analysis. *J Addict Dis* 2020;38:100-12. doi: 10.1080/10550887.2020.1724606

12 Hashemi N, Skogen JC, Sevic A, et al. A systematic review and meta-analysis uncovering the relationship between alcohol consumption and sickness absence. When type of design, data, and sickness absence make a difference. *PLoS ONE* 2022;17 doi: 10.1371/journal.pone.0262458

13 Marzan M, Callinan S, Livingston M, et al. Systematic review and dose-response meta-analysis on the relationship between alcohol consumption and sickness absence. *Alcohol Alcohol* 2021 doi: 10.1093/alcalc/agab008

14 Schou L, Moan IS. Alcohol use–sickness absence association and the moderating role of gender and socioeconomic status: A literature review. *Drug Alcohol Rev* 2016;35:158-69. doi: 10.1111/dar.12278

15 Ford MT, Cerasoli CP, Higgins JA, et al. Relationships between psychological, physical, and behavioural health and work performance: A review and meta-analysis. *Work & Stress* 2011;25:185-204. doi: 10.1080/02678373.2011.609035

16 Thørrisen MM, Bonsaksen T, Hashemi N, et al. Association between alcohol consumption and impaired work performance (presenteeism): a systematic review. *BMJ Open* 2019;9 doi: 10.1136/bmjopen-2019-029184

17 Kaner EF, Beyer FR, Muirhead C, et al. Effectiveness of brief alcohol interventions in primary care populations. *Cochrane Database Syst Rev* 2018;2:CD004148. doi: 10.1002/14651858.CD004148.pub4

18 O'Donnell P, Anderson P, Newbury-Birch D, et al. The impact of brief alcohol interventions in primary healthcare: A systematic review of reviews. *Alcohol Alcohol* 2014;49:66-78. doi: 10.1093/alcalc/agt170

19 Schulte B, O'Donnell AJ, Kastner S, et al. Alcohol screening and brief intervention in workplace settings and social services: A comparison of literature. *Front Psychiatry* 2014;5 doi: 10.3389/fpsyt.2014.00131

20 Webb G, Shakeshaft A, Sanson-Fisher R, et al. A systematic review of work-place interventions for alcohol-related problems. *Addiction* 2009;104:365-77. doi: 10.1111/j.1360-0443.2008.02472.x

21 Yuvaraj K, Eliyas SK, Gokul S, et al. Effectiveness of workplace intervention for reducing alcohol consumption: a systematic review and meta-analysis. *Alcohol Alcohol* 2019;54:264-71. doi: 10.1093/alcalc/agz024

22 Frone MR. Predictors of overall and on-the-job substance use among young workers. *J Occup Health Psychol* 2003;8:39-54. doi: 10.1037/1076-8998.8.1.39

23 Marchand A, Parent-Lamarche A, Blanc ME. Work and high-risk alcohol consumption in the Canadian workforce. *Int J Environ Res Public Health* 2011;8:2692-705. doi: 10.3390/ijerph8072692

24 Skogen JC, Bøe T, Thørrisen MM, et al. Sociodemographic characteristics associated with alcohol consumption and alcohol-related consequences, a latent class analysis of the

For peer review only

For peer review only - http://bmjopen.bmj.com/site/about/guidelines.xhtml

Norwegian WIRUS screening study. *BMC Public Health* 2019;19 doi: 10.1186/s12889-019-7648-6

25 Ames GM, Grube JW, Moore RS. Social control and workplace drinking norms: A comparison of two organizational cultures. *J Stud Alcohol* 2000;61:203-19. doi: 10.15288/jsa.2000.61.203

26 Barrientos-Gutierrez T, Gimeno D, Mangione TW, et al. Drinking social norms and drinking behaviours: a multilevel analysis of 137 workgroups in 16 worksites. *Occup Environ Med* 2007;64:602-08. doi: 10.1136/oem.2006.031765

27 Hodgins DC, Williams R, Munro G. Workplace responsibility, stress, alcohol availability and norms as predictors of alcohol consumption-related problems among employed workers. *Subst Use Misuse* 2009;44:2062-69. doi: 10.3109/10826080902855173

28 Frone MR, Bamberger PA. Alcohol and illicit drug involvement in the workforce and workplace. In: Quick J, Fisher G, Ford M, Tetrick L, eds. Handbook of occupational health psychology. Washington, DC: American Psychological Association In press 2022: chapter 18.

29 Pachito DV, Pega F, Bakusic J, et al. The effect of exposure to long working hours on alcohol consumption, risky drinking and alcohol use disorder: A systematic review and meta-analysis from the WHO/ILO Joint Estimates of the Work-related Burden of Disease and Injury. *Environ Int* 2021;146 doi: 10.1016/j.envint.2020.106205

30 Virtanen M, Jokela M, Nyberg ST, et al. Long working hours and alcohol use: systematic review and meta-analysis of published studies and unpublished individual participant data. *BMJ* 2015;350 doi: 10.1136/bmj.g7772

31 Frone MR. Prevalence and distribution of alcohol use and impairment in the workplace: a U.S. national survey. *J Stud Alcohol* 2006;67:147-56. doi: 10.15288/jsa.2006.67.147

32 Marchand A. Alcohol use and misuse: What are the contributions of occupation and work organization conditions? *BMC Public Health* 2008;8 doi: 10.1186/1471-2458-8-333

33 Saade SL, Marchand A. Work organization conditions, alcohol misuse: The moderating role of personality traits. *Work* 2013;44:191-200. doi: 10.3233/WOR-2012-1408

34 Cheng WJ, Cheng Y, Huang MC, et al. Alcohol dependence, consumption of alcoholic energy drinks and associated work characteristics in the Taiwan working population. *Alcohol Alcohol* 2012;47:372-79. doi: 10.1093/alcalc/ags034

35 Moan IS, Halkjelsvik T. Alkohol og arbeidsliv. En undersøkelse blant norske arbeidstakere [Alcohol and work. A survey among Norwegian employees]. Oslo, Norway: Norwegian Institute of Public Health 2016.

36 Plescia F, Cirrincione L, Martorana D, et al. Alcohol abuse and insomnia disorder: Focus on a group of night and day workers. *Int J Environ Res Public Health* 2021;18 doi: 10.3390/ijerph182413196

37 Buchvold HV, Pallesen S, Øyane NMF, et al. Associations between night work and BMI, alcohol, smoking, caffeine and exercise - a cross-sectional study. *BMC Public Health* 2015;15 doi: 10.1186/s12889-015-2470-2

38 Ansoleaga E, Montano R, Vezina M. Psychosocial risk at work and hazardous alcohol consumption among Chile's working adults. *Can J Public Health* 2013;104:502-08. doi: 10.17269/cjph.104.4045

For peer review only - http://bmjopen.bmj.com/site/about/guidelines.xhtml

39 Hermansson U, Knutsson A, Brandt L, et al. Screening for high-risk and elevated alcohol consumption in day and shift workers by use of the AUDIT and CDT. *Occup Med* 2003;53 doi: 10.1093/occmed/kqg104

40 Nielsen MB, Christensen JO, Knardahl S. Working at home and alcohol use. *Addict Behav Rep* 2021;14 doi: 10.1016/j.abrep.2021.100377

41 Lee CM, Cadigan JM, Fairlie AM, et al. Transitions into young adulthood: Extent to which alcohol use, perceived drinking norms, and consequences vary by education and work statuses among 18-20 year olds. *Addict Behav* 2018;79:107-12. doi: 10.1016/j.addbeh.2017.12.004

42 Bloomfield K, Grittner U, Kramer S, et al. Social inequalities in alcohol consumption and alcohol-related problems in the study countries of the EU concerted action 'gender, culture and alcohol problems: a multi-national study'. *Alcohol Alcohol* 2006;41;26-36. doi: 10.1093/alcalc/agl073

43 Probst C, Manthey J, Rehm J. Understanding the prevalence of lifetime abstinence from alcohol: an ecological study. *Drug Alcohol Depen* 2017;178;126-9. doi: 10.1016/j.drugalcdep.2017.05.008

44 Kan MY, Lan M. Comparing alcohol affordability in 65 cities worldwide. *Drug Alcohol Rev* 2013;32:19-26. doi: 10.1111/j.1465-3362.2012.00476.x

45 Kawakami N, Haratani T, Hemmi T, et al. Prevalence and demographic correlates of alcohol-related problems in Japanese employees. *Soc Psych Psych Epid* 1992;27;198-202. doi: 10.1007/BF00789006

46 Roman PM, Trice HM. The development of deviant drinking behavior. *Arch Environ Health* 1970;20:424-35. doi: 10.1080/00039896.1970.10665615

47 Macdonald S, Wells S, Wild TC. Occupational risk factors associated with alcohol and drug problems. *Am J Drug Alcohol Abuse* 1999;25:351-69. doi: 10.1081/ADA-100101865

48 Bacharach SB, Bamberger PA, Sonnenstuhl WJ. Driven to drink: Managerial control, work-related risk factors, and employee problem drinking. *Acad Manage J* 2002;45:637-58. doi: 10.5465/3069302

49 Nielsen MB, Gjerstad J, Frone MR. Alcohol use and psychosocial stressors in the Norwegian workforce. *Subst Use Misuse* 2018;53:574-84. doi: 10.1080/10826084.2017.1349797

50 Frone MR, Trinidad JR. Testing a general model of employee alcohol use and workplace productivity among U.S. workers. *Alcohol Clin Exp Res* 2012;36:229A.

51 Moore RS, Ames GM, Duke MR, et al. Food service employee alcohol use, hangovers and norms during and after work hours. *Journal of Substance Use* 2012;17 doi: 10.3109/14659891.2011.580414

52 Heikkilä K, Fransson EI, Nyberg ST, et al. Job strain and health-related lifestyle: findings from an individual-participant meta-analysis of 118 000 working adults. *Am J Public Health* 2013;103:2090-97.

53 Roxburgh S. Gender differences in the effect of job stressors on alcohol consumption. *Addict Behav* 1998;23:101-07. doi: 10.1016/S0306-4603(97)00025-7

54 Legleye S, Baumann M, Peretti-Watel P, et al. Gender and age disparities in the associations of occupational factors with alcohol abuse and smoking in the French working population. *Revue d'Epidemiologie et de Sante Publique* 2011;59:223-32. doi: 10.1016/j.respe.2011.02.103

For peer review only - http://bmjopen.bmj.com/site/about/guidelines.xhtml

55 Dobson KG, Ibrahim S, Gilbert-Ouimet M, et al. Association between psychosocial work conditions and latent alcohol consumption trajectories among men and women over a 16-year period in a national Canadian sample. *J Epidemiol Community Health* 2018;72:113-20. doi: 10.1136/jech-2017-209691

56 Skogen JC, Thørrisen MM, Bonsaksen T, et al. Effort-reward imbalance is associated with alcohol-related problems. WIRUS-screening study. *Front Psychol* 2019;10 doi: 10.3389/fpsyg.2019.02079

57 Lips-Wiersma M, Wright S, Dik B. Meaningful work: differences among blue-, pink-, and white-collar occupations. *Career Dev Int* 2016;21;534-51. doi: 10.1108/cdi-04-2016-0052

58 Norway. Working Environment Act. Oslo, Norway; Norwegian Ministry of Labour and Social Affairs 2005.

59 Tabachnick BG, Fidell LS. Using multivariate statistics. 6th ed. Boston, MA: Pearson Education 2013.

60 Stevens J. Applied multivariate statistics for the social sciences. 3rd ed. Mahwah, NJ: Lawrence Erlbaum 1996.

61 Saunders JB, Aasland OG, Babor TF, et al. Development of the Alcohol Use Disorders Identification Test (AUDIT): WHO collaborative project on early detection of persons with harmful alcohol consumption-II. *Addiction* 1993;88:791-804. doi: 10.1111/j.1360-0443.1993.tb02093.x

62 Bush K, Kivlahan DR, McDonell MB, et al. The AUDIT alcohol consumption questions (AUDIT-C). An effective brief screening test for problem drinking. *Arch Intern Med* 1998;158:1789-95. doi: 10.1001/archinte.158.16.1789

63 Karasek R, Brisson C, Kawakami N, et al. The Job Content Questionnaire (JCQ): An instrument for internationally comparative assessments of psychosocial job characteristics. *J Occup Health Psychol* 1998;3:322-55. doi: 10.1037/1076-8998.3.4.322

64 Johnson JV, Hall EM, Theorell T. Combined effects of job strain and social isolation on cardiovascular disease morbidity and mortality in a random sample of the Swedish male working population. *Scand J Work Environ Health* 1989;15:271-79. doi: 10.5271/sjweh.1852

65 Karasek R. Job demands, job decision latitude, and mental strain: implications for job redesign. *Adm Sci Q* 1979;24:285. doi: 10.2307/2392498

66 Aas RW. Workplace based sick leave prevention and return to work. Exploratory studies (doctoral thesis). Stockholm, Sweden: Karolinska Institute (Department of Clinical Neuroscience) 2011.

67 Aas RW, Ellingsen KL, Lindøe P, et al. Leadership qualities in the return to work process: a content analysis. *J Occup Rehabil* 2008;18 doi: 10.1007/s10926-008-9147-y

68 Azen R, Budescu DV. The dominance analysis approach for comparing predictors in multiple regression. *Psychol Methods* 2003;8:129-48. doi: 10.1037/1082-989x.8.2.129

69 Neter J, Wasserman W, Kutner MH. Applied linear regression models. Homewood, IL: Irwin 1989.

70 Hosmer DW, Hosmer T, Le Cessie S, et al. A comparison of goodness-of-fit tests for the logistic regression model. *Stat Med* 1997;16;965-80. doi: 10.1002/(sici)1097-0258(19970515)16:9<965::aid-sim509>3.0.co;2-o

For peer review only - http://bmjopen.bmj.com/site/about/guidelines.xhtml

71 Budescu DV. Dominance analysis: A new approach to the problem of relative importance of predictors in multiple regression. *Psychol Bull* 1993;114:542-51. doi: 10.1037/0033-2909.114.3.542

72 Ames GM, Janes C. A cultural approach to conceptualizing alcohol and the workplace. *Alcohol Health Res World* 1992;16:112-19.

73 Gordon R, Heim D, MacAskill S. Rethinking drinking cultures: A review of drinking cultures and a reconstructed dimensional approach. *Public Health* 2012;126:3-11. doi: 10.1016/j.puhe.2011.09.014

74 Savic M, Room R, Mugavin J, et al. Defining "drinking culture": A critical review of its meaning and connotation in social research on alcohol problems. *Drugs* 2016;23:270-82. doi: 10.3109/09687637.2016.1153602

75 Nesvåg S, Lie T. Rusmiddelbruk blant ansatte i norsk privat arbeidsliv [Drug use among employees in Norwegian private sector]. *Nord Alkohol Nark* 2004;21:91-109.

76 Nordaune K, Skarpaas LS, Sagvaag H, et al. Who initiates and organises situations for work-related alcohol use? The WIRUS culture study. *Scand J Public Health* 2017;45:749-56. doi: 10.1177/1403494817704109

77 Bellis MA, Hughes K, Nicholls J, et al. The alcohol harm paradox: using a national survey to explore how alcohol may disproportionately impact health in deprived individuals. *BMC Public Health* 2016;16. doi: 10.1186/s12889-016-2766-x

78 Kalodner CR, Delucia JL, Ursprung AW. An examination of the tension reduction hypothesis: The relationship between anxiety and alcohol in college students. *Addict Behav* 1989;14;649-54. doi: 10.1016/0306-4603(89)90007-5

79 Nicolaisen H, Bråthen K. Frivillig deltid - en privatsak? [Voluntary part-time work - a private matter?]. Oslo, Norway: Fafo 2012.

80 Moe MSH, Aas RW, Thørrisen MM. Tid brukt på daglige aktiviteter og alkoholbruk - WIRUS screening studien [Time spent on daily activities and alcohol consumption. The WIRUS screening study]. *Ergoterapeuten* 2020;63:76-87.

81 Carr N. Going with the flow: An assessment of the relationship between young people's leisure and holiday behaviour. *Tourism Geographies* 2002;4:115-34. doi: 10.1080/14616680210124891

82 Heacock RM, Capodilupo ER, Czeisler ME, et al. Sleep and alcohol use patterns during federal holidays in the United States. *MedRxiv* 2021 doi: 10.1101/2021.11.04.21264353

83 Finne LB, Christensen JO, Knardahl S. Psychological and social work factors as predictors of mental distress: a prospective study. *PLoS ONE* 2014;9 doi: 10.1371/journal.pone.0102514

84 Sterud T, Johannessen HA, Tynes T. Work-related psychosocial and mechanical risk factors for neck/shoulder pain: a 3-year follow-up study of the general working population in Norway. *Int Arch Occup Environ Health* 2014;87:471-81. doi: 10.1007/s00420-013-0886-5

85 Christensen JO, Nielsen MB, Finne LB, et al. Comprehensive profiles of psychological and social work factors as predictors of site-specific and multi-site pain. *Scand J Work Environ Health* 2018;44:291-302. doi: 10.5271/sjweh.3706

86 Felton BJ, Shinn M. Social integration and social support: Moving "social support" beyond the individual level. *J Community Psychol* 1992;20;103-15. doi: 10.1002/1520-6629(199204)20:2<103::aid-jcop2290200202>3.0.co;2-4

For peer review only

For peer review only - http://bmjopen.bmj.com/site/about/guidelines.xhtml

87 Bacharach SB, Bamberger P, Cohen A, et al. Retirement, social support, and drinking behavior: A cohort analysis of males with a baseline history of problem drinking. *J Drug Issues* 2007;37;525-48. doi: 10.1177/002204260703700303

88 Thørrisen MM. Alcohol consumption and impaired work performance. Interventions, and implementation barriers (doctoral thesis). Oslo, Norway: OsloMet - Oslo Metropolitan University (Faculty of Health Sciences) 2020.

89 Boniface S, Kneale J, Shelton N. Drinking pattern is more strongly associated with under-reporting of alcohol consumption than socio-demographic factors: evidence from a mixed-methods study. *BMC Public Health* 2014;14 doi: 10.1186/1471-2458-14-1297

90 de Meneses-Gaya C, Zuardi AW, Loureiro SR, et al. Alcohol Use Disorders Identification Test (AUDIT): An updated systematic review of psychometric properties. *Psychol Neurosci* 2009;2:83-97. doi: 10.3922/j.psns.2009.1.12

91 Skogen JC, Thørrisen MM, Olsen E, et al. Evidence for essential unidimensionality of AUDIT and measurement invariance across gender, age and education. Results from the WIRUS study. *Drug Alcohol Depend* 2019;202:87-92. doi: 10.1016/j.drugalcdep.2019.06.002

92 Aalto M, Tuunanen M, Sillanaukee P, et al. Effectiveness of structured questionnaires for screening heavy drinking in middle-aged women. *Alcohol Clin Exp Res* 2006;30:1884-88. doi: 10.1111/j.1530-0277.2006.00233.x

93 Giang KB, Spak F, Dzung TV, et al. The use of AUDIT to assess level of alcohol problems in rural Vietnam. *Alcohol Alcohol* 2005;40:578-83. doi: 10.1093/alcalc/agh198

94 Selin KH. Test-retest reliability of the Alcohol Use Disorder Identification Test in a general population sample. *Alcohol Clin Exp Res* 2003;27:1428-35. doi: 10.1097/01.alc.0000085633.23230.4a

95 Tuunanen M, Aalto M, Seppä K. Binge drinking and its detection among middle-aged men using AUDIT, AUDIT-C and AUDIT-3. *Drug Alcohol Rev* 2007;26:295-99. doi: 10.1080/09595230701247756

96 Lindberg P, Vingård E. Indicators of healthy work environments - a systematic review. *Work* 2012;41:3032-38. doi: 10.3233/wor-2012-0560-3032

97 Nielsen K, Nielsen MB, Ogbonnaya C, et al. Workplace resources to improve both employee well-being and performance: a systematic review and meta-analysis. *Work Stress* 2017;31:101-20. doi: 10.1080/02678373.2017.1304463

98 Burton J. WHO healthy workplace framework and model: background and supporting literature and practice. Geneva, Switzerland: World Health Organization 2010.

FIGURE TITLE/LEGEND

Figure 1. Overview of main study results. Percentages reflect relative importance of predictors based on dominance analysis (average contribution of the predictor to the outcome

For peer review only - http://bmjopen.bmj.com/site/about/guidelines.xhtml

in percentage points when analysed in conjunction with other workplace factors that are significantly associated with the outcome)

For peer review only

For peer review only - http://bmjopen.bmj.com/site/about/guidelines.xhtml

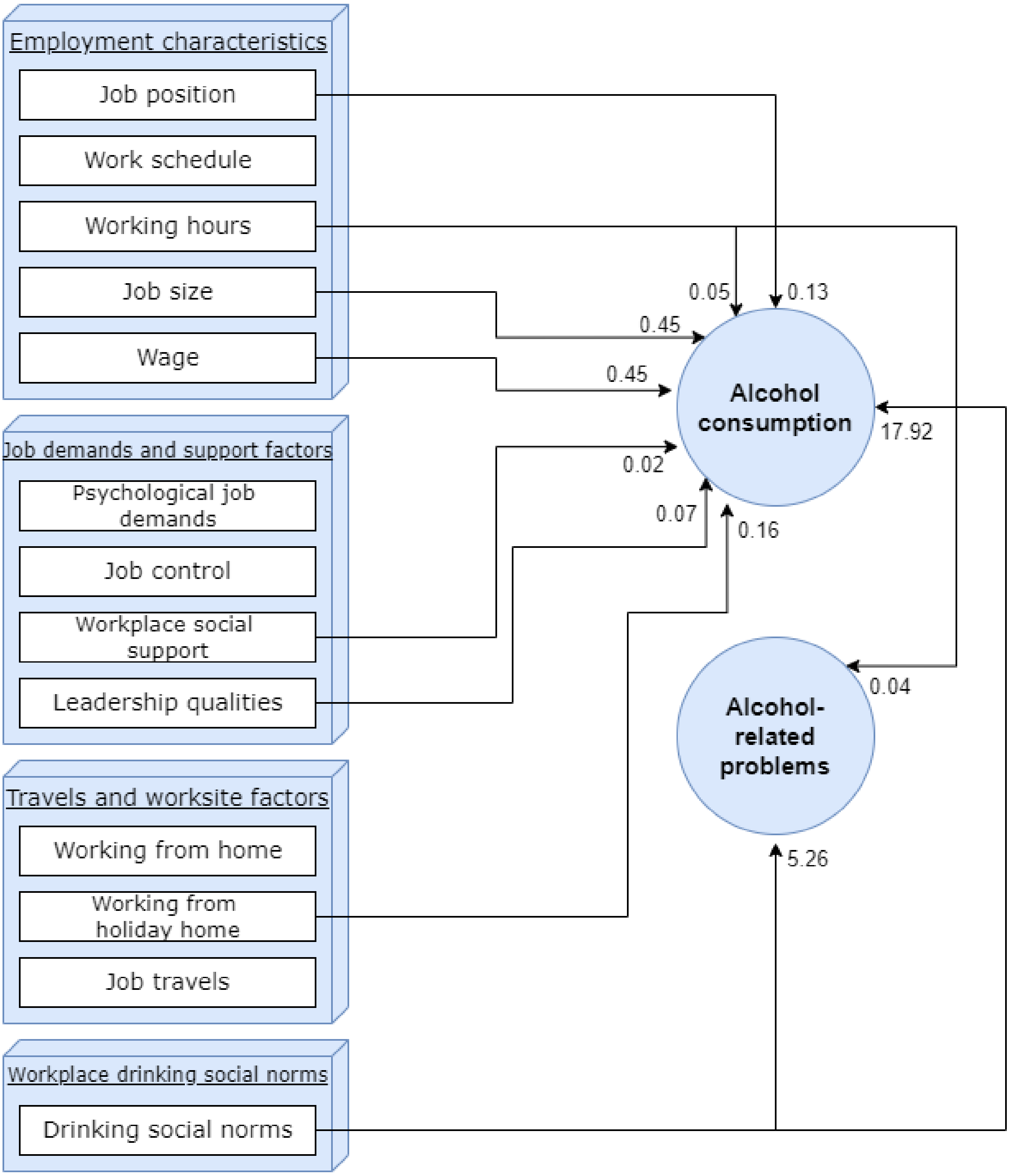
Employment characteristics
Job position
Work schedule
Working hours
Job size
Wage
Job demands and support factors
Psychological job demands
Job control
Workplace social support
Leadership qualities
Travels and worksite factors
Working from home
Working from holiday home
Job travels
Workplace drinking social norms
Drinking social norms
Alcohol consumption
Alcohol-related problems
0.05
0.13
0.45
0.45
0.02
0.07
0.16
17.92
0.04
5.26

Supplementary File

Table S1. Matrix of bivariate correlations between all study variables (outcomes, predictors and covariates)

	1	2	3	4	5	6	7	8	9	10	11	12	13	14	15	16	17	18	19	20	21	22	23	24	25
1. Alcohol consumption																									
2. Alcohol-rel. problems[A]	**.49**																								
3. Working hours	.02	-.01																							
4. Day job, no weekends[B]	**-.05**	**-.05**	.02																						
5. Evening job[B]	.01	.02	**-.05**	**-.11**																					
6. Night job[B]	-.01	.01	-.01	**-.21**	-.01																				
7. Shift work[B]	**.04**	**.04**	**-.08**	**-.79**	**-.03**	**-.06**																			
8. Job position[C]	**.04**	**-.03**	**.24**	**.12**	**-.03**	**-.06**	**-.14**																		
9. Job size[D]	**.08**	**.04**	**.25**	**.20**	**-.10**	**-.25**	**-.15**	**.19**																	
10. Income	.01	**-.08**	**.14**	**.13**	**-.04**	**-.05**	**-.13**	**.21**	**.11**																
11. Psych. job demands	-.02	-.00	**.23**	**-.05**	-.00	**-.06**	.03	**.17**	**.07**	**.05**															
12. Job control	-.01	**-.04**	**.15**	**.10**	-.01	**-.08**	**-.12**	**.14**	**.08**	**.17**	**.14**														
13. Work. social support	.01	**-.03**	.00	**.06**	-.01	**-.03**	**-.05**	.00	-.00	**.05**	**-.06**	**.34**													
14. Leadership qualities	-.02	**-.03**	-.02	.02	-.01	**-.04**	-.01	-.01	-.01	.01	**-.10**	**.23**	**.61**												
15. Job travels[E]	**.07**	.02	**.13**	**.09**	-.02	**-.05**	**-.11**	**.16**	**.13**	**.15**	**-05**	**.14**	.01	-.01											
16. Working from home[E]	.02	-.00	**.12**	**.04**	-.01	**-.03**	**-.09**	**.11**	**.04**	**.10**	**.06**	**.13**	.01	-.01	**.25**										
17. Work. holiday home[E]	**.06**	.01	**.12**	-.03	-.01	-.02	-.02	**.10**	**.05**	**.11**	**.07**	**.06**	-.02	-.02	**.24**	**.32**									
18. Drinking social norms[F]	**.42**	**.23**	**.06**	**-.05**	.02	-.03	**.04**	**-.05**	**.07**	.02	-.01	-.01	.00	.01	**.09**	**.06**	**.03**								
19. Gender[G]	**-.18**	**-.14**	**-.16**	**.04**	.01	.02	-.02	**-.13**	**-.17**	-.02	**.05**	.01	**.06**	**.05**	**-.17**	**-.03**	**-.08**	**-.16**							
20. Age	**-.12**	**-.13**	**.05**	**.19**	-.01	-.01	**-.20**	**.18**	.00	**.10**	**-.07**	-.02	**-.09**	**-.10**	.03	-.02	**.05**	**-.25**	**-.04**						
21. Educational attainment	**-.03**	-.03	**.18**	**.15**	-.03	**-.07**	**-.18**	**.09**	**.13**	**.29**	**.10**	**.30**	**.08**	**.04**	**.16**	**.14**	**.06**	**.06**	.01	**-.07**					
22. Living status[H]	**-.07**	**-.12**	.02	**.07**	-.01	-.02	**-.06**	**.06**	-.02	**.50**	.01	**.07**	**.03**	.03	**.03**	.01	**.03**	**-.04**	-.01	-.02	**.03**				
23. Marital status[I]	**-.14**	**-.15**	**.04**	**.14**	-.01	-.02	**-.15**	**.13**	-.02	**.46**	.00	**.08**	.01	-.00	**.06**	**.03**	**.04**	**-.12**	**-.04**	**.26**	**.07**	**.46**			
24. Number of children	**-.16**	**-.17**	**.03**	**.15**	-.02	-.01	**-.15**	**.14**	-.02	**.31**	-.01	**.07**	-.01	-.01	**.06**	**.04**	**.04**	**-.20**	.00	**.40**	.01	**.33**	**.42**		
25. Year of data collection	.01	.01	**-.10**	**-.12**	**.04**	**.04**	**.16**	.00	**-.04**	**-.07**	.02	**.06**	**.07**	**.09**	**-.15**	**-.06**	-.01	**-.04**	**.14**	**-.07**	**.04**	-.02	**-.03**	-.02	

N = 5388; Results from non-parametric (Spearman *rho*) correlation analyses; Statistically significant correlations (p <.05) indicated with bold typeface; [A]Ref. = no problems; [B]Ref. = other work schedules; [C]Ref. = worker; [D]Ref. = part-time; [E]Ref. = no; [F]Higher scores indicate liberal attitudes; [G]Ref. = male; [H]Ref. = living alone; [I]Ref. = unmarried; For all other variables: higher scores indicate higher variable values

For peer review only

Table S2. Study selection analyses: Comparisons between completers and non-completers on sociodemographic variables

Variable	Completers[A]	Non-completers[B]	*p*
Gender, % females	68.5	75.7	<.001[C]
Age, *M*	44.8	44.7	.946[D]
Education, % university/college	77.7	66.2	<.001[C]
Livings status, % living alone	15.1	15.1	.949[C]
Marital status, % unmarried	44.8	48.0	.005[C]
Children, *M*	1.8	1.7	.001[D]

[A]Employees who responded on all study items, i.e., the study sample (n = 5388); [B]Employees who responded on the sociodemographic items but not on all study items; [C]Difference tested with chi square tests of independence; [D]Difference tested with independent samples t-tests

For peer review only

STROBE Statement

Checklist of items that should be included in reports of observational studies

Section/Topic	Item No	Recommendation	Reported on Page No
**Title and abstract**	1	(*a*) Indicate the study's design with a commonly used term in the title or the abstract	1,2
		(*b*) Provide in the abstract an informative and balanced summary of what was done and what was found	2,3
**Introduction**			
Background/rationale	2	Explain the scientific background and rationale for the investigation being reported	3-8
Objectives	3	State specific objectives, including any prespecified hypotheses	8
**Methods**			
Study design	4	Present key elements of study design early in the paper	8
Setting	5	Describe the setting, locations, and relevant dates, including periods of recruitment, exposure, follow-up, and data collection	8-10
Participants	6	(*a*) *Cohort study*—Give the eligibility criteria, and the sources and methods of selection of participants. Describe methods of follow-up<br>*Case-control study*—Give the eligibility criteria, and the sources and methods of case ascertainment and control selection. Give the rationale for the choice of cases and controls<br>*Cross-sectional study*—Give the eligibility criteria, and the sources and methods of selection of participants	8-10
		(*b*) *Cohort study*—For matched studies, give matching criteria and number of exposed and unexposed<br>*Case-control study*—For matched studies, give matching criteria and the number of controls per case	
Variables	7	Clearly define all outcomes, exposures, predictors, potential confounders, and effect modifiers. Give diagnostic criteria, if applicable	10-13
Data sources/measurement	8*	For each variable of interest, give sources of data and details of methods of assessment (measurement). Describe comparability of assessment methods if there is more than one group	10-13
Bias	9	Describe any efforts to address potential sources of bias	12,13
Study size	10	Explain how the study size was arrived at	10
Quantitative variables	11	Explain how quantitative variables were handled in the analyses. If applicable, describe which groupings were chosen and why	10-13
Statistical methods	12	(*a*) Describe all statistical methods, including those used to control for confounding	13,14
		(*b*) Describe any methods used to examine subgroups and interactions	n/a
		(*c*) Explain how missing data were addressed	Suppl.
		(*d*) *Cohort study*—If applicable, explain how loss to follow-up was addressed<br>*Case-control study*—If applicable, explain how matching of cases and controls was addressed<br>*Cross-sectional study*—If applicable, describe analytical methods taking account of sampling strategy	n/a
		(*e*) Describe any sensitivity analyses	Suppl.

For peer review only - http://bmjopen.bmj.com/site/about/guidelines.xhtml

Section/Topic	Item No	Recommendation	Reported on Page No
**Results**			
Participants	13*	(a) Report numbers of individuals at each stage of study—eg numbers potentially eligible, examined for eligibility, confirmed eligible, included in the study, completing follow-up, and analysed	9
		(b) Give reasons for non-participation at each stage	n/a
		(c) Consider use of a flow diagram	n/a
Descriptive data	14*	(a) Give characteristics of study participants (eg demographic, clinical, social) and information on exposures and potential confounders	9,10
		(b) Indicate number of participants with missing data for each variable of interest	n/a
		(c) *Cohort study*—Summarise follow-up time (eg, average and total amount)	
Outcome data	15*	*Cohort study*—Report numbers of outcome events or summary measures over time	
		Case-control study—Report numbers in each exposure category, or summary measures of exposure	
		Cross-sectional study—Report numbers of outcome events or summary measures	10
Main results	16	(*a*) Give unadjusted estimates and, if applicable, confounder-adjusted estimates and their precision (eg, 95% confidence interval). Make clear which confounders were adjusted for and why they were included	14-22
		(*b*) Report category boundaries when continuous variables were categorized	n/a
		(*c*) If relevant, consider translating estimates of relative risk into absolute risk for a meaningful time period	n/a
Other analyses	17	Report other analyses done—eg analyses of subgroups and interactions, and sensitivity analyses	Suppl.
**Discussion**			
Key results	18	Summarise key results with reference to study objectives	23
Limitations	19	Discuss limitations of the study, taking into account sources of potential bias or imprecision. Discuss both direction and magnitude of any potential bias	27-29
Interpretation	20	Give a cautious overall interpretation of results considering objectives, limitations, multiplicity of analyses, results from similar studies, and other relevant evidence	23-27
Generalisability	21	Discuss the generalisability (external validity) of the study results	27,28
**Other Information**			
Funding	22	Give the source of funding and the role of the funders for the present study and, if applicable, for the original study on which the present article is based	31

**Give information separately for cases and controls in case-control studies and, if applicable, for exposed and unexposed groups in cohort and cross-sectional studies.*

Note: An Explanation and Elaboration article discusses each checklist item and gives methodological background and published examples of transparent reporting. The STROBE checklist is best used in conjunction with this article (freely available on the Web sites of PLoS Medicine at http://www.plosmedicine.org/, Annals of Internal Medicine at http://www.annals.org/, and Epidemiology at http://www.epidem.com/). Information on the STROBE Initiative is available at www.strobe-statement.org.

For peer review only - http://bmjopen.bmj.com/site/about/guidelines.xhtml

BMJ Open

Are workplace factors associated with employee alcohol use? The WIRUS cross-sectional study

Journal:	*BMJ Open*
Manuscript ID	bmjopen-2022-064352.R2
Article Type:	Original research
Date Submitted by the Author:	01-Sep-2022
Complete List of Authors:	Thørrisen, Mikkel; Oslo Metropolitan University, Department of Rehabilitation Science and Health Technology, Faculty of Health Sciences; University of Stavanger, Department of Public Health, Faculty of Health Sciences<br>Skogen, Jens; Norwegian Institute of Public Health, Department of Health Promotion; Stavanger University Hospital, Center for Alcohol & Drug Research<br>Bonsaksen, Tore; Inland Norway University of Applied Sciences, Department of Health and Nursing Sciences, Faculty of Social and Health Sciences; VID Specialized University, Department of Health, Faculty of Health Studies<br>Skarpaas, Lisebet; Oslo Metropolitan University, Department of Rehabilitation Science and Health Technology, Faculty of Health Sciences<br>Aas, Randi; Oslo Metropolitan University, Department of Rehabilitation Science and Health Technology, Faculty of Health Sciences; University of Stavanger, Department of Public Health, Faculty of Health Sciences
**Primary Subject Heading**:	Public health
Secondary Subject Heading:	Occupational and environmental medicine
Keywords:	OCCUPATIONAL & INDUSTRIAL MEDICINE, PUBLIC HEALTH, EPIDEMIOLOGY

SCHOLARONE™ Manuscripts

I, the Submitting Author has the right to grant and does grant on behalf of all authors of the Work (as defined in the below author licence), an exclusive licence and/or a non-exclusive licence for contributions from authors who are: i) UK Crown employees; ii) where BMJ has agreed a CC-BY licence shall apply, and/or iii) in accordance with the terms applicable for US Federal Government officers or employees acting as part of their official duties; on a worldwide, perpetual, irrevocable, royalty-free basis to BMJ Publishing Group Ltd ("BMJ") its licensees and where the relevant Journal is co-owned by BMJ to the co-owners of the Journal, to publish the Work in this journal and any other BMJ products and to exploit all rights, as set out in our licence.

The Submitting Author accepts and understands that any supply made under these terms is made by BMJ to the Submitting Author unless you are acting as an employee on behalf of your employer or a postgraduate student of an affiliated institution which is paying any applicable article publishing charge ("APC") for Open Access articles. Where the Submitting Author wishes to make the Work available on an Open Access basis (and intends to pay the relevant APC), the terms of reuse of such Open Access shall be governed by a Creative Commons licence – details of these licences and which Creative Commons licence will apply to this Work are set out in our licence referred to above.

Other than as permitted in any relevant BMJ Author's Self Archiving Policies, I confirm this Work has not been accepted for publication elsewhere, is not being considered for publication elsewhere and does not duplicate material already published. I confirm all authors consent to publication of this Work and authorise the granting of this licence.

For peer review only

Are workplace factors associated with employee alcohol use? The WIRUS cross-sectional study

Mikkel Magnus Thørrisen[1,2]*
Jens Christoffer Skogen[2,3,4]
Tore Bonsaksen[5,6]
Lisebet Skeie Skarpaas[1]
Randi Wågø Aas[1,2]*

[1]Department of Rehabilitation Science and Health Technology, Faculty of Health Sciences, OsloMet – Oslo Metropolitan University, Oslo, Norway

[2]Department of Public Health, Faculty of Health Sciences, University of Stavanger, Stavanger, Norway

[3]Department of Health Promotion, Norwegian Institute of Public Health, Bergen, Norway

[4]Center for Alcohol & Drug Research, Stavanger University Hospital, Stavanger, Norway

[5]Department of Health and Nursing Sciences, Faculty of Social and Health Sciences, Inland Norway University of Applied Sciences, Elverum, Norway

[6]Department of Health, Faculty of Health Studies, VID Specialized University, Stavanger, Norway

*Correspondence:
Mikkel Magnus Thørrisen / Randi Wågø Aas

OsloMet – Oslo Metropolitan University, Faculty of Health Sciences, Department of Rehabilitation Science and Health Technology, PO box 4 St. Olavs plass, NO-0130 Oslo, Norway

mikkel-magnus.thorrisen@oslomet.no / randi.aas@oslomet.no

Word count: 6251 (main text, i.e., excluding title page, abstract, strength/limitations statement, declarations, reference list, tables, figure and supplementary material)

For peer review only

For peer review only - http://bmjopen.bmj.com/site/about/guidelines.xhtml

ABSTRACT

Objectives: Sociodemographic predictors of employee alcohol use are well established in the literature, but knowledge about associations between workplace factors and alcohol use is less explored. The aim of this study was to explore whether workplace factors were associated with employee alcohol use (consumption and alcohol-related problems).

Design: Cross-sectional study. Linear and binary logistic regression analyses.

Setting: Heterogeneous sample of employees (workers and supervisors) from 22 companies across geographical locations and work divisions in Norway.

Participants: Employees (N = 5388) responded on survey items measuring workplace factors and alcohol use.

Outcomes: Data on alcohol use were collected with the Alcohol Use Disorders Identification Test (AUDIT). Consumption was measured with the AUDIT-C (the first three items), and alcohol-related problems were operationalised as a sum score of eight or higher on the full ten-item AUDIT.

Results: Higher levels of alcohol consumption were associated with more liberal workplace drinking social norms (b = 1.37, p <.001), working full-time (b = .18, p <.001), working from holiday home (b = .40, p <.01), being a supervisor (b = .25, p <.001), having supervisors with less desired leadership qualities (b = -.10, p <.01), shorter working hours (b = -.03, p <.05), higher workplace social support (b = .13, p <.05), and higher income (b = .02, p <.001). Alcohol-related problems were associated with more liberal workplace drinking social norms (OR = 3.52, p <.001) and shorter working hours (OR = 0.94, p <.05).

Conclusions: Workplace drinking social norms was the supremely most dominant predictor of both consumption and alcohol-related problems. Results suggest that some workplace factors may play a role in explaining employee alcohol consumption, although the predictive

For peer review only - http://bmjopen.bmj.com/site/about/guidelines.xhtml

ability of these factors was limited. This study points to the importance of drinking social norms, workplace drinking culture and leadership for understanding employee alcohol use.

Keywords: Alcohol Drinking; Employees; Work; Workplace

Strengths and limitations of this study

- This study explores the importance of a wide range of workplace factors for employee alcohol use in a large and heterogeneous sample by means of a validated alcohol screening instrument
- This study utilises individuals' overall degree of consumption, as well as odds of having a drinking pattern that may induce alcohol-related problems, as outcomes
- Study limitations include a low response rate, the use of self-reported data, and a cross-sectional design that precludes causal inferences

INTRODUCTION

Alcohol consumption is associated with detrimental health outcomes.[1-4] Globally, approximately five percent of mortality and disability-adjusted life years (DALYs) can be attributed to alcohol consumption.[5] Within the age group of 25-49 years, 1.6 % of global DALYs were attributable to alcohol use disorders in 2019.[6] Risky drinking has been conceptualised as a drinking pattern that increases the risk of social, legal, medical, occupational, domestic and economic problems.[7] Reducing harmful alcohol use represents a keystone in sustainable development of health.[5, 8]

Alcohol is the most commonly used and misused psychoactive substance in the workforce.[9] Between 10 and 30 % of employees consume alcohol at a risky level and may benefit from alcohol prevention interventions.[10] Employees’ alcohol use is associated with

For peer review only - http://bmjopen.bmj.com/site/about/guidelines.xhtml

For peer review only

increased sickness absence,[11-14] and presenteeism (impaired on-the-job performance).[15, 16] Research has demonstrated that brief alcohol interventions have promising effects in primary care,[17, 18] as well as in working populations.[19-21]

According to Frone’s model of employee substance use,[22] employee alcohol use can be construed as a function of individual and workplace environment characteristics, and multifaceted interactions between them. Individual (sociodemographic) predictors of alcohol use among employees are quite well established in the research literature. For instance, being male, young, unmarried and not having children constitute individual predictors of elevated alcohol consumption.[10, 23, 24] The role of workplace factors is, however, not as established in the literature. Workplace factors may, on the one hand, refer to employment characteristics, i.e., to more general workplace factors conceptualised in the form of conditions and terms defining the work situation (e.g., working hours, work schedule, job position, job size and income). On the other hand, the workplace can be understood as a psycho-socio-cultural setting that involves job demands and support factors (which are related to occupational stress, e.g., psychological job demands, job control, workplace social support and supervisors' perceived leadership qualities), travels and worksite factors (related to degree of workplace social control, e.g., job travels, working from home and working from holiday home), and workplace drinking social norms (which reflect the workplace alcohol use climate, e.g., workplace drinking culture and drinking social norms).

Studies have demonstrated that workplace alcohol use climate may affect the drinking level of employees,[25-27] e.g., through the impact of injunctive and descriptive norms.[28] "Drinking social norms shared by a group define standards of appropriate behaviour, creating social controls that regulate workplace alcohol availability and drinking behaviours".[26, p. 602] For instance, in a large-scale study of employees in the USA (across workgroups and worksites) it was found that being part of an alcohol discouraging workplace drinking culture

For peer review only - http://bmjopen.bmj.com/site/about/guidelines.xhtml

predicted lower consumption (less frequent drinking in general and at work, as well as overall reduced likelihood of being a heavy drinker), compared to being part of a more encouraging workplace drinking culture.[26] Using multilevel analysis, it has been found that individual-level measurement of drinking social norms may be indicative of group-level norms at worksites employees are nested within.[26] Research on the importance of other aspects of the workplace and work situation, such as general employment characteristics, job demands and support factors and travels and worksite factors, stands out as more inconclusive.

Systematic reviews generally point to longer working hours being associated with higher alcohol consumption.[29, 30] However, the evidence for such a relationship remains uncertain. Some studies have failed to demonstrate statistically significant relationships,[31-33] and an opposite association (shorter working hours associated with higher risk of alcohol dependence) was found in a study of employees in Taiwan.[34] In a Norwegian study,[35] having flexible working hours was associated with increased consumption, and some studies have linked shift work with increased probability of workplace alcohol use,[31] as well as with higher overall consumption.[36] Others have not found a relationship between shift work and alcohol use.[35, 37-39] Evidence on the importance of work position is mixed. Some studies support an association between being a supervisor (as opposed to a worker) and higher alcohol consumption,[32, 40] while others do not.[23, 31, 34, 35] In a study of employees in the USA, Lee et al. revealed that working part-time (as opposed to full-time) was associated with higher alcohol use per week, but unrelated to prevalence of binge drinking episodes and alcohol-related consequences.[41] Although working hours may be somewhat overlapping with job size, one may capture different aspects of "time at work" by studying typical number of hours at work per day (working hours) and whether or not an individual has a full-time job (job-size). For instance, variations in working hours that exceed "normal working hours" (e.g., overtime) will not be captured in a dichotomy of part-time and full-time work. Studies have

For peer review only - http://bmjopen.bmj.com/site/about/guidelines.xhtml

suggested that higher socioeconomic status is associated with higher levels of alcohol consumption,[42, 43] which may be due to alcohol being a costly commodity.[44] As such, high-income employees may have better access to alcohol than low-income employees. A study of Canadian employees found that employees in high-income households had 35 % higher odds of high-risk drinking than employees in low-income households.[23] However, in a study of Japanese employees,[45] family income was unrelated to prevalence of alcohol-related problems.

Travels and worksite factors may refer to the degree to which employees are subjects of direct monitoring at the workplace. According to Roman and Trice,[46] low visibility at the workplace and low degrees of interdependence among employees stand out as risk factors for deviant drinking behaviours. In a study of employees in Norway,[40] working from home (telecommuting) at least two days per week was associated with higher alcohol use. Furthermore, a high prevalence of job travels has been linked with increased likelihood of problematic drinking.[47]

Research on the importance of job demands and support factors (e.g., leadership, job demands and control, and workplace social support) for employee alcohol use have yielded variable results. It has been advocated that visible and active supervisors constitute a protective factor for alcohol use among workers,[46] while others have failed to demonstrate such a relationship.[47, 48] In a Norwegian study,[49] supervisors' leadership style (transformational and laissez-faire leadership) was not related to employee alcohol use. On the other hand, some studies imply that alcohol use and hangover episodes are less prevalent at workplaces where supervisors exert active social control.[25, 50, 51] Analyses of cross-sectional data imply that lower levels of job control (skill discretion and decision latitude) may be associated with higher levels of alcohol consumption, although such a relationship has proved difficult to replicate in longitudinal data.[52] Some studies reject a potential

For peer review only - http://bmjopen.bmj.com/site/about/guidelines.xhtml

association between job control and alcohol use,[23, 34, 53] while others underscore that different types of control may be differentially related to consumption. For instance, Marchand found that an increased probability of risky drinking was associated with lower skill discretion and higher decision latitude,[32] while Saade and Marchand found the opposite.[33] Higher perceived psychological job demands have in some studies been related to higher alcohol use,[33, 34] but not in others.[54] In a study of Canadian employees,[55] lower psychological demands were associated with higher alcohol use for men only. The evidence base of physical job demands is also inconclusive. A Canadian study found that higher physical demands were associated with higher alcohol use among men only,[55] while a French study found an association for women only.[54] Another Canadian study failed to demonstrate a relationship between physical job demands and alcohol use altogether.[33] Some studies indicate that lower workplace social support is associated with higher alcohol consumption,[33, 38, 55] while others do not.[23, 53] A study from Norway found increased odds of alcohol-related problems among employees who reported a perceived imbalance between work efforts and rewards, in conjunction with high levels of work overcommitment.[56]

In summary, evidence on associations between workplace factors and employee alcohol use stands out as complex and nuanced. Moreover, few studies have explored the importance of workplace factors for alcohol consumption as well as for odds of alcohol-related problems in heterogeneous employee samples by means of validated alcohol screening instruments. Individuals' overall degree of consumption, and their odds of having a drinking pattern that may induce alcohol-related problems, constitute two different – yet equally important – ways of conceptualising alcohol use that can be differentially associated with workplace factors. Knowledge about associations between workplace factors and employee alcohol use is important as an aid in determining the extent to which employers should make workplace-

For peer review only - http://bmjopen.bmj.com/site/about/guidelines.xhtml

based alcohol health promotion and prevention interventions an overall priority, and for determining which elements should be emphasised in such interventions. Hence, further research is warranted.

Study aim

The aim of this study was to explore whether workplace factors (employment characteristics, job demands and support factors, travels and worksite factors and workplace drinking social norms) were associated with employee alcohol consumption and odds of having of alcohol-related problems in a heterogeneous sample of employees in Norway.

MATERIALS AND METHODS

Design and setting

This cross-sectional study explored associations between workplace factors and alcohol use in a heterogeneous sample of employees (workers and supervisors) in 22 companies in Norway (private companies: n = 8; central government companies: n = 6; local government companies: n = 8), across geographical locations. The study was part of the Norwegian national WIRUS project (Workplace Interventions preventing Risky alcohol Use and Sick leave).

Data collection and sample

The researchers recruited companies through three occupational health service units' client lists in collaboration with the addiction competence environment KoRus Stavanger. Individual-level inclusion criteria were: (i) age 16-72; (ii) status as employee, regardless of sector, work division or geographical location; and (iii) basic understanding of the Norwegian language. Our definition of employees (criteria ii) included white- (supervisors,

For peer review only - http://bmjopen.bmj.com/site/about/guidelines.xhtml

(semi)professional roles, business owners), blue- (labourers and skilled trade roles) and pink-collar employed (hospitality, retail, care and administration roles).[57] Inclusion of employees aged 16 to 72 (criteria i) was chosen because 16 constitutes the age when individuals in Norway have typically completed mandatory primary and secondary school and are eligible for full-time work (or further education), and because employment by Norwegian law may be terminated by the employer at age 72 (even though 67 constitutes the general age of retirement).[58] Although Norwegian legislation forbids retail, serving and supply of alcohol to anyone under the age of 18,[59] alcohol is regularly consumed by adolescents.[60] Hence, it was deemed appropriate to include respondents aged 16 and 17 in the study. The invited sample was, in accordance with the inclusion criteria, heterogeneous, representing different sectors, work divisions and geographical locations. Twenty-two companies agreed to participate and provided e-mail addresses for all their employees (n = 30811). Digital questionnaires measuring workplace factors, alcohol use and sociodemographic variables were distributed to all employees in the 22 companies. Consent to participate was provided by 8542 employees (response rate = 27.7 %). A total of 3154 employees failed to respond on all relevant study items, leaving a final study sample of 5388 employees. Characteristics of the study sample are presented in Table 1.

Table 1. Characteristics of the study sample

Variable	
**Gender**	
Male, n (%)	1699 (31.5)
Female, n (%)	3689 (68.5)
**Age** (years), *M* (*SD*)	44.8 (11.2)
**Educational attainment**	
Primary/lower secondary, n (%)	87 (1.6)
Upper secondary, n (%)	1006 (18.7)
University/college ≤4 years, n (%)	2125 (39.4)
University/college >4 years, n (%)	2170 (40.3)
**Employment sector**	

For peer review only - http://bmjopen.bmj.com/site/about/guidelines.xhtml

Private corporation, n (%)	368 (6.8)
Local government, n (%)	3469 (64-4)
Central government, n (%)	1551 (28-8)
**Work division**	
Transportation/manufacturing, n (%)	285 (5.3)
Public administration/services, n (%)	4299 (79.8)
Health services, n (%)	762 (14.1)
Other services, n (%)	42 (0.8)

N = 5388; *M* = mean; *SD* = standard deviation

Data from participants were collected between October 2015 and October 2019. Hence, each participant was measured once (between 2015 and 2019), and all data were collected prior to the covid-19 pandemic. We predefined that a statistically satisfactory sample size had to conform with the formula N > 50 + (8 x number of predictors/covariates),[61] as well as exceeding a ratio of 15 participants per predictor/covariate variable.[62]

Measures

Employee alcohol use (outcomes)

The outcomes were measured with the Alcohol Use Disorders Identification Test (AUDIT). The AUDIT is an alcohol screening instrument, developed by the World Health Organization (WHO),[7, 63] consisting of 10 items (each scored 0-4). Higher scores on the AUDIT indicate higher consumption and more severe alcohol-related consequences. The first outcome, *alcohol consumption*, was measured with the first three AUDIT items (sum score of three items = continuous scale 0-12), often referred to as the AUDIT-C.[64] The second outcome, *alcohol-related problems*, was measured as a dichotomous categorical variable (0 = no problems; 1 = problems), based on a cut-off of eight on the full ten-item AUDIT (scores 0-7 = no problems; scores 8-40 = problems).[7]

Workplace factors (predictors)

For peer review only - http://bmjopen.bmj.com/site/about/guidelines.xhtml

Employment characteristics included *working hours* (number of hours during a typical day), *work schedule* (day job without weekends; day job with weekends; evening job; night job; shift work), *job position* (worker; supervisor), *job size* (part-time; full-time), and *income* (gross annual household income in Norwegian Kroner (NOK)).

Job demands and support factors included employees' perceptions of *psychological job demands*, *job control*, *workplace social support* and supervisors' *leadership qualities*. Psychological job demands (five items), job control (perceived control over workplace decisions and potential for utilising personal skills in the job; nine items) and workplace social support (from co-workers and supervisors; eight items) were measured with the Job Content Questionnaire (JCQ),[65] based on the Demand Control Support model,[66, 67] each scored on four-point Likert scales where higher scores indicated higher levels of demands, control and support, respectively. Reversed items were recoded and mean scores were calculated (demands: $\alpha = .76$; control: $\alpha = .58$; support: $\alpha = .89$). Supervisors' leadership qualities were measured with the Seven-item Leadership Qualities Questionnaire (7LQQ).[68] Developed on the basis of qualitative interview data,[69] 78 distinct leadership qualities were identified and categorised into seven desired leadership types (problem-solver; contact-maker; responsible-maker; protector; trust-creator; recogniser; encourager), each type scored on a five-point Likert scale based on the frequency of which supervisors were perceived to display the leadership type in question (0 = never; 1 = seldom; 2 = sometimes; 3 = often; 4 = very often). A mean score of the seven items was calculated ($\alpha = .94$), where higher scores indicated higher levels of desired leadership qualities.

Travels and worksite factors (reflecting degree to which employees are under direct monitoring by co-workers and supervisors) were measured in terms of whether the job included *job travels* (no; yes), *working from home* (no; yes), and *working from holiday home* (no; yes).

For peer review only - http://bmjopen.bmj.com/site/about/guidelines.xhtml

Workplace alcohol use climate was conceptualised as employees' perceptions of *drinking social norms*, measured with the four items from the Drinking Norms Scale (DNS) that directly relates to the work situation.[26] Responses were provided on a four-point Likert scale where higher scores indicated more liberal drinking social norms. A mean score was calculated (α = .73).

An overview of workplace factors is presented in Table 2.

Table 2. Overview of workplace factors

Main factor	Factors	Measurement
Employment characteristics	Working hours	Continuous[A]: number of hours during a typical day
	Work schedule	Categorical[A]: day job, no weekends; day job with weekends; evening job; night job; shift work
	Job position	Categorical[A]: worker; supervisor
	Job size	Categorical[A]: part-time; full-time
	Income	Continuous[A]: gross annual household income in 100000 NOK
Job demands and support factors	Psychological job demands	JCQ: mean score of five items
	Job control	JCQ: mean score of nine items
	Workplace social support	JCQ: mean score of eight items
	Supervisors' leadership qualities	7LQQ: mean score of seven items
Travels and worksite factors	Job travels	Categorical[A]: no; yes
	Working from home	Categorical[A]: no; yes
	Working from holiday home	Categorical[A]: no; yes
Workplace drinking social norms	Drinking social norms	DNS: mean score of four items

[A]Self-developed measure; NOK = Norwegian Kroner; JCQ = Job Content Questionnaire; 7LQQ = Seven-item Leadership Qualities Questionnaire; DNS = Drinking Norms Scale

Covariates

The following sociodemographic factors, that have demonstrated significant associations with alcohol use in a Norwegian employee population,[10] were included as controls in adjusted analyses: *Gender* (male; female), *age* (years), *educational attainment* (primary/lower secondary; upper secondary; university/college ≤4 years; university/college >4 years), *living*

For peer review only - http://bmjopen.bmj.com/site/about/guidelines.xhtml

status (living alone; living with others), *marital status* (unmarried; married), and *number of children*. Due to the cross-sectional data in this study being collected over a period of approximately five years, *year of data collection* (2015; 2016; 2017; 2018; 2019) was included as an additional covariate.

Analysis

Descriptive analyses were performed on the main study variables (outcomes and predictors) and presented in terms of means, standard deviations, frequencies and percentages, as appropriate.

Main analyses were conducted in two stages: model selection and predictor comparison.[70] In the model selection stage, associations between workplace factors and alcohol consumption (AUDIT-C scale 0-12) were explored by means of a multiple hierarchical linear regression analysis. Employment characteristics were entered as predictors in the first model, followed by sequential inclusion of job demands and support factors (Model 2), travels and worksite factors (Model 3), workplace drinking social norms (Model 4), and covariates (Model 5). Unstandardised (b) and standardised (β) regression coefficients were calculated. Potential multicollinearity was explored using the variance inflation factor (VIF). Multicollinearity was deemed a concern if VIFs exceeded 10.[71] Associations between workplace factors and the odds of alcohol-related problems (AUDIT-10 categories 0-7 vs. 8-40) were explored with a multiple hierarchical binary logistic regression analysis. Predictors and covariates were entered sequentially through five models, and odds ratios were calculated. Goodness of fit was explored by means of Hosmer and Lemeshow tests, where poor model fit was indicated by p <.05.[72] In the final and fully adjusted models in the regression analyses (linear and logistic), associations between workplace factors and alcohol outcomes were adjusted for gender, age, educational attainment, living status, marital status,

For peer review only

number of children and year of data collection. Work schedule (categorical nominal predictor) was dummy coded. All main analyses were performed with IBM SPSS version 27.

In the predictor comparison stage, the relative importance of predictors that demonstrated significant associations with the outcomes (p <.05) in fully adjusted models in the model selection stage were estimated by means of dominance analysis (DA).[73] DA consists of examining R^2 values for all possible subset models in a multiple regression, with additional contribution of each predictor. A predictor is considered to be more important than its competitors if its predictive ability exceeds the others' in all subset regressions.[73, p. 545] Each predictor's relative importance was expressed in terms of its average contribution (in percentage points) to the explained variance in the outcome. DA was conducted separately for each outcome using Stata version 17.

Significant results were defined as p <.05.

Patient and public involvement

Employee representatives and other relevant stakeholders were included in the WIRUS project's reference group. Design, research questions, recruitment procedures and choice of outcome measures in the project have been informed by priorities, experience and preferences expressed by the reference group.

RESULTS

Approximately one out of ten employees (11.5 %) reported alcohol-related problems (AUDIT ≥8). The sample's means score on alcohol consumption was 3.2 (on the AUDIT-C scale ranging from 0 to 12). The majority of employees were workers (79.6 % of the total sample) with full-time (81.8 % of the total sample) day job schedules without weekends (73.9 % of the total sample). Employees' average gross annual household income was 1,012,000

For peer review only - http://bmjopen.bmj.com/site/about/guidelines.xhtml

NOK (approximately 83,587 GBP), and a minority of employees had a job that included job travels (14.9 % of the total sample), working from home (6.9 % of the total sample) and working from holiday home (3.0 % of the total sample). Job control and workplace social support were rated higher than psychological job demands (on the JCQ scales ranging from 1 to 4: $M_{\text{Job control}} = 3.0$; $M_{\text{Social support}} = 3.2$; $M_{\text{Psychological job demands}} = 2.2$). Employees' mean rating of supervisors' desired leadership qualities was 2.9 (on the 7LQQ scale ranging from 0 to 4). On average, employees reported somewhat more restrictive than liberal workplace drinking social norms (*M* = 2.2 on the DNS scale ranging from 1 to 4). Descriptive statistics for the main study variables are presented in Table 3.

Table 3. Descriptive statistics for the main study variables

	Variable	
O	Alcohol consumption[A], *M* (*SD*)	3.21 (1.87)
O	Alcohol-related problems[B], n (%) yes	619 (11.50)
**Employment characteristics:**		
P	Working hours[C], *M* (*SD*)	7.95 (1.83)
P	Day job, no weekends, n (%) yes	3982 (73.90)
P	Evening job, n (%) yes	22 (0.40)
P	Night job, n (%) yes	84 (1.60)
P	Shift work, n (%) yes	981 (18.20)
P	Job position, n (%) workers	4288 (79.60)
P	Job size, n (%) full-time	4407 (81.80)
P	Income[D], *M* (*SD*)	10.12 (6.25)
**Job demands and support factors:**		
P	Psychological job demands[E], *M* (*SD*)	2.17 (0.43)
P	Job control[E], *M* (*SD*)	3.00 (0.34)
P	Workplace social support[E], *M* (*SD*)	3.19 (0.47)
P	Leadership qualities[F], *M* (*SD*)	2.91 (0.81)
**Travels and worksite factors:**		
P	Job travels, n (%) yes	804 (14.90)
P	Working from home, n (%) yes	374 (6.90)
P	Working from holiday home, n (%) yes	161 (3.00)
**Workplace drinking social norms:**		
P	Drinking social norms[G], *M* (*SD*)	2.23 (0.53)

N = 5388; O = outcome; P = predictor; *M* = mean; *SD* = standard deviation; [A]Alcohol Use Disorders Identification Test – Consumption (AUDIT-C), scale 0-12; [B]AUDIT (all ten items, scale 0-40), sum score ≥8; [C]Number of hours

For peer review only - http://bmjopen.bmj.com/site/about/guidelines.xhtml

during a typical day; [D]Gross annual household income in 100,000 Norwegian Kroner; [E]Job Content Questionnaire (JCQ), scale 1-4; [F]Seven-item Leadership Qualities Questionnaire (7LQQ), scale 0-4; [G]Drinking Norms Scale (DNS), scale 1-4 (higher scores = more liberal norms)

Bivariate correlations between all study variables (outcomes, predictors and covariates) are presented in the Supplementary File (Table S1).

Workplace factors and alcohol consumption

Associations between workplace factors and alcohol consumption are presented in Table 4.

For peer review only

For peer review only - http://bmjopen.bmj.com/site/about/guidelines.xhtml

Table 4. Associations between workplace factors and alcohol consumption[A]

	Model 1<br>Employment characteristics	Model 2<br>Job demands and support factors added	Model 3<br>Travels and worksite factors added	Model 4<br>Workplace drinking social norms added	Model 5<br>Fully adjusted model, including covariates[B]
	b (β) [*p*]	b (β) [*p*]	b (β) [*p*]	b (β) [*p*]	b (β) [*p*]
Working hours	-.01 (-.00) [.750]	.00 (.00) [.905]	-.00 (-.00) [.831]	**-.03 (-.03) [.029]**	**-.03 (-.03) [.049]**
Day job, no weekends[C]	**-.25 (-.06) [.022]**	**-.28 (-.07) [.011]**	**-.25 (-.06) [.020]**	-.12 (-.03) [.231]	-.04 (-.01) [.669]
Evening job[C]	.41 (.01) [.320]	.39 (.01) [.346]	.43 (.01) [.303]	.21 (.01) [.574]	.07 (.00) [.840]
Night job[C]	.02 (.00) [.920]	-.06 (-.00) [.798]	-.02 (-.00) [.924]	.12 (.01) [.590]	-.04 (-.00) [.867]
Shift work[C]	.06 (.01) [.619]	.04 (.01) [.745]	.07 (.02) [.550]	.09 (.02) [.441]	-.06 (-.01) [.591]
Job position[D]	.11 (.02) [.109]	**.14 (.03) [.040]**	.10 (.02) [.118]	**.25 (.06) [<.001]**	**.25 (.06) [<.001]**
Job size[E]	**.46 (.09) [<.001]**	**.46 (.09) [<.001]**	**.44 (.09) [<.001]**	**.31 (.06) [<.001]**	**.18 (.04) [.006]**
Income[F]	.00 (.00) [.760]	.00 (.01) [.716]	.00 (.00) [.966]	.00 (.00) [.840]	**.02 (.05) [<.001]**
Psych. job demands[G]	-	**-.15 (-.03) [.017]**	**-.15 (-.03) [.016]**	**-.13 (-.03) [.026]**	-.09 (-.02) [.106]
Job control[G]	-	-.12 (-.02) [.144]	-.15 (-.03) [.063]	-.10 (-.02) [.165]	.02 (.00) [.755]
Workplace social support[G]	-	**.15 (.04) [.030]**	**.16 (.04) [.023]**	**.14 (.03) [.035]**	**.13 (.03) [.040]**
Leadership qualities[H]	-	**-.09 (-.04) [.028]**	**-.09 (-.04) [.033]**	**-.10 (-.04) [.005]**	**-.10 (-.04) [.004]**
Job travels[I]	-	-	**.25 (.05) [.001]**	.04 (.01) [.564]	.04 (.01) [.575]
Working from home[I]	-	-	-.05 (-.01) [.647]	-.18 (-.02) [.069]	-.12 (-.02) [.216]
Working from holiday home[I]	-	-	**.40 (.04) [.012]**	**.42 (.04) [.003]**	**.40 (.04) [.007]**
Drinking social norms[J]	-	-	-	**1.51 (.42) [<.001]**	**1.37 (.39) [<.001]**
R^2	.012	.014	.018	.192	.225
VIF range	1.02 – 3.54	1.03 – 3.56	1.03 – 3.59	1.03 – 3.60	1.08 – 3.62

N = 5388; Results from multiple hierarchical linear regression analyses; b = unstandardised regression coefficient; β = standardized regression coefficient; VIF = variance inflation factor; [A]Alcohol Use Disorders Identification Test – Consumption (AUDIT-C), scale 0-12, higher scores indicate higher consumption; [B]Covariates = gender, age, educational attainment, living status, marital status, number of children, year of data collection; [C]Ref. = other work schedules; [D]Ref. = worker; [E]Ref. = part-time; [F]Household gross annual income in 100,000 Norwegian Kroner; [G]Job Content Questionnaire (JCQ), scale 1-4, higher scores indicate higher predictor values; [H]Seven-item Leadership Qualities Questionnaire (7LQQ), scale 0-4, higher scores indicate higher level of desired leadership qualities; [I]Ref. = no; [J]Drinking Norms Scale (DNS), scale 1-4, higher scores indicate more liberal workplace drinking norms; Bold typeface indicates statistically significant associations (p <.05)

For peer review only - http://bmjopen.bmj.com/site/about/guidelines.xhtml

As shown in Table 4 (Models 1-3), employment characteristics, job demands and support factors and travels and worksite factors explained only small proportions of the variance in alcohol consumption (1.2 to 1.8 %). The proportion of explained variance in consumption increased considerably (to 19.2 %) when workplace drinking social norms was included in the analysis (Model 4). In the fully adjusted model (Model 5), also taking covariates into account, eight workplace factors were significantly associated with alcohol consumption. The highest VIF was 3.62, indicating that multicollinearity was not a concern.

Higher consumption was associated with shorter working hours, being a supervisor (versus a worker), working full-time (as opposed to part-time), higher income, higher levels of workplace social support, having a supervisor with less desired leadership qualities, working from holiday home, and more liberal workplace drinking social norms. The remaining workplace factors either failed to demonstrate significant relationships with alcohol consumption altogether (evening job schedule, night job schedule, shift work schedule, job control, working from home), or failed to reach statistical significance in the fully adjusted model (day job without weekend schedule, psychological job demands, job travels).

The relative importance of the eight significant workplace factors for explaining the variance in alcohol consumption is presented in Table 5.

Table 5. Relative importance of workplace factors[A] in explaining alcohol consumption[B]

Predictor	Dominance statistic	Average contribution (pp)[C]	Ranking
Drinking social norms	0.1792	17.92	1
Job size	0.0045	0.45	2
Working from holiday home	0.0016	0.16	3
Job position	0.0013	0.13	4
Leadership qualities	0.0007	0.07	5
Working hours	0.0005	0.05	6
Workplace social support	0.0002	0.02	7
Income	0.0000	0.00	8

N = 5388; Results from dominance analysis; [A]Workplace factors that demonstrated significant associations with alcohol consumption in the fully adjusted regression model (Model 5 in Table 4); [B]Alcohol Use

For peer review only - http://bmjopen.bmj.com/site/about/guidelines.xhtml

Disorders Identification Test – Consumption (AUDIT-C), scale 0-12; [C]Average contribution to the R^2 in percentage points

Based on average contribution to the explained variance in alcohol consumption, drinking social norms (17.92 %) was the dominant predictor, followed by job size (0.45 %), working from holiday home (0.16 %), job position (0.13 %), leadership qualities (0.07 %), working hours (0.05 %), workplace social support (0.02 %) and income (0.00 %). In the fully adjusted regression model (Table 4, Model 5), a one-unit increase in drinking social norms (towards more liberal) was associated with an increase of 1.37 units on the alcohol consumption scale (ranging from 0 to 12).

Workplace factors and alcohol-related problems

Associations between workplace factors and odds of having alcohol-related problems are presented in Table 6.

For peer review only - http://bmjopen.bmj.com/site/about/guidelines.xhtml

For peer review only

Table 6. Associations between workplace factors and odds of having alcohol-related problems[A]

	Model 1 Employment characteristics	Model 2 Job demands and support factors added	Model 3 Travels and worksite factors added	Model 4 Workplace drinking social norms added	Model 5 Fully adjusted model, including covariates[B]
	OR (*p*)	OR (*p*)	OR (*p*)	OR (*p*)	OR (*p*)
Working hours	0.97 (.270)	0.98 (.315)	0.97 (.247)	**0.95 (.025)**	**0.94 (.024)**
Day job, no weekends[C]	0.74 (.079)	0.73 (.074)	0.74 (.082)	0.82 (.278)	0.97 (.852)
Evening job[C]	2.35 (.117)	2.30 (.128)	2.35 (.119)	1.98 (.235)	1.77 (.334)
Night job[C]	1.59 (.212)	1.48 (.299)	1.52 (.268)	1.68 (.182)	1.48 (.328)
Shift work[C]	1.05 (.812)	1.02 (.921)	1.04 (.822)	1.06 (.761)	0.83 (.373)
Job position[D]	0.82 (.082)	0.83 (.107)	0.80 (.067)	0.95 (.646)	0.99 (.956)
Job size[E]	**1.92 (<.001)**	**1.91 (<.001)**	**1.88 (<.001)**	**1.68 (<.001)**	1.31 (.064)
Income[F]	**1.00 (.001)**	**1.00 (.001)**	**1.00 (.001)**	**1.00 (<.001)**	1.00 (.764)
Psych. job demands[G]	-	1.02 (.880)	1.02 (.869)	1.03 (.780)	1.05 (.672)
Job control[G]	-	0.82 (.142)	0.79 (.086)	0.82 (.175)	0.92 (.565)
Workplace social support[G]	-	0.97 (.764)	0.97 (.812)	0.90 (.407)	0.88 (.331)
Leadership qualities[H]	-	0.94 (.317)	0.94 (.329)	0.92 (.223)	0.90 (.127)
Job travels[I]	-	-	**1.35 (.016)**	1.12 (.383)	1.09 (.506)
Working from home[I]	-	-	0.99 (.964)	0.86 (.435)	0.91 (.646)
Working from holiday home[I]	-	-	1.09 (.749)	1.15 (.599)	1.17 (.564)
Drinking social norms[J]	-	-	-	**4.61 (<.001)**	**3.52 (<.001)**
$R^2_{Nagelkerke}$	.020	.022	.025	.125	.184
Hosmer and Lemeshow test	χ = 12.39, *p* = .135	χ = 13.63, *p* = .092	χ = 6.21, *p* = .624	χ = 9.39, *p* = .310	χ = 8.22, *p* = .412

N = 5388; Results from multiple binary logistic regression analyses; OR = odds ratio; [A]Alcohol Use Disorders Identification Test (AUDIT, all ten items), sum score 0-7 (no problems) vs 8-40 (problems); [B]Covariates = gender, age, educational attainment, living status, marital status, number of children, year of data collection; [C]Ref. = other work schedules; [D]Ref. = worker; [E]Ref. = part-time; [F]Household gross annual income in 100,000 Norwegian Kroner; [G]Job Content Questionnaire (JCQ), scale 1-4, higher scores indicate higher predictor values; [H]Seven-item Leadership Qualities Questionnaire (7LQQ), scale 0-4, higher scores indicate higher level of desired leadership qualities; [I]Ref. = no; [J]Drinking Norms Scale (DNS), scale 1-4, higher scores indicate more liberal workplace drinking norms; Bold typeface indicates statistically significant associations (*p* <.05)

For peer review only - http://bmjopen.bmj.com/site/about/guidelines.xhtml

As shown in Table 6 (Models 1-3), employment characteristics, job demands and support factors and travels and worksite factors explained only small proportions of the variance in odds of having alcohol-related problems (2.0 to 2.5 %). The explained variance increased considerably when workplace drinking social norms were included in the analysis (Model 4, 12.5 %). In the fully adjusted model (Model 5, containing all workplace factors as well as covariates), only two workplace factors were significantly related with odds of having alcohol-related problems. There was no evidence of poor model fits, as indicated by Hosmer and Lemeshow tests reaching $p \geq .05$.

Elevated odds of alcohol-related problems was associated with shorter working hours and more liberal workplace drinking social norms. The remaining workplace factors either failed to reach statistical significance in the fully adjusted model (job size, income, job travels, working from home), or were unrelated with alcohol-related problems altogether (day job no weekend schedule, evening schedule, night schedule, shift work schedule, job position, psychological job demands, job control, workplace social support, leadership qualities, working from holiday home).

Dominance analysis (Table 7) revealed that drinking social norms was the dominant predictor of alcohol-related problems by having an average contribution to the proportion of explained variance of 5.26 %. In contrast, the contribution of working hours was only 0.04 % (on average).

Table 7. Relative importance of workplace factors[A] in explaining odds of having alcohol-related problems[B]

Predictor	Dominance statistic	Average contribution (pp)	Ranking
Drinking social norms	0.0526	5.26	1
Working hours	0.0004	0.04	2

N = 5388; Results from dominance analysis; [A]Workplace factors that demonstrated significant associations with odds of having alcohol-related problems in the fully adjusted regression model (Model 5 in Table 6); [B]Alcohol Use Disorders Identification Test (AUDIT, all ten items), sum score 0-7 (no problems) vs 8-40 (problems); [C]Average contribution to the R^2 in percentage points

For peer review only - http://bmjopen.bmj.com/site/about/guidelines.xhtml

A one-hour increase in working hours was associated with a reduced odds of alcohol-related problems by a factor of 0.94. A one-unit increase on the drinking social norms scale (towards more liberal) was associated with an increased odds of alcohol-related problems by a factor of 3.52.

Summary of main results

Main results are summarised in Figure 1.

[Figure 1 about here]

Eight out of 16 workplace factors were not significantly related to either alcohol consumption or alcohol-related problems. Six workplace factors were significantly associated with alcohol consumption but not with alcohol-related problems (job position, job size, income, workplace social support, leadership qualities, working from holiday home). Two workplace factors were significantly related to both alcohol consumption and alcohol-related problems (working hours, drinking social norms). With the exception of drinking social norms (that was the dominant predictor of alcohol consumption as well as of alcohol-related problems), associations between workplace factors and the alcohol outcomes were quite weak (see Tables 4 and 6) and most of the variance in employees' alcohol consumption and alcohol-related problems was explained by variables not included in this study, i.e., by variables beyond the included workplace factors.

DISCUSSION

For peer review only - http://bmjopen.bmj.com/site/about/guidelines.xhtml

The aim of this study was to explore whether workplace factors were associated with alcohol use in a heterogeneous sample of employees in Norway.

Drinking social norms emerged as the supremely most important predictor of both alcohol consumption and alcohol-related problems. More liberal attitudes toward the role of alcohol in workplace settings was associated with having a higher consumption as well as elevated odds of having alcohol-related problems. Drinking norms prescribe the level of consumption that is considered appropriate or tolerable in specific social contexts, and employees' drinking norms may reflect the workplace drinking culture.[26, 74-76] Hence, our study supports previous research demonstrating that workplace alcohol use climate may affect employees' level and experienced consequences of alcohol consumption.[25-28] The association between workplace drinking norms and alcohol consumption can be viewed in conjunction with evidence that has demonstrated that a significant proportion of employees' overall consumption indeed does occur in job-related contexts. Studies from Norway have found that 30 % of employees had consumed alcohol in job-related settings during the past two weeks,[77] 43 % of employees' overall consumption occurred in job-related situations,[77] and employers initiated and organised more than half of the job-related situations in which employees were exposed to alcohol.[78]

Interestingly, longer work hours were associated with lower consumption but also with higher odds of having alcohol-related problems. These findings may, intuitively, stand out as somewhat contradictory, insofar that one may expect alcohol consumption to be closely associated with alcohol-related problems. We did find a considerable and statistically significant correlation between the two alcohol outcomes. However, the strength of the correlation was in the upper layer of moderate strength (see Supplementary File, Table S1), which is far from a perfect correlation. Hence, somewhat different mechanisms may underlie the two alcohol outcomes, which has been demonstrated in earlier research. As captured by

For peer review only - http://bmjopen.bmj.com/site/about/guidelines.xhtml

the term the alcohol harm paradox, certain populations may be exposed to a variety of health risks that interact with alcohol consumption to create synergistically detrimental effects of consumption,[79] which result in greater alcohol-related harm among individuals with specific characteristics (e.g., socioeconomic variables) for each consumed alcohol unit, compared with individuals without these specific characteristics.[24] Associations between working hours and alcohol outcomes have been explained in terms of stress.[29, 34, 80] Shorter working hours may be related to higher consumption (as found in our study) due to involuntary part-time and/or temporary contracts generating stress that is coped with by means of higher alcohol consumption.[34] On the other hand, particularly long working hours may be associated with particularly high work loads and stress that result in coping mechanisms generating alcohol-related problems.[34, 80] However, our study did not allow causal explanations for the differential findings regarding the relationships between working hours, consumption and alcohol-related problems. It should be noted that associations found in our study were quite weak and that we, in line with earlier researchers, were unable to establish consistent knowledge regarding the relationship between working hours and alcohol outcomes among employees.[29-34]

Higher income was associated with higher alcohol consumption. This finding is consistent with earlier studies emphasising that higher socioeconomic status is related to higher levels of consumption.[23, 42, 43] Alcohol is a costly commodity,[44] and high-income employees have better access to alcohol than their lower-income counterparts.

Working full-time (as opposed to part-time) was associated with elevated alcohol consumption among employees. This finding contradicts earlier research from the USA that has found that part-time workers had higher weekly consumption than full-time workers.[41] Our data do not illuminate reasons for why full-time employees were more exposed to alcohol than part-time employees. One may hypothesise that this association can be interpreted in

For peer review only - http://bmjopen.bmj.com/site/about/guidelines.xhtml

terms of the degree to which employees are socially integrated at the workplace. Part-time employees may have a lower workplace social integration than their full-time counterparts, perhaps resulting in a lower exposure to alcohol in job-related situations. Alternatively, part-time employees may have reduced working hours due to health problems,[81] which in turn may explain why they – on a group level – consume less alcohol than full-time employees. Employees' financial situation may as well be of importance, insofar that alcohol is a costly commodity,[44] and that part-time workers may find themselves in a more strained financial situation than full-time employees.

Workers consumed less alcohol than supervisors, which is in line with some earlier studies.[32, 40] One may hypothesise that a supervisor position entails access and exposure to factors that may enhance alcohol consumption, such as higher income and greater work location flexibility (e.g., working from holiday home). However, our data do not lend themselves to explanations for the association between job position and alcohol consumption, and several studies have failed to demonstrate such a relationship.[23, 31, 34, 35]

Worksite factors (e.g., working from home and having overall greater flexibility) and job travels have in some studies been linked with elevated alcohol consumption.[35, 40, 47] In our study, travels and worksite factors demonstrated mixed results. We did not find an association between alcohol consumption, working from home and job travels. However, working from holiday home was associated with elevated consumption. These findings may reflect a context-dependency in the relationship between work location flexibility and alcohol use. In an earlier study exploring relationships between activity patterns and alcohol use among employees in Norway,[82] non-domestic activities were associated with higher consumption than domestic activities. Having an activity pattern characterised by activities within (rather than outside) the household was associated with lower alcohol consumption. Working from home (in a domestic setting) may constitute working in an activity setting not

For peer review only

For peer review only - http://bmjopen.bmj.com/site/about/guidelines.xhtml

very compatible with alcohol use, even though working from home entails being less visible for co-workers and supervisors and less susceptible to workplace social control (factors which have been linked with elevated alcohol consumption).[9, 22] Working from holiday home constitutes a non-domestic activity where work is performed in a context more similar to vacations. Vacations and holidays typically involve greater alcohol exposure, potentially resulting in higher consumption levels.[83, 84]

Having supervisors with desired leadership qualities was associated with lower alcohol consumption. Supervisors who are highly rated on desired leadership qualities (problem-solving, contact-making, responsible-making, protecting, trust-creating, recognising and encouraging;[69]) may be perceived as active, visible and involved with their staff and can be well-positioned to set norms and exert informal social control. It has been advocated that visible and active supervisors represent protective factors for elevated alcohol use among workers,[46] and studies have demonstrated lower prevalence of drinking and hangover episodes at work among employees employed at workplaces where supervisors exert active social control.[25, 50, 51] As such, supervisors with desired leadership qualities may be influential in determining workplace drinking cultures. One may assume that desired leadership qualities represent a potential protective factor for elevated alcohol consumption in line with how earlier studies have found such desired leadership to be a protective factor for mental distress,[85] and physiological pain.[86, 87]

In contrast to earlier studies that have found that higher levels of social support at work are associated with lower alcohol consumption among employees,[33, 38, 55] we found an association in the opposite direction, i.e., that higher levels of support were associated with higher consumption. Social support may be conceptualised as a proxy of social integration,[88] and our measure of social support included support from both peers and supervisors. Perceived levels of social support may reflect the level of which the individual

For peer review only - http://bmjopen.bmj.com/site/about/guidelines.xhtml

employee feels socially integrated in the workplace culture. Hence, social support may have dual and opposing effects on alcohol outcomes, depending on the workplace culture. In liberal drinking cultures, high levels of perceived support may reinforce permissive drinking norms,[89] while the opposite may be true in more restrictive workplace drinking cultures.

Methodological considerations

The cross-sectional design of this study precludes causal inferences regarding the relationships between workplace factors and alcohol-related outcomes. Even though we attempted to minimise risk of confounding by including relevant covariates in adjusted analyses, reversed, reciprocal and/or third-variable causation cannot be ruled out. For instance, the association between job size and alcohol use may be due to job size affecting drinking pattern, to drinking pattern affecting job size, or to an unmeasured third variable (e.g., health status) affecting both job size and drinking pattern. However, the aim of this study was not to unravel causal relationships but was explorative in nature. The cross-sectional design may also have played a role in explaining why observed effect sizes were quite small. Potential effects of some workplace factors on broad indicators of alcohol use and problems may be temporary and/or short-lived, rendering it difficult to fully capture true effects without more robust research designs that involve longitudinal measures.

The sample was large and consisted of a heterogeneous selection of employees across work sectors, divisions, levels and geographical locations. The sample size well exceeded the a priori defined criteria,[61, 62] and was thus considered statistically satisfactory (required sample size: N = 435; actual sample size: N = 5388). However, the response rate was quite low (27.7 %). Low response rates may represent a threat to external validity by limiting generalisability of results in instances where responders systematically differ from non-responders. Study selection analyses of the WIRUS screening data indicate that the final study

For peer review only - http://bmjopen.bmj.com/site/about/guidelines.xhtml

sample was similar to the invited sample (i.e., all employees in the included companies) regarding distributions of gender and age.[90] However, compared with the entire Norwegian workforce, female, older and well-educated employees were overrepresented in our study. Moreover, it must be noted that public sector employees were largely overrepresented, resulting in a limited potential of generalising results across employment sectors. Probable reasons for this overrepresentation include that public companies in Norway, on average, tend to be larger than private companies (the project aimed to recruit companies with >100 employees), and that an economic downturn in Norway in 2014-2015 (due to falling oil prices) made it difficult for private (profit-based) companies to prioritise participation in research. There may also be important differences between the study sample and non-responders that we lack information about (unmeasured selection effects) that have biased our findings. Moreover, a considerable number of employees (n = 3154) were excluded due to not responding on all study items in the survey. Comparisons between completers and non-completers on sociodemographic variables (see Supplementary File, Table S2) demonstrated that completers were characterised by a slight overrepresentation of males, employees with university/college education, married employees, and employees having more children.

This study was solely based on self-reported data from employees, which may involve risks of measuring bias (e.g., recall bias and social desirability bias). In particular, one may assume that alcohol use may have been underestimated, insofar that studies have demonstrated a discrepancy between self-reported consumption and actual alcohol sales.[91] On the other hand, alcohol consumption and alcohol-related problems were both measured with the AUDIT, which has demonstrated psychometric properties superior to other alcohol screening instruments,[92] and has been deemed appropriate for use in Norwegian employee populations.[93] A threshold of eight points on the AUDIT as indicative of alcohol-related problems has been found to represent a satisfactory compromise between sensitivity and

For peer review only - http://bmjopen.bmj.com/site/about/guidelines.xhtml

specificity,[7] and are in line with previous research on non-clinical populations.[94-97] Furthermore, regarding issues of measurement, we only differentiated between workers and supervisors, i.e., not taking into account how many workers supervisors were responsible for. This may have concealed some potential nuances regarding stress and workload among supervisors. We did, however, include psychological job demands as a predictor in this study, which may have captured some of these potential nuances.

Implications

Among the workplace factors explored in this study, drinking social norms emerged as the supremely most important predictor of employee alcohol use. Hence, results from our study calls attention to the importance of workplace drinking cultures to understand employees' level of alcohol consumption and odds of experiencing alcohol-related problems. Furthermore, other significant predictors of employee alcohol use support the notion of emphasising workplace drinking culture. Perceiving higher levels of social support and being full-time employed were both associated with higher consumption levels and can both be conceptualised as proxies of social integration at the workplace, thereby indicating the importance of workplace culture. Moreover, results indicate the importance of supervisor behaviour. Supervisors consumed more alcohol than workers, and having supervisors with high levels of desired leadership qualities was associated with lower levels of consumption among employees. Efforts aimed at reducing alcohol consumption and alcohol-related problems among employees may include fostering less liberal drinking cultures at workplaces in combination with desired leadership qualities among managers. Raising managers' awareness of their influence on workplace drinking culture may be serviceable. Prosocial supervisors may stimulate workplace cultures that discourage or inhibit drinking in work-related situations and may also foster social relationships and trust that enable establishment

For peer review only - http://bmjopen.bmj.com/site/about/guidelines.xhtml

of routines for early identification and aid for employees who may benefit from alcohol prevention interventions.

In a systematic review,[98] collaboration/teamwork and positive, accessible and fair supervisors were identified among the most pronounced factors considered as important for a healthy workplace. In another systematic review,[99] covering workplace resources to improve employee well-being and performance, social support and high-quality relationships between workers and supervisors were identified among the factors organisations should emphasise. As such, a focus on workplace factors identified in our study as potential protective factors for alcohol-related problems seem to converge with the WHO framework for healthy workplaces, i.e., workplaces "in which workers and managers collaborate to use a continual improvement process to protect and promote health, safety and well-being of all workers and the sustainability of the workplace by considering" important aspects such as the psychosocial work environment, the organisation of work, and the workplace culture.[100, p. 16]

Evidence on associations between workplace factors and employee alcohol use still stands out as complex and nuanced, and this study showed that only marginal proportions of the variance in alcohol consumption and alcohol-related problems were explained by employment characteristics, job demands and support factors and travels and worksite factors. Hence, further research is warranted. Results from this study suggest the application and further development of interventions, e.g., Workplace Health Promotion Programs (WHPP), targeting workplace drinking culture and leadership. Effects and implementation of such interventions should be explored by means of robust research designs, such as prospective cohort studies and cluster randomised controlled trials. Future research may also benefit from explorations of potential interactions between individual and workplace factors, including mediators and moderators, which were beyond the scope of our study.

For peer review only - http://bmjopen.bmj.com/site/about/guidelines.xhtml

Conclusions

Knowledge about workplace factors associated with employee alcohol use is important as an aid in determining the extent to which employers should make workplace-based alcohol prevention interventions an overall priority. This study, conducted in a large and heterogeneous sample of employees in Norway points to the importance of drinking social norms, workplace drinking culture and leadership.

DECLARATIONS

Ethics approval statement

Companies and employees were provided with information about the study's aim, they were assured confidentiality, that participation was voluntary, and that they could withdraw from the study at any given time. Written informed consent was obtained from all included employees. The study was approved by the Regional Committee for Medical and Health Research in Norway (REK; approval no. 2014/647).

Author contributions

RWA is the principal investigator (PI) and project manager (PM) for the WIRUS project (Workplace Interventions preventing Risky alcohol Use and Sick leave). This study was designed by MMT and RWA. MMT analysed the data and drafted the manuscript. JCS, TB, LSS, RWA and MMT provided scientific input to the different drafts and provided data interpretation. All authors made critical revisions and provided intellectual content to the manuscript, approved the final version to be published, and agreed to be accountable for all aspects of this work.

For peer review only - http://bmjopen.bmj.com/site/about/guidelines.xhtml

Funding

This work was supported by the Norwegian Directorate of Health (grant number: n/a) and the Research Council of Norway (grant number: 260640).

Competing interests

None declared.

Data availability statement

De-identified data from the WIRUS Screening study are available from the corresponding author on reasonable request.

For peer review only

SUPPLEMENTARY FILE

Supplementary File: Correlation analyses and study selection analyses (PDF)

REFERENCES

1 Griswold MG, Fullman N, Hawley C, et al. Alcohol use and burden for 195 countries and territories, 1990–2016: a systematic analysis for the Global Burden of Disease Study 2016. *Lancet* 2018;392:1015-35. doi: 10.1016/s0140-6736(18)31310-2

2 Rehm J, Gmel GE, Gmel G, et al. The relationship between different dimensions of alcohol use and the burden of disease - an update. *Addiction* 2017;112:968-1001. doi: 10.1111/add.13757

3 Room R, Babor T, Rehm J. Alcohol and public health. *Lancet* 2005;365:519-30. doi: 10.1016/s0140-6736(05)70276-2

4 Wood AM, Kaptoge S, Butterworth AS, et al. Risk thresholds for alcohol consumption: combined analysis of individual-participant data for 599 912 current drinkers in 83 prospective studies. *Lancet* 2018;391:1513-23. doi: 10.1016/S0140-6736(18)30134-X

5 World Health Organization. Global status report on alcohol and health 2018. Geneva, Switzerland: World Health Organization 2018.

6 Vos T, Lim SS, Abbafati C, et al. Global burden of 369 diseases and injuries in 204 countries and territories, 1990–2019: a systematic analysis for the Global Burden of Disease Study 2019. *Lancet* 2020;396:1204-22.

For peer review only - http://bmjopen.bmj.com/site/about/guidelines.xhtml

7 Babor TF, Higgins-Biddle JC, Saunders JB, et al. AUDIT: The alcohol use disorders identification test. Guidelines for use in primary health care. 2nd ed. Geneva, Switzerland: World Health Organization 2001.

8 Collin J, Casswell S. Alcohol and the sustainable development goals. *Lancet* 2016;387:2582-83. doi: 10.1016/S0140-6736(16)30827-3

9 Frone MR. Alcohol and illicit drug use in the workforce and workplace. Washington, DC: American Psychological Association 2013.

10 Thørrisen MM, Skogen JC, Aas RW. The associations between employees' risky drinking and sociodemographics, and implications for intervention needs. *BMC Public Health* 2018;18 doi: 10.1186/s12889-018-5660-x

11 Amiri S, Behnezhad S. Alcohol consumption and sick leave: a meta-analysis. *J Addict Dis* 2020;38:100-12. doi: 10.1080/10550887.2020.1724606

12 Hashemi N, Skogen JC, Sevic A, et al. A systematic review and meta-analysis uncovering the relationship between alcohol consumption and sickness absence. When type of design, data, and sickness absence make a difference. *PLoS ONE* 2022;17 doi: 10.1371/journal.pone.0262458

13 Marzan M, Callinan S, Livingston M, et al. Systematic review and dose-response meta-analysis on the relationship between alcohol consumption and sickness absence. *Alcohol Alcohol* 2021 doi: 10.1093/alcalc/agab008

14 Schou L, Moan IS. Alcohol use–sickness absence association and the moderating role of gender and socioeconomic status: A literature review. *Drug Alcohol Rev* 2016;35:158-69. doi: 10.1111/dar.12278

15 Ford MT, Cerasoli CP, Higgins JA, et al. Relationships between psychological, physical, and behavioural health and work performance: A review and meta-analysis. *Work & Stress* 2011;25:185-204. doi: 10.1080/02678373.2011.609035

16 Thørrisen MM, Bonsaksen T, Hashemi N, et al. Association between alcohol consumption and impaired work performance (presenteeism): a systematic review. *BMJ Open* 2019;9 doi: 10.1136/bmjopen-2019-029184

17 Kaner EF, Beyer FR, Muirhead C, et al. Effectiveness of brief alcohol interventions in primary care populations. *Cochrane Database Syst Rev* 2018;2:CD004148. doi: 10.1002/14651858.CD004148.pub4

18 O'Donnell P, Anderson P, Newbury-Birch D, et al. The impact of brief alcohol interventions in primary healthcare: A systematic review of reviews. *Alcohol Alcohol* 2014;49:66-78. doi: 10.1093/alcalc/agt170

19 Schulte B, O'Donnell AJ, Kastner S, et al. Alcohol screening and brief intervention in workplace settings and social services: A comparison of literature. *Front Psychiatry* 2014;5 doi: 10.3389/fpsyt.2014.00131

20 Webb G, Shakeshaft A, Sanson-Fisher R, et al. A systematic review of work-place interventions for alcohol-related problems. *Addiction* 2009;104:365-77. doi: 10.1111/j.1360-0443.2008.02472.x

21 Yuvaraj K, Eliyas SK, Gokul S, et al. Effectiveness of workplace intervention for reducing alcohol consumption: a systematic review and meta-analysis. *Alcohol Alcohol* 2019;54:264-71. doi: 10.1093/alcalc/agz024

For peer review only - http://bmjopen.bmj.com/site/about/guidelines.xhtml

22 Frone MR. Predictors of overall and on-the-job substance use among young workers. *J Occup Health Psychol* 2003;8:39-54. doi: 10.1037/1076-8998.8.1.39

23 Marchand A, Parent-Lamarche A, Blanc ME. Work and high-risk alcohol consumption in the Canadian workforce. *Int J Environ Res Public Health* 2011;8:2692-705. doi: 10.3390/ijerph8072692

24 Skogen JC, Bøe T, Thørrisen MM, et al. Sociodemographic characteristics associated with alcohol consumption and alcohol-related consequences, a latent class analysis of the Norwegian WIRUS screening study. *BMC Public Health* 2019;19 doi: 10.1186/s12889-019-7648-6

25 Ames GM, Grube JW, Moore RS. Social control and workplace drinking norms: A comparison of two organizational cultures. *J Stud Alcohol* 2000;61:203-19. doi: 10.15288/jsa.2000.61.203

26 Barrientos-Gutierrez T, Gimeno D, Mangione TW, et al. Drinking social norms and drinking behaviours: a multilevel analysis of 137 workgroups in 16 worksites. *Occup Environ Med* 2007;64:602-08. doi: 10.1136/oem.2006.031765

27 Hodgins DC, Williams R, Munro G. Workplace responsibility, stress, alcohol availability and norms as predictors of alcohol consumption-related problems among employed workers. *Subst Use Misuse* 2009;44:2062-69. doi: 10.3109/10826080902855173

28 Frone MR, Bamberger PA. Alcohol and illicit drug involvement in the workforce and workplace. In: Quick J, Fisher G, Ford M, Tetrick L, eds. Handbook of occupational health psychology. Washington, DC: American Psychological Association In press 2022: chapter 18.

29 Pachito DV, Pega F, Bakusic J, et al. The effect of exposure to long working hours on alcohol consumption, risky drinking and alcohol use disorder: A systematic review and meta-analysis from the WHO/ILO Joint Estimates of the Work-related Burden of Disease and Injury. *Environ Int* 2021;146 doi: 10.1016/j.envint.2020.106205

30 Virtanen M, Jokela M, Nyberg ST, et al. Long working hours and alcohol use: systematic review and meta-analysis of published studies and unpublished individual participant data. *BMJ* 2015;350 doi: 10.1136/bmj.g7772

31 Frone MR. Prevalence and distribution of alcohol use and impairment in the workplace: a U.S. national survey. *J Stud Alcohol* 2006;67:147-56. doi: 10.15288/jsa.2006.67.147

32 Marchand A. Alcohol use and misuse: What are the contributions of occupation and work organization conditions? *BMC Public Health* 2008;8 doi: 10.1186/1471-2458-8-333

33 Saade SL, Marchand A. Work organization conditions, alcohol misuse: The moderating role of personality traits. *Work* 2013;44:191-200. doi: 10.3233/WOR-2012-1408

34 Cheng WJ, Cheng Y, Huang MC, et al. Alcohol dependence, consumption of alcoholic energy drinks and associated work characteristics in the Taiwan working population. *Alcohol Alcohol* 2012;47:372-79. doi: 10.1093/alcalc/ags034

35 Moan IS, Halkjelsvik T. Alkohol og arbeidsliv. En undersøkelse blant norske arbeidstakere [Alcohol and work. A survey among Norwegian employees]. Oslo, Norway: Norwegian Institute of Public Health 2016.

36 Plescia F, Cirrincione L, Martorana D, et al. Alcohol abuse and insomnia disorder: Focus on a group of night and day workers. *Int J Environ Res Public Health* 2021;18 doi: 10.3390/ijerph182413196

For peer review only - http://bmjopen.bmj.com/site/about/guidelines.xhtml

37 Buchvold HV, Pallesen S, Øyane NMF, et al. Associations between night work and BMI, alcohol, smoking, caffeine and exercise - a cross-sectional study. *BMC Public Health* 2015;15 doi: 10.1186/s12889-015-2470-2

38 Ansoleaga E, Montano R, Vezina M. Psychosocial risk at work and hazardous alcohol consumption among Chile's working adults. *Can J Public Health* 2013;104:502-08. doi: 10.17269/cjph.104.4045

39 Hermansson U, Knutsson A, Brandt L, et al. Screening for high-risk and elevated alcohol consumption in day and shift workers by use of the AUDIT and CDT. *Occup Med* 2003;53 doi: 10.1093/occmed/kqg104

40 Nielsen MB, Christensen JO, Knardahl S. Working at home and alcohol use. *Addict Behav Rep* 2021;14 doi: 10.1016/j.abrep.2021.100377

41 Lee CM, Cadigan JM, Fairlie AM, et al. Transitions into young adulthood: Extent to which alcohol use, perceived drinking norms, and consequences vary by education and work statuses among 18-20 year olds. *Addict Behav* 2018;79:107-12. doi: 10.1016/j.addbeh.2017.12.004

42 Bloomfield K, Grittner U, Kramer S, et al. Social inequalities in alcohol consumption and alcohol-related problems in the study countries of the EU concerted action 'gender, culture and alcohol problems: a multi-national study'. *Alcohol Alcohol* 2006;41;26-36. doi: 10.1093/alcalc/agl073

43 Probst C, Manthey J, Rehm J. Understanding the prevalence of lifetime abstinence from alcohol: an ecological study. *Drug Alcohol Depen* 2017;178;126-9. doi: 10.1016/j.drugalcdep.2017.05.008

44 Kan MY, Lan M. Comparing alcohol affordability in 65 cities worldwide. *Drug Alcohol Rev* 2013;32:19-26. doi: 10.1111/j.1465-3362.2012.00476.x

45 Kawakami N, Haratani T, Hemmi T, et al. Prevalence and demographic correlates of alcohol-related problems in Japanese employees. *Soc Psych Psych Epid* 1992;27;198-202. doi: 10.1007/BF00789006

46 Roman PM, Trice HM. The development of deviant drinking behavior. *Arch Environ Health* 1970;20:424-35. doi: 10.1080/00039896.1970.10665615

47 Macdonald S, Wells S, Wild TC. Occupational risk factors associated with alcohol and drug problems. *Am J Drug Alcohol Abuse* 1999;25:351-69. doi: 10.1081/ADA-100101865

48 Bacharach SB, Bamberger PA, Sonnenstuhl WJ. Driven to drink: Managerial control, work-related risk factors, and employee problem drinking. *Acad Manage J* 2002;45:637-58. doi: 10.5465/3069302

49 Nielsen MB, Gjerstad J, Frone MR. Alcohol use and psychosocial stressors in the Norwegian workforce. *Subst Use Misuse* 2018;53:574-84. doi: 10.1080/10826084.2017.1349797

50 Frone MR, Trinidad JR. Testing a general model of employee alcohol use and workplace productivity among U.S. workers. *Alcohol Clin Exp Res* 2012;36:229A.

51 Moore RS, Ames GM, Duke MR, et al. Food service employee alcohol use, hangovers and norms during and after work hours. *Journal of Substance Use* 2012;17 doi: 10.3109/14659891.2011.580414

52 Heikkilä K, Fransson EI, Nyberg ST, et al. Job strain and health-related lifestyle: findings from an individual-participant meta-analysis of 118 000 working adults. *Am J Public Health* 2013;103:2090-97.

For peer review only - http://bmjopen.bmj.com/site/about/guidelines.xhtml

53 Roxburgh S. Gender differences in the effect of job stressors on alcohol consumption. *Addict Behav* 1998;23:101-07. doi: 10.1016/S0306-4603(97)00025-7

54 Legleye S, Baumann M, Peretti-Watel P, et al. Gender and age disparities in the associations of occupational factors with alcohol abuse and smoking in the French working population. *Revue d'Epidemiologie et de Sante Publique* 2011;59:223-32. doi: 10.1016/j.respe.2011.02.103

55 Dobson KG, Ibrahim S, Gilbert-Ouimet M, et al. Association between psychosocial work conditions and latent alcohol consumption trajectories among men and women over a 16-year period in a national Canadian sample. *J Epidemiol Community Health* 2018;72:113-20. doi: 10.1136/jech-2017-209691

56 Skogen JC, Thørrisen MM, Bonsaksen T, et al. Effort-reward imbalance is associated with alcohol-related problems. WIRUS-screening study. *Front Psychol* 2019;10 doi: 10.3389/fpsyg.2019.02079

57 Lips-Wiersma M, Wright S, Dik B. Meaningful work: differences among blue-, pink-, and white-collar occupations. *Career Dev Int* 2016;21;534-51. doi: 10.1108/cdi-04-2016-0052

58 Working Environment Act (Act relating to the environment, working environment, working hours and employment protection, etc.). Oslo, Norway; Norwegian Ministry of Labour and Social Affairs 2005.

59 Alcohol Act (Act on the sale of alcoholic beverages, etc.). Oslo, Norway; Norwegian Ministry of Health and Care Services 1989.

60 Bakken A. Ungdata 2022. Nasjonale resultater [Ungdata 2022. National results]. Oslo, Norway: OsloMet – Oslo Metropolitan University.

61 Tabachnick BG, Fidell LS. Using multivariate statistics. 6th ed. Boston, MA: Pearson Education 2013.

62 Stevens J. Applied multivariate statistics for the social sciences. 3rd ed. Mahwah, NJ: Lawrence Erlbaum 1996.

63 Saunders JB, Aasland OG, Babor TF, et al. Development of the Alcohol Use Disorders Identification Test (AUDIT): WHO collaborative project on early detection of persons with harmful alcohol consumption-II. *Addiction* 1993;88:791-804. doi: 10.1111/j.1360-0443.1993.tb02093.x

64 Bush K, Kivlahan DR, McDonell MB, et al. The AUDIT alcohol consumption questions (AUDIT-C). An effective brief screening test for problem drinking. *Arch Intern Med* 1998;158:1789-95. doi: 10.1001/archinte.158.16.1789

65 Karasek R, Brisson C, Kawakami N, et al. The Job Content Questionnaire (JCQ): An instrument for internationally comparative assessments of psychosocial job characteristics. *J Occup Health Psychol* 1998;3:322-55. doi: 10.1037/1076-8998.3.4.322

66 Johnson JV, Hall EM, Theorell T. Combined effects of job strain and social isolation on cardiovascular disease morbidity and mortality in a random sample of the Swedish male working population. *Scand J Work Environ Health* 1989;15:271-79. doi: 10.5271/sjweh.1852

67 Karasek R. Job demands, job decision latitude, and mental strain: implications for job redesign. *Adm Sci Q* 1979;24:285. doi: 10.2307/2392498

68 Aas RW. Workplace based sick leave prevention and return to work. Exploratory studies (doctoral thesis). Stockholm, Sweden: Karolinska Institute (Department of Clinical Neuroscience) 2011.

For peer review only - http://bmjopen.bmj.com/site/about/guidelines.xhtml

69 Aas RW, Ellingsen KL, Lindøe P, et al. Leadership qualities in the return to work process: a content analysis. *J Occup Rehabil* 2008;18 doi: 10.1007/s10926-008-9147-y

70 Azen R, Budescu DV. The dominance analysis approach for comparing predictors in multiple regression. *Psychol Methods* 2003;8:129-48. doi: 10.1037/1082-989x.8.2.129

71 Neter J, Wasserman W, Kutner MH. Applied linear regression models. Homewood, IL: Irwin 1989.

72 Hosmer DW, Hosmer T, Le Cessie S, et al. A comparison of goodness-of-fit tests for the logistic regression model. *Stat Med* 1997;16;965-80. doi: 10.1002/(sici)1097-0258(19970515)16:9<965::aid-sim509>3.0.co;2-o

73 Budescu DV. Dominance analysis: A new approach to the problem of relative importance of predictors in multiple regression. *Psychol Bull* 1993;114:542-51. doi: 10.1037/0033-2909.114.3.542

74 Ames GM, Janes C. A cultural approach to conceptualizing alcohol and the workplace. *Alcohol Health Res World* 1992;16:112-19.

75 Gordon R, Heim D, MacAskill S. Rethinking drinking cultures: A review of drinking cultures and a reconstructed dimensional approach. *Public Health* 2012;126:3-11. doi: 10.1016/j.puhe.2011.09.014

76 Savic M, Room R, Mugavin J, et al. Defining "drinking culture": A critical review of its meaning and connotation in social research on alcohol problems. *Drugs* 2016;23:270-82. doi: 10.3109/09687637.2016.1153602

77 Nesvåg S, Lie T. Rusmiddelbruk blant ansatte i norsk privat arbeidsliv [Drug use among employees in Norwegian private sector]. *Nord Alkohol Nark* 2004;21:91-109.

78 Nordaune K, Skarpaas LS, Sagvaag H, et al. Who initiates and organises situations for work-related alcohol use? The WIRUS culture study. *Scand J Public Health* 2017;45:749-56. doi: 10.1177/1403494817704109

79 Bellis MA, Hughes K, Nicholls J, et al. The alcohol harm paradox: using a national survey to explore how alcohol may disproportionately impact health in deprived individuals. *BMC Public Health* 2016;16. doi: 10.1186/s12889-016-2766-x

80 Kalodner CR, Delucia JL, Ursprung AW. An examination of the tension reduction hypothesis: The relationship between anxiety and alcohol in college students. *Addict Behav* 1989;14;649-54. doi: 10.1016/0306-4603(89)90007-5

81 Nicolaisen H, Bråthen K. Frivillig deltid - en privatsak? [Voluntary part-time work - a private matter?]. Oslo, Norway: Fafo 2012.

82 Moe MSH, Aas RW, Thørrisen MM. Tid brukt på daglige aktiviteter og alkoholbruk - WIRUS screening studien [Time spent on daily activities and alcohol consumption. The WIRUS screening study]. *Ergoterapeuten* 2020;63:76-87.

83 Carr N. Going with the flow: An assessment of the relationship between young people's leisure and holiday behaviour. *Tourism Geographies* 2002;4:115-34. doi: 10.1080/14616680210124891

84 Heacock RM, Capodilupo ER, Czeisler ME, et al. Sleep and alcohol use patterns during federal holidays in the United States. *MedRxiv* 2021 doi: 10.1101/2021.11.04.21264353

85 Finne LB, Christensen JO, Knardahl S. Psychological and social work factors as predictors of mental distress: a prospective study. *PLoS ONE* 2014;9 doi: 10.1371/journal.pone.0102514

For peer review only

For peer review only - http://bmjopen.bmj.com/site/about/guidelines.xhtml

86 Sterud T, Johannessen HA, Tynes T. Work-related psychosocial and mechanical risk factors for neck/shoulder pain: a 3-year follow-up study of the general working population in Norway. *Int Arch Occup Environ Health* 2014;87:471-81. doi: 10.1007/s00420-013-0886-5

87 Christensen JO, Nielsen MB, Finne LB, et al. Comprehensive profiles of psychological and social work factors as predictors of site-specific and multi-site pain. *Scand J Work Environ Health* 2018;44:291-302. doi: 10.5271/sjweh.3706

88 Felton BJ, Shinn M. Social integration and social support: Moving "social support" beyond the individual level. *J Community Psychol* 1992;20;103-15. doi: 10.1002/1520-6629(199204)20:2<103::aid-jcop2290200202>3.0.co;2-4

89 Bacharach SB, Bamberger P, Cohen A, et al. Retirement, social support, and drinking behavior: A cohort analysis of males with a baseline history of problem drinking. *J Drug Issues* 2007;37;525-48. doi: 10.1177/002204260703700303

90 Thørrisen MM. Alcohol consumption and impaired work performance. Interventions, and implementation barriers (doctoral thesis). Oslo, Norway: OsloMet - Oslo Metropolitan University (Faculty of Health Sciences) 2020.

91 Boniface S, Kneale J, Shelton N. Drinking pattern is more strongly associated with under-reporting of alcohol consumption than socio-demographic factors: evidence from a mixed-methods study. *BMC Public Health* 2014;14 doi: 10.1186/1471-2458-14-1297

92 de Meneses-Gaya C, Zuardi AW, Loureiro SR, et al. Alcohol Use Disorders Identification Test (AUDIT): An updated systematic review of psychometric properties. *Psychol Neurosci* 2009;2:83-97. doi: 10.3922/j.psns.2009.1.12

93 Skogen JC, Thørrisen MM, Olsen E, et al. Evidence for essential unidimensionality of AUDIT and measurement invariance across gender, age and education. Results from the WIRUS study. *Drug Alcohol Depend* 2019;202:87-92. doi: 10.1016/j.drugalcdep.2019.06.002

94 Aalto M, Tuunanen M, Sillanaukee P, et al. Effectiveness of structured questionnaires for screening heavy drinking in middle-aged women. *Alcohol Clin Exp Res* 2006;30:1884-88. doi: 10.1111/j.1530-0277.2006.00233.x

95 Giang KB, Spak F, Dzung TV, et al. The use of AUDIT to assess level of alcohol problems in rural Vietnam. *Alcohol Alcohol* 2005;40:578-83. doi: 10.1093/alcalc/agh198

96 Selin KH. Test-retest reliability of the Alcohol Use Disorder Identification Test in a general population sample. *Alcohol Clin Exp Res* 2003;27:1428-35. doi: 10.1097/01.alc.0000085633.23230.4a

97 Tuunanen M, Aalto M, Seppä K. Binge drinking and its detection among middle-aged men using AUDIT, AUDIT-C and AUDIT-3. *Drug Alcohol Rev* 2007;26:295-99. doi: 10.1080/09595230701247756

98 Lindberg P, Vingård E. Indicators of healthy work environments - a systematic review. *Work* 2012;41:3032-38. doi: 10.3233/wor-2012-0560-3032

99 Nielsen K, Nielsen MB, Ogbonnaya C, et al. Workplace resources to improve both employee well-being and performance: a systematic review and meta-analysis. *Work Stress* 2017;31:101-20. doi: 10.1080/02678373.2017.1304463

100 Burton J. WHO healthy workplace framework and model: background and supporting literature and practice. Geneva, Switzerland: World Health Organization 2010.

For peer review only - http://bmjopen.bmj.com/site/about/guidelines.xhtml

FIGURE TITLE/LEGEND

Figure 1. Overview of main study results. Percentages reflect relative importance of predictors based on dominance analysis (average contribution of the predictor to the outcome in percentage points when analysed in conjunction with other workplace factors that are significantly associated with the outcome)

For peer review only

For peer review only - http://bmjopen.bmj.com/site/about/guidelines.xhtml

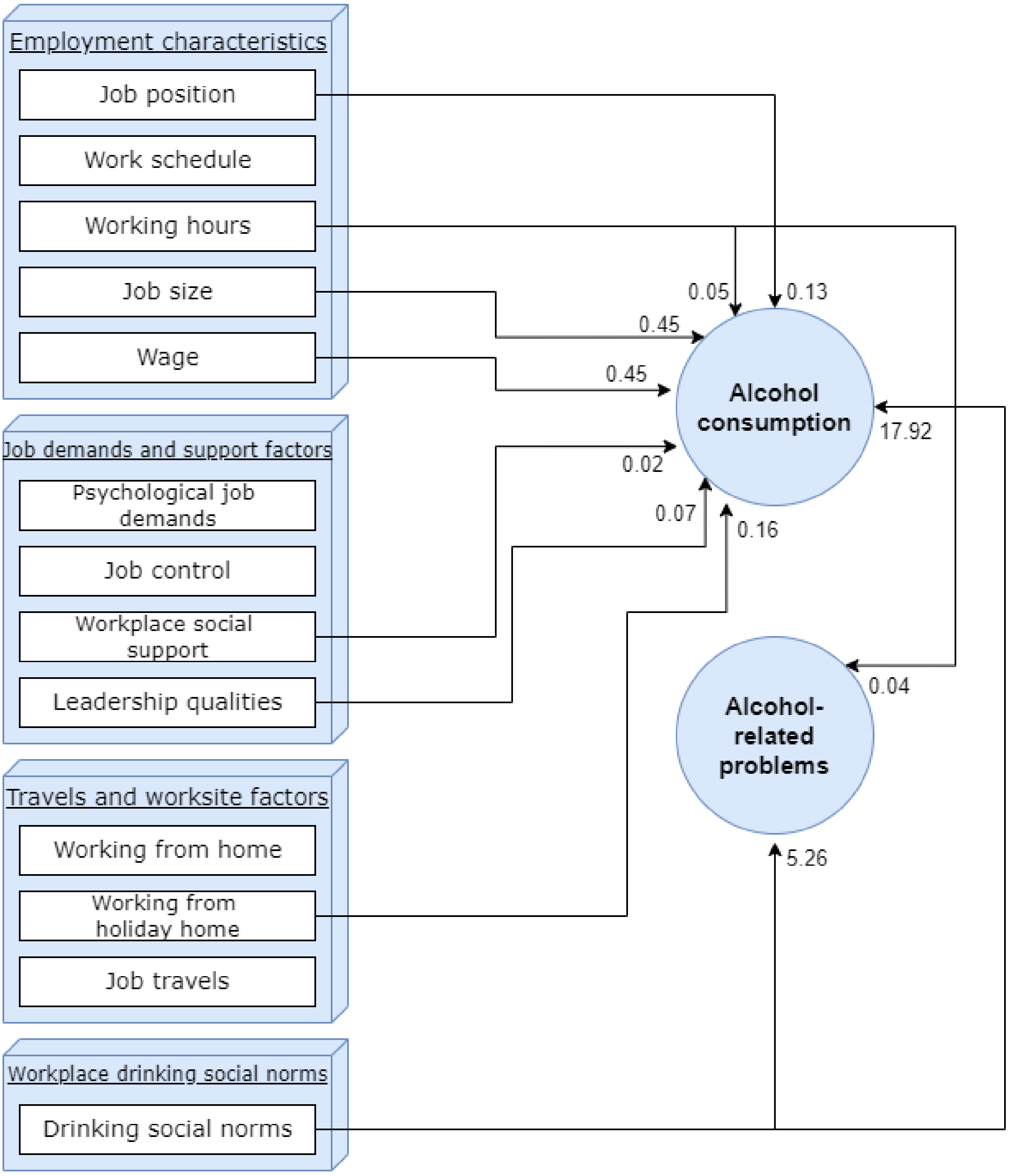
Employment characteristics
Job position
Work schedule
Working hours
Job size
Wage
Job demands and support factors
Psychological job demands
Job control
Workplace social support
Leadership qualities
Travels and worksite factors
Working from home
Working from holiday home
Job travels
Workplace drinking social norms
Drinking social norms
Alcohol consumption
Alcohol-related problems
0.05
0.13
0.45
0.45
0.02
0.07
0.16
17.92
0.04
5.26

Supplementary File

Table S1. Matrix of bivariate correlations between all study variables (outcomes, predictors and covariates)

	1	2	3	4	5	6	7	8	9	10	11	12	13	14	15	16	17	18	19	20	21	22	23	24	25
1. Alcohol consumption																									
2. Alcohol-rel. problems[A]	**.49**																								
3. Working hours	.02	-.01																							
4. Day job, no weekends[B]	**-.05**	**-.05**	.02																						
5. Evening job[B]	.01	.02	**-.05**	**-.11**																					
6. Night job[B]	-.01	.01	-.01	**-.21**	-.01																				
7. Shift work[B]	**.04**	**.04**	**-.08**	**-.79**	**-.03**	**-.06**																			
8. Job position[C]	**.04**	**-.03**	**.24**	**.12**	**-.03**	**-.06**	**-.14**																		
9. Job size[D]	**.08**	**.04**	**.25**	**.20**	**-.10**	**-.25**	**-.15**	**.19**																	
10. Income	.01	**-.08**	**.14**	**.13**	**-.04**	**-.05**	**-.13**	**.21**	**.11**																
11. Psych. job demands	-.02	-.00	**.23**	**-.05**	-.00	**-.06**	.03	**.17**	**.07**	**.05**															
12. Job control	-.01	**-.04**	**.15**	**.10**	-.01	**-.08**	**-.12**	**.14**	**.08**	**.17**	**.14**														
13. Work. social support	.01	**-.03**	.00	**.06**	-.01	**-.03**	**-.05**	.00	-.00	**.05**	**-.06**	**.34**													
14. Leadership qualities	-.02	**-.03**	-.02	.02	-.01	**-.04**	-.01	-.01	-.01	.01	**-.10**	**.23**	**.61**												
15. Job travels[E]	**.07**	.02	**.13**	**.09**	-.02	**-.05**	**-.11**	**.16**	**.13**	**.15**	**-05**	**.14**	.01	-.01											
16. Working from home[E]	.02	-.00	**.12**	**.04**	-.01	**-.03**	**-.09**	**.11**	**.04**	**.10**	**.06**	**.13**	.01	-.01	**.25**										
17. Work. holiday home[E]	**.06**	.01	**.12**	-.03	-.01	-.02	-.02	**.10**	**.05**	**.11**	**.07**	**.06**	-.02	-.02	**.24**	**.32**									
18. Drinking social norms[F]	**.42**	**.23**	**.06**	**-.05**	.02	-.03	**.04**	**-.05**	**.07**	.02	-.01	-.01	.00	.01	**.09**	**.06**	**.03**								
19. Gender[G]	**-.18**	**-.14**	**-.16**	**.04**	.01	.02	-.02	**-.13**	**-.17**	-.02	**.05**	.01	**.06**	**.05**	**-.17**	**-.03**	**-.08**	**-.16**							
20. Age	**-.12**	**-.13**	**.05**	**.19**	-.01	-.01	**-.20**	**.18**	.00	**.10**	**-.07**	-.02	**-.09**	**-.10**	.03	-.02	**.05**	**-.25**	**-.04**						
21. Educational attainment	**-.03**	-.03	**.18**	**.15**	-.03	**-.07**	**-.18**	**.09**	**.13**	**.29**	**.10**	**.30**	**.08**	**.04**	**.16**	**.14**	**.06**	**.06**	.01	**-.07**					
22. Living status[H]	**-.07**	**-.12**	.02	**.07**	-.01	-.02	**-.06**	**.06**	-.02	**.50**	.01	**.07**	**.03**	.03	**.03**	.01	**.03**	**-.04**	-.01	-.02	**.03**				
23. Marital status[I]	**-.14**	**-.15**	**.04**	**.14**	-.01	-.02	**-.15**	**.13**	-.02	**.46**	.00	**.08**	.01	-.00	**.06**	**.03**	**.04**	**-.12**	**-.04**	**.26**	**.07**	**.46**			
24. Number of children	**-.16**	**-.17**	**.03**	**.15**	-.02	-.01	**-.15**	**.14**	-.02	**.31**	-.01	**.07**	-.01	-.01	**.06**	**.04**	**.04**	**-.20**	.00	**.40**	.01	**.33**	**.42**		
25. Year of data collection	.01	.01	**-.10**	**-.12**	**.04**	**.04**	**.16**	.00	**-.04**	**-.07**	.02	**.06**	**.07**	**.09**	**-.15**	**-.06**	-.01	**-.04**	**.14**	**-.07**	**.04**	-.02	**-.03**	-.02	

N = 5388; Results from non-parametric (Spearman *rho*) correlation analyses; Statistically significant correlations (p <.05) indicated with bold typeface; [A]Ref. = no problems; [B]Ref. = other work schedules; [C]Ref. = worker; [D]Ref. = part-time; [E]Ref. = no; [F]Higher scores indicate liberal attitudes; [G]Ref. = male; [H]Ref. = living alone; [I]Ref. = unmarried; For all other variables: higher scores indicate higher variable values

For peer review only

Table S2. Study selection analyses: Comparisons between completers and non-completers on sociodemographic variables

Variable	Completers[A]	Non-completers[B]	*p*
Gender, % females	68.5	75.7	<.001[C]
Age, *M*	44.8	44.7	.946[D]
Education, % university/college	77.7	66.2	<.001[C]
Livings status, % living alone	15.1	15.1	.949[C]
Marital status, % unmarried	44.8	48.0	.005[C]
Children, *M*	1.8	1.7	.001[D]

[A]Employees who responded on all study items, i.e., the study sample (n = 5388); [B]Employees who responded on the sociodemographic items but not on all study items; [C]Difference tested with chi square tests of independence; [D]Difference tested with independent samples t-tests

For peer review only

STROBE Statement

Checklist of items that should be included in reports of observational studies

Section/Topic	Item No	Recommendation	Reported on Page No
**Title and abstract**	1	(*a*) Indicate the study's design with a commonly used term in the title or the abstract	1,2
		(*b*) Provide in the abstract an informative and balanced summary of what was done and what was found	2,3
**Introduction**			
Background/rationale	2	Explain the scientific background and rationale for the investigation being reported	3-8
Objectives	3	State specific objectives, including any prespecified hypotheses	8
**Methods**			
Study design	4	Present key elements of study design early in the paper	8
Setting	5	Describe the setting, locations, and relevant dates, including periods of recruitment, exposure, follow-up, and data collection	8-10
Participants	6	(*a*) *Cohort study*—Give the eligibility criteria, and the sources and methods of selection of participants. Describe methods of follow-up<br>*Case-control study*—Give the eligibility criteria, and the sources and methods of case ascertainment and control selection. Give the rationale for the choice of cases and controls<br>*Cross-sectional study*—Give the eligibility criteria, and the sources and methods of selection of participants	8-10
		(*b*) *Cohort study*—For matched studies, give matching criteria and number of exposed and unexposed<br>*Case-control study*—For matched studies, give matching criteria and the number of controls per case	
Variables	7	Clearly define all outcomes, exposures, predictors, potential confounders, and effect modifiers. Give diagnostic criteria, if applicable	10-13
Data sources/measurement	8*	For each variable of interest, give sources of data and details of methods of assessment (measurement). Describe comparability of assessment methods if there is more than one group	10-13
Bias	9	Describe any efforts to address potential sources of bias	12,13
Study size	10	Explain how the study size was arrived at	10
Quantitative variables	11	Explain how quantitative variables were handled in the analyses. If applicable, describe which groupings were chosen and why	10-13
Statistical methods	12	(*a*) Describe all statistical methods, including those used to control for confounding	13,14
		(*b*) Describe any methods used to examine subgroups and interactions	n/a
		(*c*) Explain how missing data were addressed	Suppl.
		(*d*) *Cohort study*—If applicable, explain how loss to follow-up was addressed<br>*Case-control study*—If applicable, explain how matching of cases and controls was addressed<br>*Cross-sectional study*—If applicable, describe analytical methods taking account of sampling strategy	n/a
		(*e*) Describe any sensitivity analyses	Suppl.

For peer review only - http://bmjopen.bmj.com/site/about/guidelines.xhtml

Section/Topic	Item No	Recommendation	Reported on Page No
**Results**			
Participants	13*	(a) Report numbers of individuals at each stage of study—eg numbers potentially eligible, examined for eligibility, confirmed eligible, included in the study, completing follow-up, and analysed	9
		(b) Give reasons for non-participation at each stage	n/a
		(c) Consider use of a flow diagram	n/a
Descriptive data	14*	(a) Give characteristics of study participants (eg demographic, clinical, social) and information on exposures and potential confounders	9,10
		(b) Indicate number of participants with missing data for each variable of interest	n/a
		(c) *Cohort study*—Summarise follow-up time (eg, average and total amount)	
Outcome data	15*	*Cohort study*—Report numbers of outcome events or summary measures over time	
		Case-control study—Report numbers in each exposure category, or summary measures of exposure	
		Cross-sectional study—Report numbers of outcome events or summary measures	10
Main results	16	(*a*) Give unadjusted estimates and, if applicable, confounder-adjusted estimates and their precision (eg, 95% confidence interval). Make clear which confounders were adjusted for and why they were included	14-22
		(*b*) Report category boundaries when continuous variables were categorized	n/a
		(*c*) If relevant, consider translating estimates of relative risk into absolute risk for a meaningful time period	n/a
Other analyses	17	Report other analyses done—eg analyses of subgroups and interactions, and sensitivity analyses	Suppl.
**Discussion**			
Key results	18	Summarise key results with reference to study objectives	23
Limitations	19	Discuss limitations of the study, taking into account sources of potential bias or imprecision. Discuss both direction and magnitude of any potential bias	27-29
Interpretation	20	Give a cautious overall interpretation of results considering objectives, limitations, multiplicity of analyses, results from similar studies, and other relevant evidence	23-27
Generalisability	21	Discuss the generalisability (external validity) of the study results	27,28
**Other Information**			
Funding	22	Give the source of funding and the role of the funders for the present study and, if applicable, for the original study on which the present article is based	31

**Give information separately for cases and controls in case-control studies and, if applicable, for exposed and unexposed groups in cohort and cross-sectional studies.*

Note: An Explanation and Elaboration article discusses each checklist item and gives methodological background and published examples of transparent reporting. The STROBE checklist is best used in conjunction with this article (freely available on the Web sites of PLoS Medicine at http://www.plosmedicine.org/, Annals of Internal Medicine at http://www.annals.org/, and Epidemiology at http://www.epidem.com/). Information on the STROBE Initiative is available at www.strobe-statement.org.

For peer review only - http://bmjopen.bmj.com/site/about/guidelines.xhtml

For peer review only
